# DJ-1 counteracts Caveolin-1-mediated necroptosis to inhibit epithelial barrier dysfunction in colitis

**DOI:** 10.1038/s41419-025-07989-z

**Published:** 2025-08-29

**Authors:** Mengli Yu, Jie Zhang, Bingru Lin, Wei Zhu, Xin Song, Jiaqi Pan, Dingwu Li, Xinjue He, Jing Sun, Zhe Shen, Chaohui Yu

**Affiliations:** 1 Department of Gastroenterology, the Fourth Affiliated Hospital of School of Medicine, and International School of Medicine, International Institutes of Medicine, Zhejiang University, Yiwu, China; 2 Department of Gastroenterology, the First Affiliated Hospital, College of Medicine, Zhejiang University, Hangzhou, China; 3 Department of Gastroenterology, Ruijin Hospital, Shanghai Jiao Tong University School of Medicine, Shanghai, China

**Keywords:** Ulcerative colitis, Cell death and immune response, Necroptosis

## Abstract

Caveolin-1 (CAV1), a pivotal protein implicated in endothelial cell-mediated angiogenesis, assumes an ambiguous role with elusive underlying mechanisms in the pathogenesis of inflammatory bowel disease (IBD). In this investigation, we delineated the involvement of CAV1 in murine models of dextran sulfate sodium (DSS)-induced colitis. CAV1 knockout mice manifested attenuated pathological and inflammatory damage to the epithelium, whereas mice overexpressing CAV1 exhibited contrasting outcomes. In vivo, the accumulation of epithelial CAV1 contributed to the disruption of the epithelial barrier by promoting necroptosis. Subsequent mechanistic analyses revealed that the colitis-protective protein DJ-1 regulated CAV1 through a proteasome-mediated protein degradation pathway. Utilizing necroptosis-modeled organoids from murine intestines and pharmacological inhibition of necroptosis, our findings demonstrated that the DJ-1/CAV1 pathway governed epithelial inflammation via necroptosis in the context of colitis. In summary, our research revealed that epithelial CAV1 aggravated necroptosis in experimental colitis, leading to impairment of the epithelial barrier, which was negatively regulated by DJ-1.

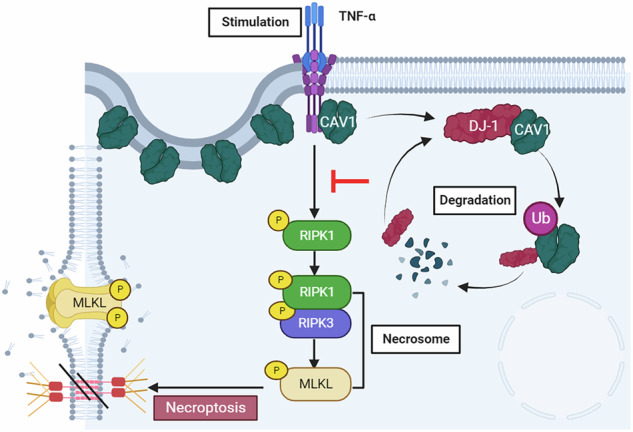

## Introduction

Inflammatory bowel disease (IBD), which encompasses ulcerative colitis (UC) and Crohn’s disease (CD), is a gastrointestinal disorder characterized by inflammation of the digestive tract, with its exact cause remaining unknown [1]. IBD has evolved into a global burden given its increasing incidence worldwide [2, 3]. However, the underlying pathogenetic mechanism is still unclear, and effective treatments are limited. Hence, there is a requirement for the advancement of fresh medications and innovative approaches to treatment in order to cater to individualized healthcare needs.

Caveolin-1 (CAV1) is a scaffolding structural protein of caveolae and was reported to participate in the regulation of angiogenesis, cell signaling and endothelial-mediated inflammation [4, 5]. CAV1 serves as the principal architectural constituent of caveolae in endothelial cells, and it has been documented that endothelial CAV1 plays a pivotal role in regulating colitis [6]. Except for angiogenesis, CAV1 has been demonstrated to participate in tumor necrosis factor receptor 1 (TNFR1) signaling, which is implicated in inflammation response during IBD as reported [7]. However, in contrast to its role in the endothelium, CAV1 deficiency has been shown to exacerbate DSS-induced colitis and contribute to the prevention of intestinal nitrosative stress and mucosal barrier damage [8, 9]. And other studies claimed that epithelial expression of CAV2, but not CAV1, was enhanced in the inflamed mucosa of patients with ulcerative colitis (UC) [10]. Thus, CAV1 plays a pivotal role in the pathogenesis of colitis; however, its precise function remains subject to debate and ambiguity.

Parkinson disease 7 (PARK7 or DJ-1) is a protein that was shown to have antiapoptotic effects in our previous study [11–14]. We also found that the protective effect of DJ-1 in IBD was possibly related to CAV1 [11]. Hence, to further determine the role of CAV1 in IBD and explore its relationship with DJ-1, we carried out this research.

Necroptosis is a caspase-independent programmed cell death executed by the activation of death receptors [15]. Generally, necroptosis is recognized as proinflammatory cell death and includes three key modulators, receptor interacting protein kinases (RIPK)-1, RIPK3 and mixed lineage kinase domain like protein (MLKL) [16, 17]. The severity of inflammation in experimental colitis was found to be exacerbated by a defect in either RIPK3 [18] or MLKL [19], leading to necroptosis. Further, clinical specimen detection strongly indicated that necroptosis was closely associated with intestinal inflammation in children with IBD [20]. Hence, we posit that comprehending the molecular mechanisms underlying necroptosis and its implication in colitis holds significant interest for the management of human IBD.

Here, we aimed to address the question of the role of CAV1 in colitis and whether the pathogenesis of CAV1 is dependent on necroptosis. We postulated that CAV1 played a vital role in intestinal inflammation and that DJ-1 and necroptosis were involved. To test this hypothesis, we constructed CAV1 or DJ-1 knockout transgenic mice and generated the double knockout mice (DKO) through hybridization. We conducted both in vivo and in vitro experiments, which included intestinal organoids culture. This pathway could potentially be an unrecognized mechanism of CAV1 and shed light on the diagnosis and therapies of colitis.

## Results

### Intestinal CAV1 expression was increased in IBD patients and DSS-treated mice

**Fig. 1:**
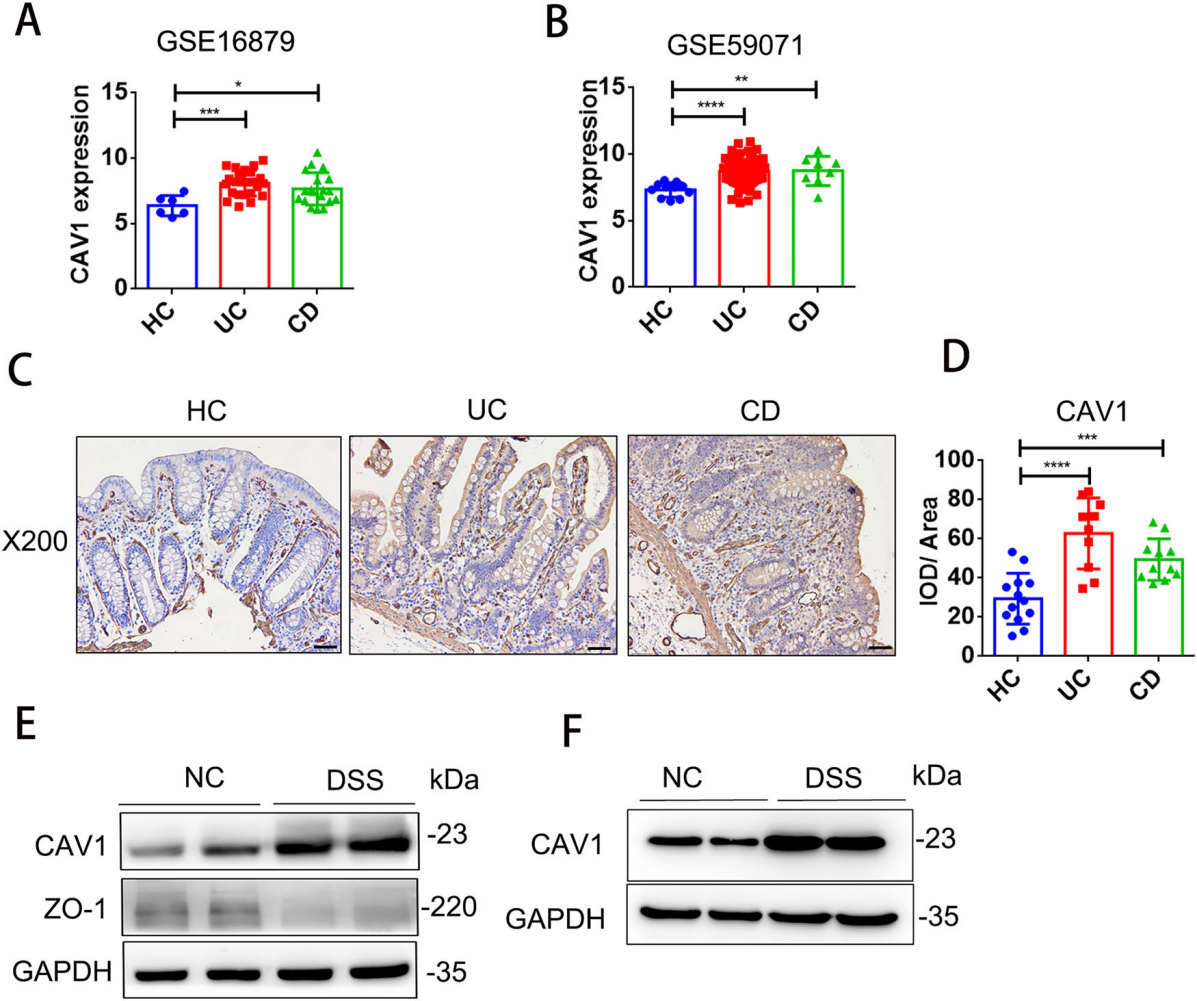
Intestinal CAV1 expression was increased in IBD patients and DSS-treated mice. **A**, **B** Bioinformatics analysis of CAV1 mRNA expression in the GEO database, **A** GSE16879 containing 6 healthy controls (HC), 24 UC patients and 19 CD patients before infliximab treatment, **B** GSE59071 containing 11 healthy controls, 97 UC patients and 8 CD patients. **C** Representative IHC analysis of CAV1 expression in colonic tissue samples from the healthy controls and the patients with UC or CD (magnification ×200; scale bar = 50 µm). **D** Statistical analysis of CAV1 expression in the healthy controls (*n* = 14), patients with UC (*n* = 10), and patients with CD (*n* = 12). **E**, **F** Western blotting of CAV1 protein expression in the control mice and the DSS-treated mice. Whole colon tissue (**E**) and the isolated colonic epithelium (**F**) were shown. All data are the means ± SD. *p < 0.05, **p < 0.01, ***p < 0.001, ****p < 0.0001, two-tailed.

Firstly, two GEO datasets, GSE16879 and GSE59071, with a total of 148 inflamed colonic tissues from UC or CD patients and 17 healthy colonic tissues, were used. All CAV1 mRNA levels were elevated in UC and CD colonic tissues compared with healthy tissues (Fig. 1A, B). Then, we found that CAV1 protein expression was markedly increased in the samples from both UC and CD patients compared with those of the healthy control subjects (Fig. 1C), as indicated by semiquantitative analysis of the integral optical density (IOD) of the CAV1–positive areas (Fig. 1D). Moreover, in the mice with DSS-induced colitis, there was an accumulation of CAV1 protein expression compared to that in the control mice (Fig. 1E). Then, we isolated the colonic epithelium of the DSS-treated mice and found that CAV1 significantly accumulated in the colonic epithelium (Fig. 1F). These results suggested that CAV1 expression was increased in IBD.

### CAV1 deficiency alleviated DSS-induced experimental colitis

**Fig. 2:**
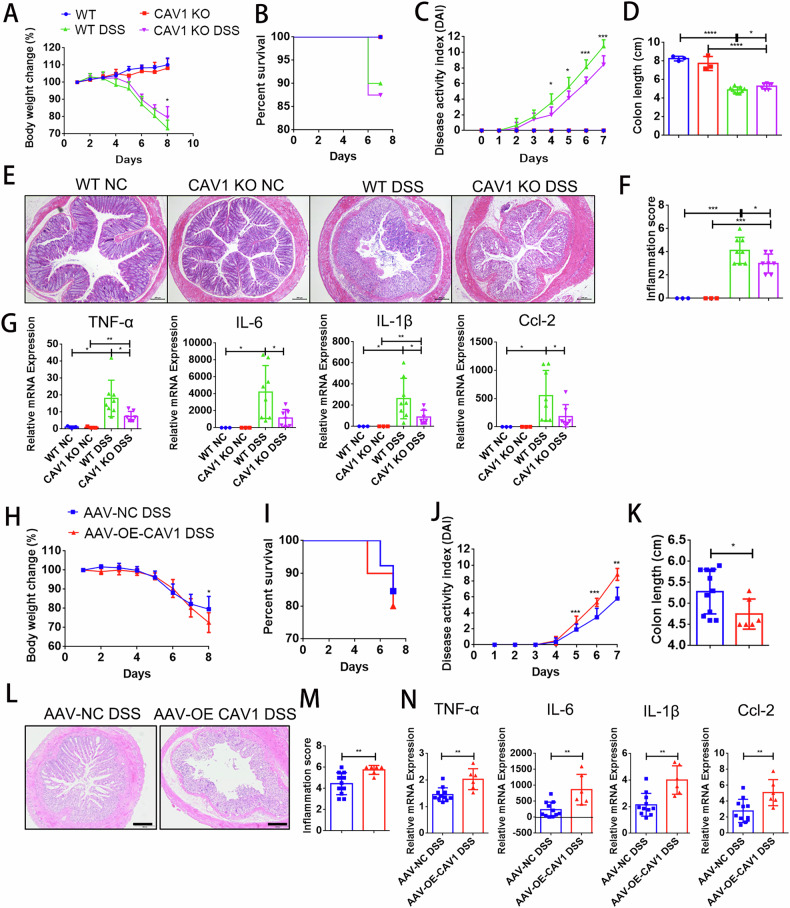
CAV1 promoted DSS-induced experimental colitis. **A**–**G** WT and CAV1 KO mice were treated with 3.5% DSS for 7 days. WT NC = 3, CAV1 KO NC = 3, WT DSS = 8, CAV1 KO DSS = 7. Body weight change (**A**), survival rates (**B**), the disease activity index (DAI) (**C**) and colon length (**D**) were calculated. Mice were sacrificed on Day 7, and (**E**) histological changes in colon tissue samples from the WT and CAV1 KO mice were measured (magnification ×100; scale bar=100 µm). **F** Semiquantitative scoring of histopathology was performed. **G** Quantitative PCR analysis of cytokines and chemokines in colons from the DSS-treated mice. **H**–**N** Male C57BL/6 mice were infected with RFP-tagged CAV1-overexpressing AAV7 (AAV-OE-CAV1) or the negative control AAV7 (AAV-NC) then administered DSS for 7 days. AAV-NC DSS = 11; AAV-OE CAV1 DSS = 6. Body weight change (**H**), survival rates (**I**), DAI scores (**J**), colon length (**K**) were measured. **L** Histological changes are shown (magnification ×100; scale bar=200 µm). **M** Semiquantitative scoring of histopathology was performed, and **N** quantitative PCR analysis of inflammatory markers was calculated. DAI scores and inflammation scores are expressed as median and IQR. Other All data are the means ± SD. *p < 0.05, **p < 0.01, ***p < 0.001, ****p < 0.0001, two-tailed.

Several studies have reported generation of DSS colitis models in CAV1 KO mice, but the results are controversial [6, 8]. To confirm the role of CAV1 in colitis, we repeated the same experiment. CAV1 KO mice and their WT littermates were treated with 3.5% DSS to establish an acute experimental colitis model [21]. The CAV1 KO DSS-treated mice had a higher body weight and lower disease activity index (DAI) scores than their WT DSS-treated littermates (Fig. 2A, C), although CAV1 deficiency did not decrease the death rate after DSS administration (Fig. 2B). In addition, intestinal analysis showed that CAV1 deficiency reversed the DSS-induced shortened colon length and hematochezia (Fig. 2D, Supplementary Fig. S1A). The histological examination results corresponded with the clinical observations (Fig. 2E, F). Compared with the WT mice, the KO mice exhibited milder disruption of the epithelial architecture, with smaller areas of crypt loss and much less inflammation. Colonic proinflammatory cytokines (tumor necrosis factor (Tnf)-α, interleukin (IL)-6 and IL-1β) and Ccl-2 were activated in the WT DSS-treated mice and were decreased in the CAV1 KO DSS-induced mice (Fig. 2G).

### Injection of CAV1-overexpressing AAV aggravated DSS-induced experimental colitis

Then, we tested the effect of CAV1 in IBD through gain-of-function experiments. Tail intravenous injection of RFP-tagged CAV1-overexpressing AAV7 was applied to upregulate intestinal CAV1 protein expression. Immunofluorescence showed that the RFP-tagged CAV1 protein was successfully expressed in the colonic epithelium (Supplementary Fig. S1B). Western blot analysis and quantitative PCR analysis both showed that the total expression of CAV1 in the colon was significantly higher than that of control AAV7 (Supplementary Fig. S1C–E). Based on the protein expression levels of CAV1 in the colon, mice that successfully achieved intestinal overexpression of CAV1 were subjected to statistical analysis. Although the survival rate was similar, the body weight loss, DAI scores and colon length shortening were significantly aggravated in the AAV-OE-CAV1 DSS-treated mice compared with the AAV-NC DSS-treated mice (Fig. 2H–K, Supplementary Fig. S1F). In accordance with the elevated inflammation scores, the expression levels of proinflammatory cytokines were significantly increased in the colons of the CAV1-overexpressing mice (Fig. 2L–N). Consistent with Kevil et al. [6], our clinical and histological results both indicated that CAV1 deficiency markedly alleviated DSS-induced colitis in mice.

### Absence of CAV1 in non-hematopoietic cells contributed to DSS-induced experimental colitis

However, Kevil et al. mainly focused on the effect of CAV1 in the endothelium [6], and we found that CAV1 also accumulated in the epithelium. To determine the cell populations that contribute to CAV1-mediated inflammation in colitis, we generated CAV1 KO bone marrow chimeras (Supplementary Fig. S2A). WT recipient mice received a dose of γ-ray irradiation (7.5 Gy) to kill the bone marrow cells and were immediately injected intravenously with bone marrow cells from donor WT mice or CAV1 KO mice. Three weeks after bone marrow reconstitution, the genotypes of the 2 groups were evaluated (Supplementary Fig. S2B), and the mice were subjected to DSS after another 3 weeks. Although the CAV1 KO to WT mice had a lower survival rate (Supplementary Fig. S2D), the WT to WT mice, in which CAV1 was intact in all cells, had similar body weights, DAI scores, colon length, histological scores and proinflammatory cytokines and chemokines as the CAV1 KO to WT mice, in which CAV1 was disrupted in myeloid cells (Supplementary Fig. S2C, E–I). Collectively, these data indicated that non-hematopoietic cells may play a predominant role in the colitis pathogenesis.

### The accumulation of CAV1 disrupted the epithelial barrier by promoting necroptosis in vitro

**Fig. 3:**
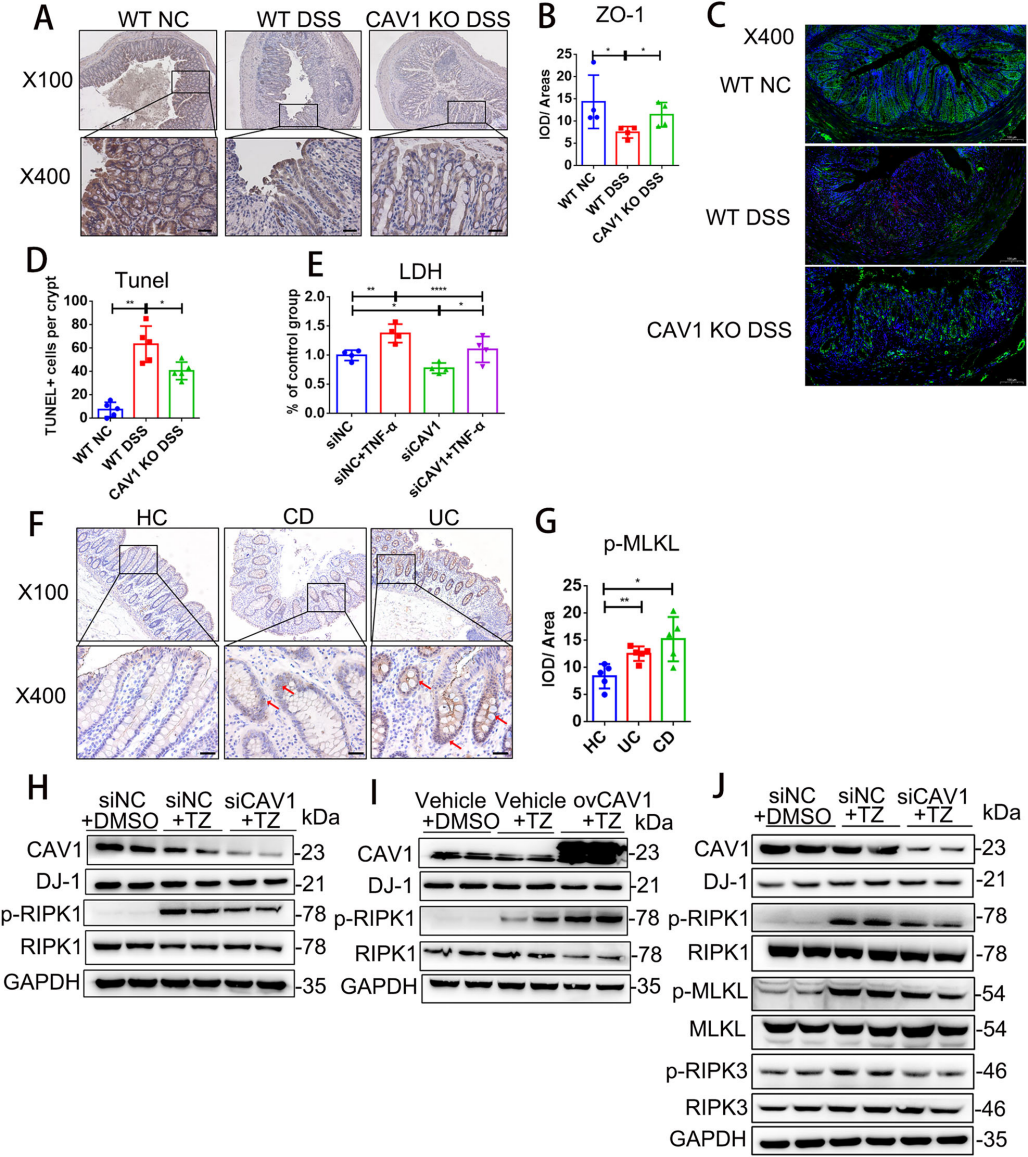
CAV1 disrupted intestinal barrier function by promoting epithelial necroptosis. **A** Representative IHC analysis of ZO-1 expression in colonic tissue samples from the WT and CAV1 KO mice (upper: magnification ×100；scale bar = 100 µm, lower: magnification ×400；scale bar = 20 µm). **B** Quantification of IHC quantification analysis of ZO-1 (*n* = 4) expression. **C** TUNEL staining and IHC staining of ZO-1 in colonic tissue samples from the above groups (TUNEL magnification ×400, scale bar=100 µm). **D** Quantification of TUNEL (green) staining and nuclear DAPI (blue) (*n* = 4). **E** LDH levels in culture medium supernatant were measured in the CAV1 knockdown HCT116 cells after stimulation with 100 ng/ml TNF-α for 24 h (*n* = 4). **F** Representative IHC analysis of p-MLKL expression in colonic tissue samples from the healthy controls (HC) and the patients with UC or CD (upper: magnification ×100; scale bar = 100 µm, lower: magnification ×400; scale bar = 20 µm). **G** Statistical analysis of p-MLKL expression (*n* = 5). CAV1 was knocked down (**H**) or overexpressed (**I**) for 24 h in HCT116 cells, and then, necroptosis was induced with 100 ng/ml TNF-α and 25 µM zVAD-fmk (TZ) for 24 h. An equal amount of DMSO was added as the solvent control. **J** Western blot analysis of necroptosis marker expression in HT29 cells after CAV1 knockdown and TZ stimulation. All data are the means ± SD. *p < 0.05, **p < 0.01, ****p < 0.0001, two-tailed.

Then, we further examined the role of CAV1 in the epithelium. Compared with the WT mice, the reduction in expression of the epithelial barrier marker protein ZO-1 was less pronounced in DSS-induced experimental colitis mice with CAV1 deficiency mice (Fig. 3A, B). And TUNEL staining, which detects all forms of cell death, revealed that CAV1 deficiency suppressed epithelial death (Fig. 3C, D). Consistent with animal experiments, in vitro, we found the lactate dehydrogenase (LDH) level in the culture medium was significantly decreased in the CAV1-silenced group (Fig. 3E). After the overexpression of CAV1, proinflammatory cytokines, such as TNF-α, IL-8, IL-1β and intercellular adhesion molecule (ICAM)-1, were significantly increased (Supplementary Fig. S3A). In contrast, CAV1 knockdown resulted in decreased levels of the proinflammatory cytokines under TNF-α stimulation (Supplementary Fig. S3B). We investigated multiple indicators of cell death modality and observed no differential expression in cleaved-caspase3 (an apoptosis marker) [22], cleaved-GSDMD (a pyroptosis marker) [23], and GPX4 (markers of ferroptosis) [24, 25] between the CAV1 knockout group and WT group. However, we noted an elevation in p-MLKL levels in CAV1-deficient colitis mice, suggesting a potential link between CAV1-induced epithelial demise and necroptosis (Supplementary Fig. S3C). Necroptosis is modulated by the kinases RIPK1 and RIPK3 and the pseudokinase MLKL [26]. We found that p-MLKL was significantly increased in the colons of CD and UC patients (Fig. 3F, G). Therefore, we suspected that CAV1-regulated intestinal epithelial inflammation was related to the necroptosis pathway. We applied a combination of TNF-α and zVAD-fmk, a pancaspase inhibitor, to generate a necroptosis model in vitro. The data showed that CAV1 knockdown decreased the expression of p-RIPK1 in the necroptosis cell model (Fig. 3H). In contrast, CAV1 overexpression aggravated the expression of p-RIPK1 (Fig. 3I). However, we failed to detect p-RIPK3 and p-MLKL, the other two markers of necroptosis, in HCT116 cells. Therefore, we used another intestinal epithelial tumor cell line, HT29, and stimulated it with TZ. Consistent with our observations in HTC116 cells, CAV1 deficiency significantly alleviated the changes in all necroptosis markers (Fig. 3J). Collectively, these findings suggested that epithelial CAV1 promoted necroptosis in the epithelium, thereby exacerbating disruption of the epithelial barrier in IBD.

### The expression of CAV1 was regulated by the colitis protective protein DJ-1 via a proteasome-mediated protein degradation pathway

**Fig. 4:**
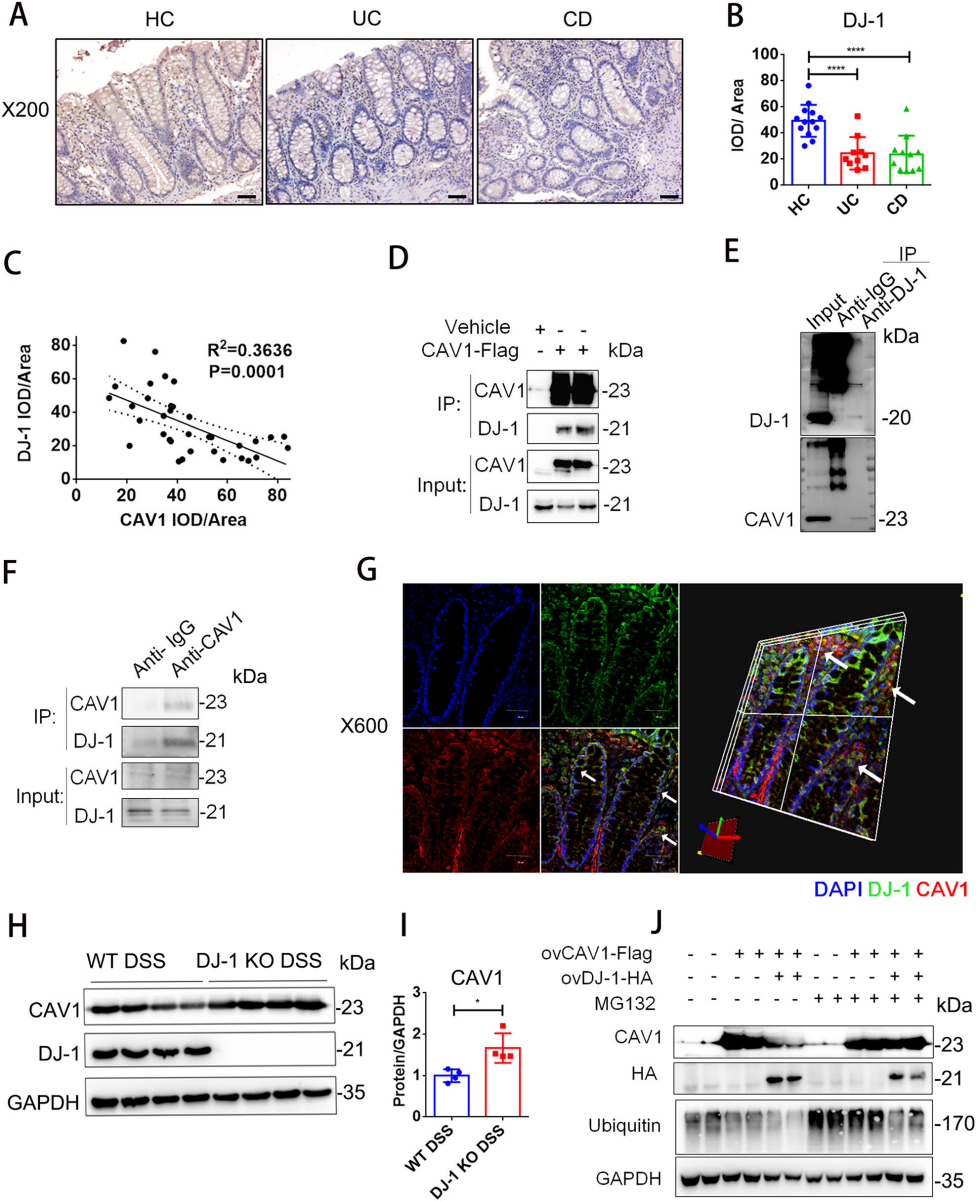
DJ-1 was required for CAV1 degradation through direct protein-protein interactions. **A** Representative IHC analysis of DJ-1 expression in colonic tissue samples from healthy controls and patients with UC or CD (magnification ×200; scale bar = 50 µm). **B** Statistical analysis of DJ-1 expression in the healthy controls (*n* = 13), patients with UC (*n* = 10) or CD (*n* = 11). **C** Correlation analysis of the CAV1 and DJ-1 IHC staining IOD/area score is shown (*n* = 36). **D** HCT116 cells were infected with a FLAG-tagged CAV1 overexpression plasmid or the empty vector (vehicle), and total FLAG-tagged CAV1 was immunoprecipitated. **E** Endogenous co-IP: HEK-293 cell lysates were immunoprecipitated with anti–DJ-1 or control IgG antibodies. **F** Endogenous co-IP: HT29 cell lysates were immunoprecipitated with anti–DJ-1 or control IgG antibodies. **G** Representative images of immunofluorescence staining of IBD human colon sections (DJ-1: green, CAV1: red, DAPI nuclear: blue, magnification ×600). **H** Western blot analysis of colonic CAV1 protein levels in the WT and DJ-1 KO mice with DSS-induced colitis. **I** Quantitative analysis of the above CAV1 protein levels (*n* = 4). **J** HEK293 cells infected with FLAG-tagged CAV1, HA-tagged DJ-1 plasmid and empty vector were stimulated with 100 ng/ml TNF-α and 25 µM zVAD-fmk for 24 h and then treated with MG132 (25 µM, I) for another 6 h. All data are the means ± SD. *p < 0.05, ***p < 0.001, ****p < 0.0001, two-tailed.

To further investigate the upstream mechanisms regulating CAV1 expression, we overexpressed Flag-tagged CAV1 in HEK293 cells and obtained the proteins that interacted with CAV1 by pulldown experiments. Specific peptides of DJ-1 were found through protein profile analysis (Supplementary Fig. S4A). Then, we examined DJ-1 protein levels again in our collected human colon samples, and the IBD colonic tissues showed a determinate decline compared with those of the healthy controls (Fig. 4A, B). Semiquantitative analysis of the IOD indicated that CAV1 expression was negatively associated with DJ-1 expression (Fig. 4C). The protein profile result was further confirmed by coimmunoprecipitation (co-IP), demonstrating a significant interaction between DJ-1 and CAV1 under both endogenous and exogenous conditions (Fig. 4D–F, Supplementary Fig. S4B). Additionally, immunofluorescence staining revealed colocalization of DJ-1 and CAV1 in UC colonic tissue (Fig. 4G). Further exploration found that the CAV1 protein level in the DJ-1 KO DSS-treated mice was much higher than that in the WT DSS-treated mice (Fig. 4H, I). However, intervention with DJ-1 expression in vitro did not affect CAV1 transcriptional levels (Supplementary Fig. S4C), illustrating that DJ-1 deficiency only promoted CAV1 protein levels. In addition, the DJ-1 expression in the CAV1 KO mice was equal to that in the WT DSS-treated mice (Supplementary Fig. S4D), suggesting that DJ-1 was an upstream molecule of CAV1. Then, the cells were treated with the proteasome inhibitor MG132 or autophagy inhibitor 3-MA, and the results showed that MG132 (Fig. 4J) prevented the downregulation of CAV1 in the presence of DJ-1 overexpression while 3-MA made the inhibitory effect of DJ-1 on CAV1 protein persisted (Supplementary Fig. S4E). Additionally, overexpression of DJ-1 promoted the ubiquitination of the CAV1 protein (Supplementary Fig. S4F). According to the above evidence, we demonstrated that DJ-1 deficiency decreased CAV1 degradation in epithelial cells through physical protein interactions and proteasome pathway.

### CAV1 knockout rescued DSS-induced colitis in DJ-1-deficient mice

**Fig. 5:**
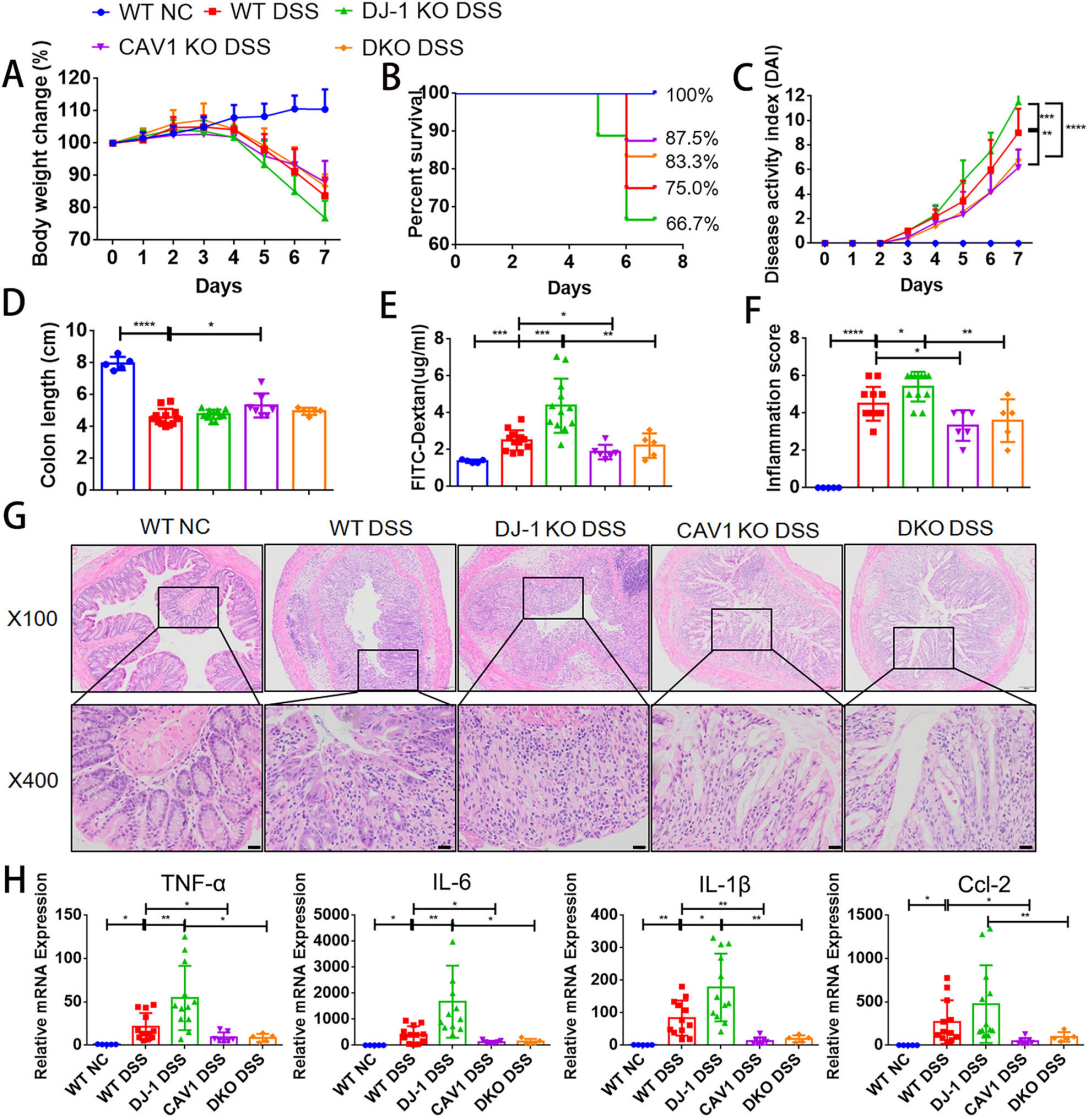
CAV1 knockout rescued DSS-induced colitis in DJ-1deficient mice. WT, DJ-1 KO, CAV1 KO and DKO mice were treated with DSS for 7 days. WT NC = 5, WT DSS = 12, DJ-1 KO DSS = 12, CAV1 KO DSS = 6, DKO DSS = 5. Body weight change (**A**), survival rates (**B**) and the disease activity index (**C**) were monitored daily. **D** Mouse colon lengths were measured after sacrifice. **E** Intestinal permeability was evaluated by measuring the concentration of FITC-dextran in the blood serum. Histological scores (**F**) were determined in a double-blinded manner, and the histological analysis of colon tissue samples is shown (**G**) (upper: magnification ×100; scale bar = 100 µm, lower: magnification ×400; scale bar = 20 µm). **H** Quantitative PCR analysis was used to assess cytokine and chemokine production in whole-colon homogenates. DAI scores and inflammation scores are expressed as median and IQR. Other All data are the means ± SD. *p < 0.05, **p < 0.01, ***p < 0.001, ****p < 0.0001, ns no significant, two-tailed.

Based on the above results, we generated CAV1 and DJ-1 double knockout (DKO) mice to verify whether CAV1 plays a role in IBD pathogenesis downstream of DJ-1 (Supplementary Fig. S5A). WT, DJ-1 KO, CAV1 KO and DKO mice were treated with DSS for 7 days. Body weight loss, survival rate and DAI scores were significantly ameliorated in the DKO mice compared with the DJ-1 KO mice (Fig. 5A–C). Although there is no difference in colon length shortening between DKO mice and DJ-1 KO mice after DSS molding, fluorescein isothiocyanate-conjugated dextran (FITC-dextran, Sigma, USA) analysis indicated that intestinal permeability was markedly decreased compared to that in the DJ-1 KO DSS mice (Fig. 5D, E). Histological examination corresponded with the expression levels of proinflammatory cytokines, which were elevated in the DJ-1 KO mice but significantly reduced in the CAV1 and DKO mice (Fig. 5F–H).

### The DJ-1/CAV1 pathway regulated epithelial inflammation through necroptosis in colitis

**Fig. 6:**
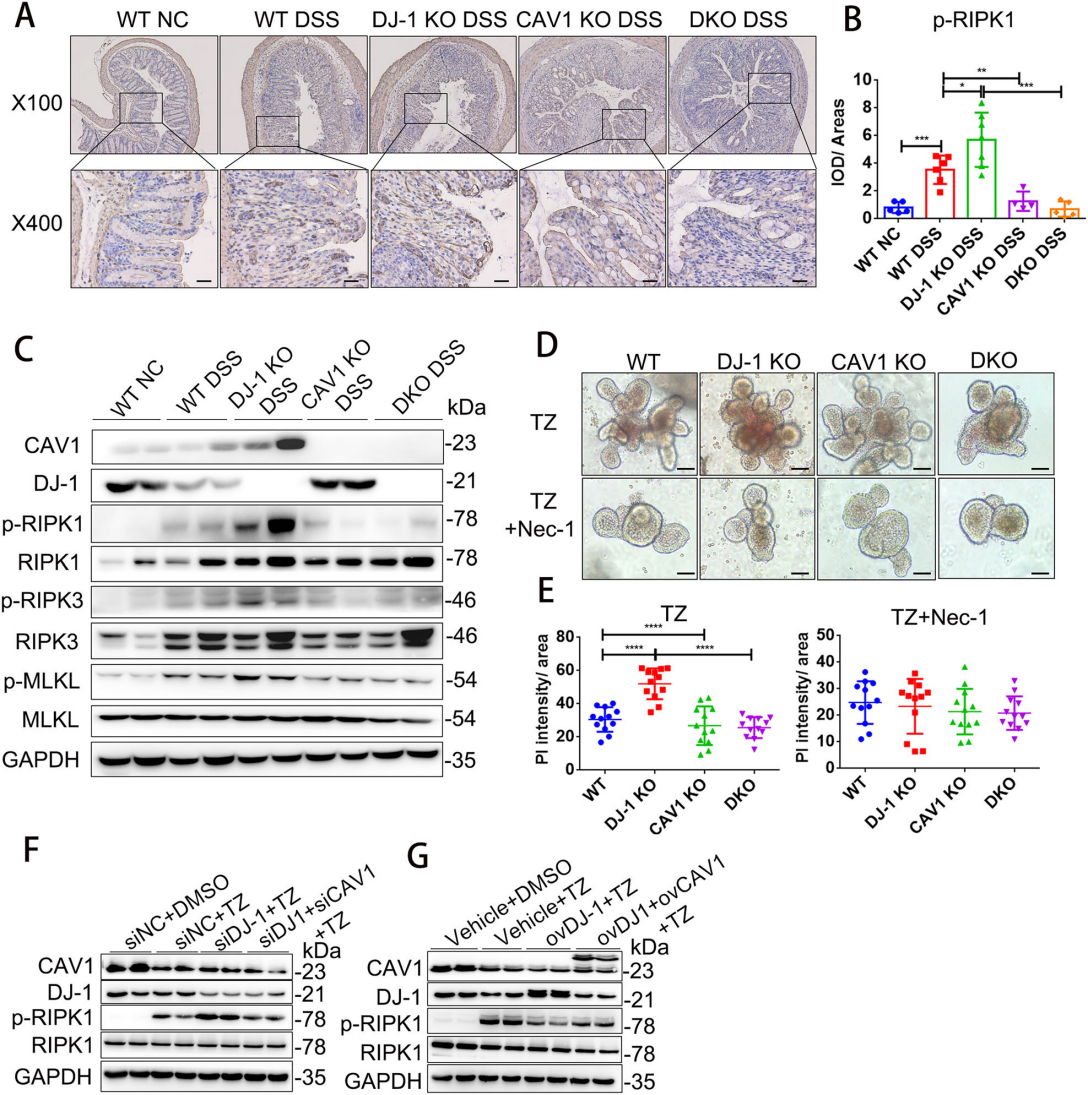
CAV1 promoted epithelial necroptosis under the regulation of DJ-1. **A** Representative IHC analysis of p-RIPK1 expression in colonic tissue samples (IHC upper: magnification ×100；scale bar = 100 µm, lower: magnification ×400；scale bar = 20 µm). **B** Quantification of IHC analysis of p-RIPK1 (WT NC = 5, WT DSS = 6, DJ-1 KO DSS = 7, CAV1 KO DSS = 4, DKO DSS = 5) expression. **C** Western blotting was used to analyze CAV1, DJ-1 and necroptosis signaling pathway molecule protein levels in colon tissue samples from the mice treated as described above. **D** Intestinal organoids from the WT, DJ-1 KO, CAV1 KO and DKO mice treated as indicated with the combination of TNF-α (100 ng/ml) and zVAD-fmk (25 µM) (TZ), or TZ+ necrostatin-1 (Nec-1, 30 µM) for 12 h and stained with PI (red) for 24 h (magnification ×200；scale bar = 50 µm). **E** Quantification of PI intensities. *n* = 12 organoids from 3 mice per group. **F** DJ-1 and CAV1 expression were knocked down in HCT116 cells, and the cells were stimulated with TZ for 24 h. **G** Western blot analysis of DJ-1 and CAV1 overexpression in the TZ-stimulated HCT116 cells. All data are the means ± SD. *p < 0.05, **p < 0.01, ***p < 0.001, ****p < 0.0001, two-tailed.

**Fig. 7:**
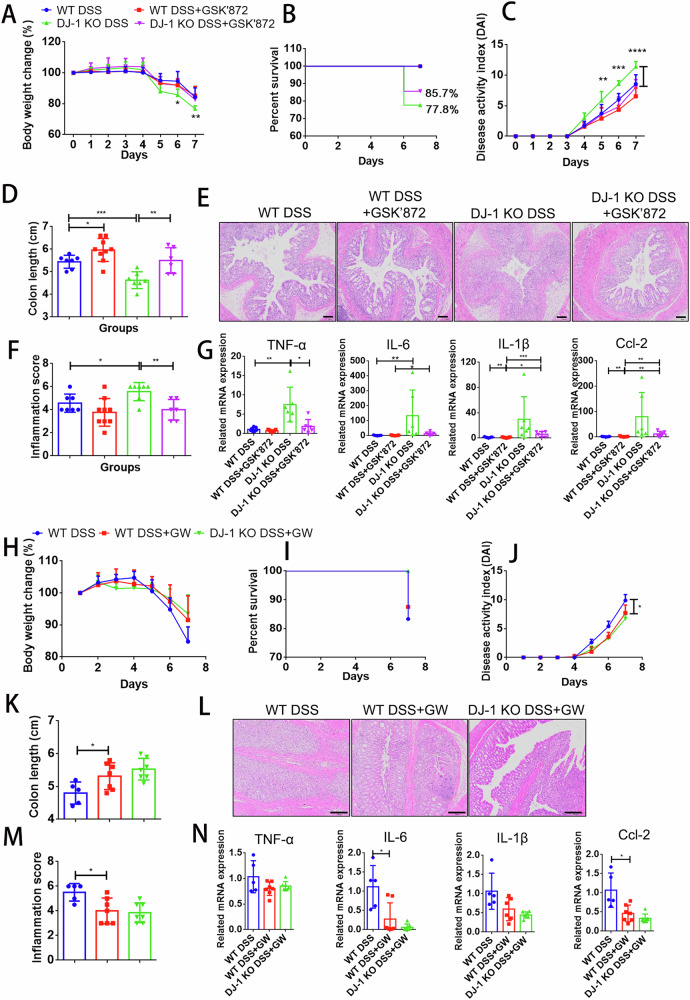
Pharmacologic inhibition of necroptosis relieved DJ-1 deficiency-induced experimental colitis. WT and DJ-1 KO mice were treated with 2 mg/ml GSK’872 every two days by intraperitoneal injection after 7 days of DSS administration. WT DSS = 7, WT DSS + GSK’872 = 9, DJ-1 KO DSS = 7, DJ-1 KO DSS + GSK’872 = 6. Body weight change (**A**), survival rates (**B**) and the DAI scores (**C**) were scored daily. Mice were sacrificed on Day 7, and colon lengths (**D**) were measured. **E** HE staining in colon tissue samples from the WT and DJ-1 KO mice is shown (magnification ×100; scale bar=100 µm). **F** Semiquantitative histopathological scoring was performed. **G** Quantitative PCR analysis of cytokines and chemokines in colons from the WT and DJ-1 KO DSS-treated mice. The WT and DJ-1 KO mice were treated with 1 mg/ml GW806742X (GW) for each day by intraperitoneal injection under a 7-day DSS administration. WT DSS = 5; WT DSS + GW = 7; DJ-1 KO + GW = 7. Body weight change (**H**), survival rates (**I**) and DAI scores (**J**) were scored daily. Colon lengths (**K**) were measured. H&E staining (**L**) in colon tissue samples is shown, and semiquantitative scoring of histopathology (**M**) was performed (upper: magnification ×40; scale bar = 500 µm, lower: magnification ×100; scale bar=200 µm). **N** Quantitative PCR analysis of cytokines and chemokines in colons. DAI scores and inflammation scores are expressed as median and IQR. Other All data are the means ± SD. *p < 0.05, **p < 0.01, ***p < 0.001, ****p < 0.0001, two-tailed.

Furthermore, we found that necroptosis was downstream of DJ-1/CAV1 in DKO mice with colitis and organoids. Histological staining of p-RIPK1 in colonic sections confirmed that necroptosis occurred significantly more in the DJ-1 KO mice, while CAV1 knockout rescued these effects. (Fig. 6A, B). In addition, in DSS-induced colitis, the DJ-1 deficiency-induced activation of p-RIPK1, p-RIPK3 and p-MLKL was dramatically blunted in the DKO mice (Fig. 7C, S6A). We then induced necroptosis in organoids from the intestines of mice of different genotypes [27]. The organoids were basically mature on day 5 (Supplementary Fig. S6B). When the organoids were stimulated with a combination of zVAD-fmk and recombinant mouse TNF-α, propidium iodide (PI) intensity revealed that DJ-1-deficient organoids exhibited increased sensitivity to necroptosis induction, while CAV1 deficiency or double deficiency protected the organoids from necroptosis. And when the organoids treated with Nec-1s, a chemical inhibitor of necroptosis, the differences in PI intensity in organoids of different genotypes disappeared (Fig. 6D, E). In addition, in vitro, we also found that rescued cell inflammation was observed after knockdown of CAV1 compared with DJ-1 knockdown alone (Supplementary Fig. S6C). And in gain-of-function experiments, we observed that DJ-1 overexpression decreased the expression of proinflammatory cytokines, while CAV1 overexpression aggravated cell inflammation (Supplementary Fig. S6D). Moreover, knockdown of CAV1 in the DJ-1-deficient necroptosis model rescued the expression of p-RIPK1 compared with DJ-1 knockdown alone (Fig. 6F). And DJ-1 overexpression decreased the expression of p-RIPK1, while CAV1 overexpression again aggravated cell necroptosis (Fig. 6G). Based on the above evidence, we concluded that DJ-1/CAV1 pathway regulated epithelial necroptosis in colitis.

### Pharmacological inhibition of necroptosis relieved DJ-1 deficiency and DSS-induced experimental colitis

GSK’872, a chemical inhibitor of RIPK3 kinase activity [28], was evaluated in DJ-1-deficient colitis. The results showed that the body weight loss, survival rate, DAI scores and colon length shortening were exacerbated by DJ-1 deficiency compared to those in the WT DSS-treated mice, whereas these adverse effects were dramatically blunted in the DJ-1 KO mice intraperitoneally injected with GSK’872 (Fig. 7A–D, Supplementary Fig. S7A). Moreover, histological examination results indicated that GSK’872 significantly suppressed colitis symptoms and led to the maintenance of gut barrier integrity in the mice (Fig. 7E, F). Consistently, the inflammatory response was reduced by GSK’872 in the DJ-1 KO DSS-treated mice (Fig. 7G).

Moreover, we applied GW806742X [29], an MLKL inhibitor that binds the MLKL pseudokinase domain, to the WT and DJ-1 KO DSS-treated mice. After the administration of GW806742X to the WT and DJ-1 KO mice, the exacerbation of colitis due to DJ-1 deficiency was relieved, as shown by the body weight loss, survival rate, DAI scores, colon length, histological examination and inflammatory cytokines (Fig. 7H–N, Supplementary Fig. S7B). And GW806742X showed the ability to decrease the protein levels of MLKL in the colon of the DSS-treated mice (Supplementary Fig. S7C), indicating an inhibitory effect on necroptosis. These results suggested that DJ-1 protection against colitis was largely dependent on RIPK3/MLKL-dependent necroptosis signaling.

Taken together, our research demonstrated the key role of CAV1 in colitis and revealed the mechanism by which CAV1 promoteed epithelial necroptosis, leading to impairment of the epithelial barrier. This process was negatively regulated by DJ-1-mediated protein degradation. These data provided novel insights into key aspects of IBD pathogenesis with broad therapeutic implications.

## Discussion

The present study reveals the detrimental involvement of CAV1 in epithelial necroptosis. Analysis of both GEO databases and colonic tissues from individuals with IBD indicated a noteworthy increase in CAV1 accumulation in IBD subjects. Notably, CAV1 knockout mitigated DSS-induced intestinal inflammation. The accumulation of epithelial CAV1 was found to contribute to the disruption of the epithelial barrier and inflammation. Additionally, our findings highlighted that DJ-1, a protective protein against epithelial colitis, facilitatesd the degradation of CAV1, thereby inhibiting the CAV1/necroptosis axis in the pathogenesis of IBD. This study is the first to identify DJ-1 as a mediator of CAV1, suggesting that the pivotal role played by CAV1 in triggering necroptosis ultimately results in epithelial inflammation in colitis.

CAV1 is a key protein in the cell membrane and invaginations involved in inflammation [5]. Kevil et al. showed that genetic deletion or pharmacologic inhibition of CAV1 significantly decreased colitis histopathology scores and reduced vascular densities [6]. However, other studies have shown that CAV1 protects against DSS-induced colitis [8]. Given these different conclusions, we believed that the underlying function and mechanism of CAV1 in IBD are important. Our long-term study of CAV1 found that it is complex and flexible in IBD, and the mechanism by which CAV1 modulates inflammation differs in different populations of colon cells, such as fibroblasts, endothelial cells and epithelial cells [6, 30]. In stenotic CD, CAV1 deficiency induced fibroblast activation and inhibited CD-induced intestinal fibrosis [30]. However, in active colitis, CAV1 was a harmful factor in IBD. In our study, we focused on epithelial cell death and inflammation, of which CAV1 was capable, rather than endothelial-mediated inflammation, as reported before. Both the transcript and protein levels indicated increased CAV1 expression in IBD patients. Genetic deletion and overexpression of CAV1 also showed that CAV1 was a harmful factor in maintaining epithelial homeostasis against foreign stimuli. Furthermore, CAV1 KO bone marrow chimeric DSS mice did not show a state of remission, indicating that immune cells did not participate in the pathogenesis of CAV1-mediated colitis. These findings first suggested that the downregulation of CAV1 expression protected against inflammation in IBD.

To mechanistically verify the contribution of epithelial CAV1 to colitis, we examined the relationship between CAV1 and DJ-1. DJ-1 is a protein that was first reported to be associated with parkinsonism [31], and it is an essential antiapoptotic regulator [32]. Our previous study revealed that a defect in DJ-1 expression might aggravate inflammation and epithelial cell apoptosis through p53 signaling [11]. However, the underlying mechanism remains unknown. It was reported that DJ-1 modulated CAV1 expression by regulating the stability of CAV1 via the proteasomal degradation pathway [33, 34]. In our research, we revealed a marked interaction between DJ-1 and CAV1, and we proved that DJ-1 promoted CAV1 protein degradation to inhibit inflammation. Our study was not the first to propose that the degradation of CAV1 protein is regulated by other molecules. It is reported that TRAF4 specifically interacted with the N-terminal tail of CAV1, and regulated CAV1 stability by preventing ZNRF1-mediated ubiquitination and facilitating USP7-mediated deubiquitination independently of its E3 ubiquitin ligase catalytic activity [35]. Thus, it is possible that TRAF4 acts as a key upstream regulator of CAV1 expression in colitis; however, definitive confirmation necessitates additional experimental evidence. Therefore, regulation of the protein levels of CAV1 might be an important part of IBD pathogenesis-related epithelial inflammation.

In addition to apoptosis, necroptosis is another pathway that regulates epithelial inflammation and is speculated to be downstream of the DJ-1/CAV1 pathway. Our early study reported that DJ-1 deficiency exacerbated apoptosis, and in this research, we found that DJ-1 deficiency exacerbated necroptosis as well, identifying an unreported pathway of DJ-1 in IBD. The necroptosis signaling pathway is modulated by the kinase RIPK1 and relies on the kinases RIPK3 and MLKL [36]. The mechanisms underlying the necroptosis pathway mostly involve the binding of TNF to TNFR1, and TNFR1 trimers lead to the recruitment of RIPK1 [15, 36, 37]. TNF stimulation and caspase 8 deficiency promoted the formation of a cytosolic necrosome [26, 38]. RIPK3 and MLKL deficiency was reported to inhibit DSS-induced colitis [39, 40], and MLKL or the RIPK3/necroptosis axis is a driving force for intestinal inflammation. In our study, we found that CAV1 accumulation sensitized the cells to TZ treatment, indicating that CAV1 promoted necroptosis in epithelial cells. Moreover, in vivo, we found that inhibition of RIPK3 or MLKL pharmacologically completely rescued DJ-1 deficiency-related colitis. Consistent with the organoid experiment, a genetic defect of CAV1 could rescue the epithelial necroptosis caused by DJ-1 deficiency, indicating that the DJ-1/CAV1/necroptosis pathway participated in the pathogenesis of colitis. However, the connection between CAV1 and necroptosis is not fully elucidated.

To verify the clinical value of CAV1 and necroptosis in colitis, we collected intestinal samples from IBD patients and observed that CAV1 and p-MLKL were both significantly increased in advanced UC and CD patients compared with healthy controls. In addition, it has been reported that increased phosphorylation of the necroptotic pathway was positively correlated with enhanced TNF-α in IBD patients [28]. Thus, the clinical application of necroptosis inhibitors may have a therapeutic effect on the progression of colitis. In addition, targeting DJ-1 expression or CAV1 degradation can contribute to new drug development in IBD treatment.

However, there are still some limitations in our study. A notable limitation of this study is the suboptimal transduction efficiency of CAV1 overexpression via tail vein injection of AAV7. To address this, future research should prioritize optimizing delivery methods, such as employing mesenteric artery injection [41], which could enhance tissue-specific targeting and expression efficiency despite its technical challenges. Additionally, exploring alternative viral vectors or adjunct therapies to amplify CAV1 expression warrants further investigation to improve therapeutic outcomes. In the bone marrow suppression experiments, we could only demonstrate that immune cells play no role in CAV1-mediated inflammation in colitis. However, we lack conclusive data to specify which non-hematopoietic cell population mediates the functional effects of CAV1. To further elucidate that CAV1 primarily exerts its effects through intestinal epithelium, we propose generating epithelial-specific conditional knockout models to precisely evaluate CAV1 epithelial functions, or employing single-cell RNA sequencing to comprehensively identify pathogenic non-hematopoietic cell populations for future investigations. In vitro experiments, we found that the expression of CAV1 in HT29 and HCT116 cells slightly decreased after TZ modeling. The different expression results in animal and cell models are considered negative feedback results, and the differences are interesting and worthy of further exploration. Besides, the use of colorectal cancer cell lines, though practical for initial mechanistic exploration, may not fully replicate the physiological complexity of normal colonic epithelium. Although Mouse intestinal organoids were included to better model in vivo epithelial dynamics, interspecies differences between murine and human systems could affect translational interpretation. Further work incorporating complementary human primary or near-physiological models, such as HCEC-1CT, will help bridge these gaps. Mechanistically, we assumed that DJ-1 might degrade CAV1 through proteasomal degradation, since we observed an increase in the ubiquitination level of CAV1 protein in the human intestinal epithelial cell line HCT116 following DJ-1 overexpression. However, the specific types of ubiquitin chain linkages and the precise ubiquitination site on the CAV1 substrate require further elucidation. As reported, we hypothesize that DJ-1 may functionally collaborate with ZNRF1 in regulating CAV1 stability [42], while this hypothesis requires rigorous experimental validation. In addition, we cannot explain how CAV1 modulates downstream necroptosis. It was reported that RIPK3 phosphorylated MLKL that oligomerizes and translocates to the plasma membrane to execute necroptosis or induce mitochondrial fission in necroptosis [43, 44]. The existence of a potential connection to the previous report remains uncertain, necessitating further investigation into additional mechanisms. In addition, there is another assumption about the CAV1/necroptosis pathway in which the plasma membrane protein CAV1 regulates ESCRT-III complex formation to promote MLKL-dependent calcium influx and phosphatidylserine exposure on the outer leaflet of the plasma membrane in the necroptotic process [45]. Hence, further studies are required to elucidate the possible mechanisms involved in the DJ-1/CAV1/necroptosis pathway in colitis.

Overall, our findings establish for the first time that DJ-1 promotes CAV1 degradation to inhibit the disruption of the epithelial barrier and protect against necroptosis in colitis. These results offer conceptually novel insights into the pathogenesis of CAV1-induced inflammation and provide a potential therapeutic approach for colitis treatment.

## Methods

### Human samples

Intestinal tissue samples were obtained from healthy controls (*n* = 14), UC patients (*n* = 10) and CD patients (*n* = 12). The characteristics of the patients and healthy subjects are summarized in Supplementary Table S1. A more detailed description of human subjects was set out in the supplementary material.

### Animals

Male C57BL/6 mice were purchased from the Nanjing Biomedical Research Institution of Nanjing University (Nanjing, China), and CAV1 KO mice and DJ-1 KO mice were described in our previous studies [11, 21]. Along with the newly bred DKO mice, all the transgenic mice were identified with PCR analysis of genomic DNA, and the primers are listed in Supplementary Table S2. The dextran sulfate sodium (DSS, MP Biomedicals, USA)-induced UC model was established with 7 days of 3.5% DSS administration. For the gain-of-function experiment, 8-week-old male C57BL/6 mice were intravenous injected with RFP-tagged CAV1-overexpressing AAV7 (vector: Pav-CMV-P2A-RFP-3Flag) or control AAV7 (Vigene Biosciences), and then, the mice were subjected to DSS after 3 weeks. For the CAV1 KO bone marrow chimera experiment, the bone marrows of 6–8 weeks old donor C57BL/6 (wild type, WT) mice or CAV1 KO mice were flushed using 1640 medium (Gibco, USA). Red Blood Cell Lysis Buffer (Dawen Biology, China) was added to disintegrate the erythrocytes, washed again with PBS and filtered through a 70 µm cell strainer (Biosharp, China). The cells were resuspended in PBS and stored on ice after cell counting. 8-week-old male C57BL/6 mice received a dose of γ-ray irradiation (7.5 Gy) and were then injected intravenously with the isolated bone marrow cells at 1 ×10^7^ cells per mouse immediately. Three weeks after bone marrow reconstitution, the genotypes were evaluated, and the mice were subjected to DSS after another 3 weeks. For the necroptosis inhibition experiments, WT and DJ-1 KO mice were treated with 2 mg/mL GSK’872 (MCE, USA) every two days or 1 mg/mL GW806742X (MCE, USA) each day by intraperitoneal injection under a 7-day DSS administration. All mice were housed in Zhejiang Academy of Medical Sciences in a standard environment.

Sample size specifications have been systematically integrated into the corresponding methodological sections of the manuscript. Mice were randomized into experimental cohorts using a dynamic allocation strategy, wherein the total number of groups was determined by both the logistical demands of post-intervention sample collection and the empirical trends observed in preliminary survival analyses. To maintain methodological transparency, investigators remained unblinded to group assignments during both the experimental phase and outcome evaluation processes.

### Organoid culture

Mouse intestinal organoids were cultured using IntestiCult organoid growth medium according to the manufacturer’s instructions (Stemcell Technologies, Canada) [46]. In brief, the colons of WT, DJ-1 KO, CAV1 KO and DKO mice were separated and washed repeatedly 15–20 times in cold (2–8 °C) PBS. After the tissue was cut into 2 mm segments, the fragments were resuspended in 25 mL of Gentle Cell Dissociation Reagent (Stemcell Technologies) at room temperature (15–25 °C) and shaken at room temperature at 20 rpm for 15 min. Dissociated tissues were rotated at 290 g for 5 min at 2–8 °C, followed by resuspension in PBS with 0.1% BSA (Sigma, USA). Then, the crypts were filtered through 70 μm strainers and plated in 24-well culture plates (Corning) in IntestiCul Organoid Growth Medium mixed 1:1 with prechilled mixed Matrigel Matrix (Corning). IntestiCult™ Organoid Growth Medium was added to the cell culture plates to immerse the matrix. The medium was changed every 2 days. After 5–7 days, 100 ng/ml TNF-α (PeproTech, China) and/or 25 µM zVAD-fmk (MCE, USA) were added to the medium for 24 h, and 30 µM necrostatin-1 (Nec-1, MCE, USA) was added for 12 h. Then, the organoids were stained with propidium iodide (PI, Sigma, USA) for 24 h before dead cell imaging was performed [27, 47].

### Cell lines

The HEK293 cell and colorectal carcinoma cell lines HCT116 and HT-29 were purchased from the Chinese Academy of Sciences (Shanghai, China) and cultured in Dulbecco’s modified Eagle’s medium with 10% fetal calf serum (Gibco, USA) and 100 U/ml penicillin‒streptomycin (Sigma-Aldrich, USA). HCT116 cells were treated for 24 h with 100 ng/ml TNF-α (PeproTech, China) to establish a cell model of colitis. For establishment of a necroptosis model, HCT116 and HT-29 cells were stimulated with 100 ng/ml TNF-α and 25 µM Z-VAD-fmk (MCE, USA) for 24 h. HCT116 and HT-29 cells were transfected with a CAV1-specific siRNA (siCAV1, 5’-CCACCTTCACTGTGACGAA-3’), a DJ-1-specific siRNA (siDJ-1, 5’-GAUUAAGGUCACCGUUGCA-3’) (Ruibobio, Guangzhou, China) or the corresponding negative control for 24 h using Lipofectamine 3000 (Invitrogen). Flag-tagged CAV1 and HA-tagged DJ-1 overexpression plasmids were transfected into HCT116 and/or HT-29 cells using Lipofectamine 3000 according to the manufacturer’s instructions for 24 h before further TNF-α and/or 25 µM Z-VAD-fmk treatment. The LDH level (Dojindo, Kyushu, Japan) in culture supernatants were measured according to the manufacturer’s instructions to assess death cells in vitro. HEK293 cells infected with FLAG-tagged CAV1, HA-tagged DJ-1 plasmid and empty vector were stimulated with TNF-α and Z-VAD-fmk for 24 h and then treated with 25 µM MG132 (MCE, USA) or 3-MA (10 mM, MCE) for another 6 h before being harvested.

### Bioinformatics analysis

The expression levels of CAV1 in intestinal tissues from IBD patients and healthy controls were obtained from the RNAseq datasets GSE16879, and GSE59071 in the GEO database (www.ncbi.nlm.nih.gov/gds).

### Statistical analysis

The statistical analyses were conducted using GraphPad Prism 8 software. All data are presented as means ± SD or median and interquartile range (IQR) and represent a minimum of three independent experiments. Statistical significance was determined using appropriate methods such as two-tailed Student’s *t* test, one-way ANOVA, or non-parametric test. Results with a *P*-value less than 0.05 were considered statistically significant. Absence of a *p*-value for a comparison explicitly indicated that no statistically significant difference was observed between those groups.

Other materials and methods are described in details in the supplementary materials.

## Supplementary information

Revised Supplementary materials

Original western blots

## Data Availability

The authors declare that all other data supporting the findings of this study are available within the article and its Supplementary material files, or are available from the authors upon request.
