# Supplementary material for: DJ-1 counteracts Caveolin-1-mediated necroptosis to inhibit epithelial barrier dysfunction in colitis
Source: Cell Death Dis. 2025 Aug 29;16(1):657. doi: 10.1038/s41419-025-07989-z (PMC12394565; doi:10.1038/s41419-025-07989-z)
Supplement: Supplementary file 1 — Revised Supplementary materials [file 41419_2025_7989_MOESM1_ESM.docx]

**DJ-1 counteracts Caveolin-1-mediated necroptosis to inhibit epithelial barrier dysfunction in colitis**

**Supplementary figures**

**Supplementary tables**

**Supplementary materials and methods**

**Supplementary figures**

**
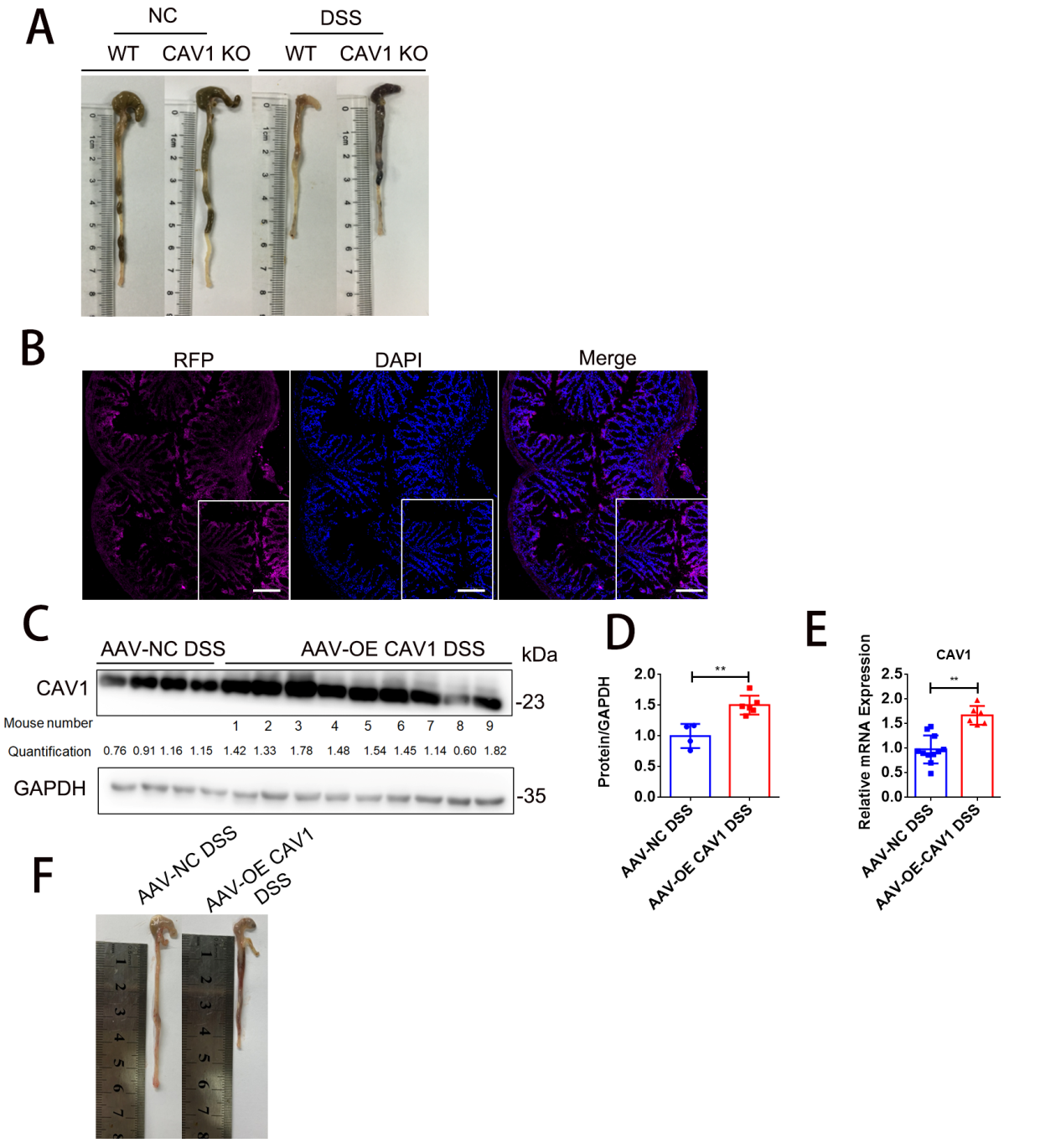
**

**Supplementary Figure 1 CAV1 promoted DSS-induced experimental colitis.**

(A) Mice were treated with 3.5% DSS for 7 days. Colon appearances were [photograph](javascript:;)ed. (B) Representative immunofluorescence staining picture of the RFP-tagged CAV1-overexpressing AAV7 infectious colons (magnification ×400，scale bar=150 µm). (C) Western blotting and gray quantitative analysis were used to analyze CAV1 protein levels in colon tissue samples. Based on the quantitative results of protein grayscale analysis of CAV1 in the colonic tissue of mice, specimens from mice numbered 1-6 were selected for further analysis. (D) Semiquantitative analysis of CAV1 protein. (E) Quantitative PCR analysis of CAV1 was performed. AAV-NC DSS=11; AAV-OE CAV1 DSS=6. (F) Representative colon images were captured from AAV-OE-CAV1 DSS-treated mice and compared with those from AAV-NC DSS-treated mice. The data are shown as the means±SDs. **p<0.01, two-tailed.


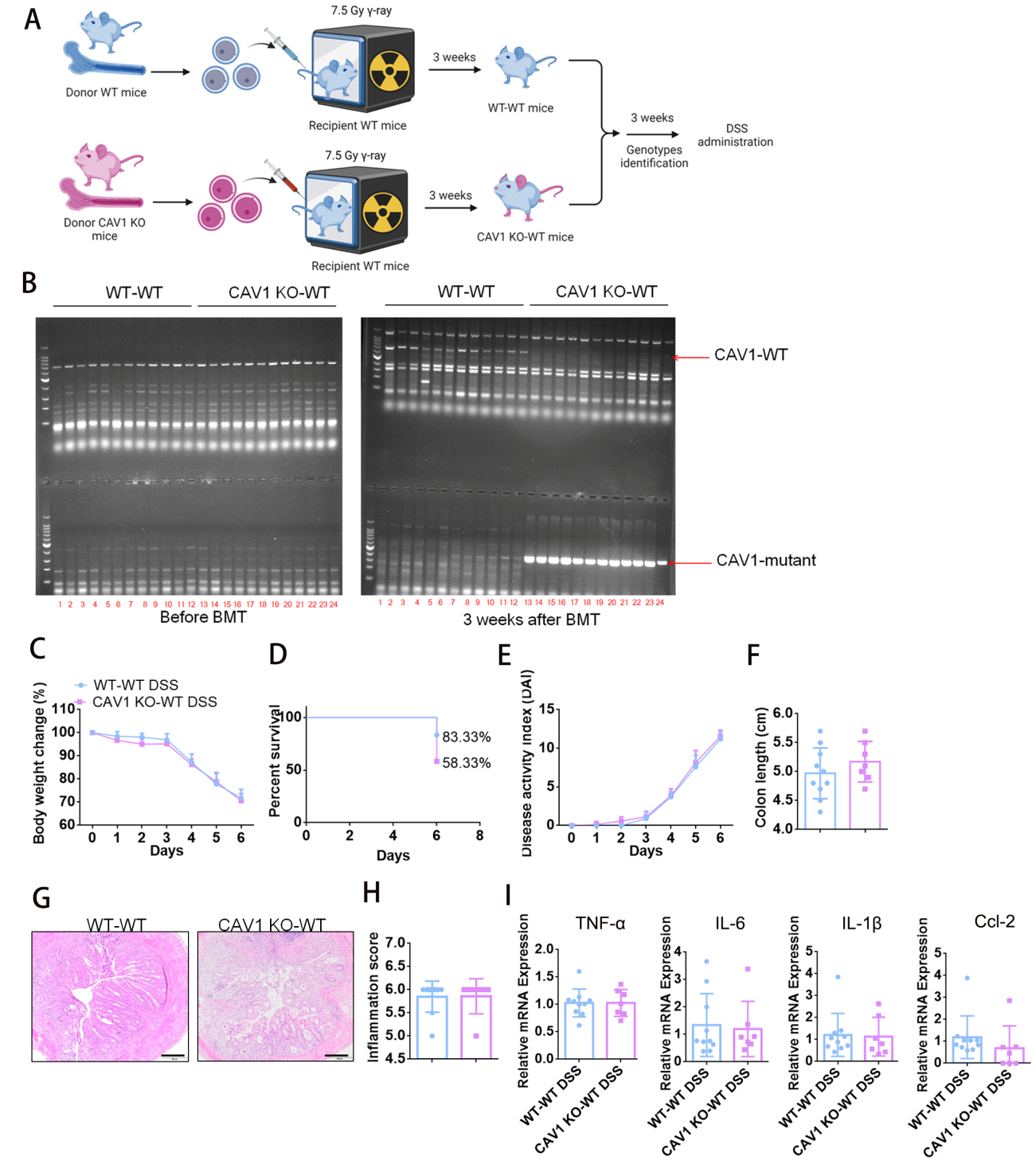


**Supplementary Figure 2 The genomic DNA of the bone marrow transplantation experiment and the efficiency verification of CAV1 knockdown or overexpression in vitro.**

(A) Diagram of the bone marrow chimera experiment: WT recipient mice received a dose of γ-ray irradiation (7.5 Gy) and were then injected intravenously with bone marrow cells from donor WT mice or CAV1 KO mice. Six weeks after bone marrow reconstitution, the mice were subjected to DSS. WT to WT (WT-WT)=10, CAV1 KO to WT (CAV1 KO-WT)=7. (B) PCR analysis of genomic DNA. The left picture is the genomic DNA of experimental mice before bone marrow transplantation (BMT), and the right picture is that of mice after bone marrow reconstitution. Body weight change (C), survival rates (D), DAI scores (E) and colon length (F) were measured. (G) Representative H&E staining images were [photograph](javascript:;)ed (magnification ×100; scale bar=200 µm). (H) Semiquantitative scoring of histopathology was performed, and (I) quantitative PCR analysis of inflammatory markers was performed. DAI scores and inflammation scores are expressed as median and IQR. Other all data are the means±SD.


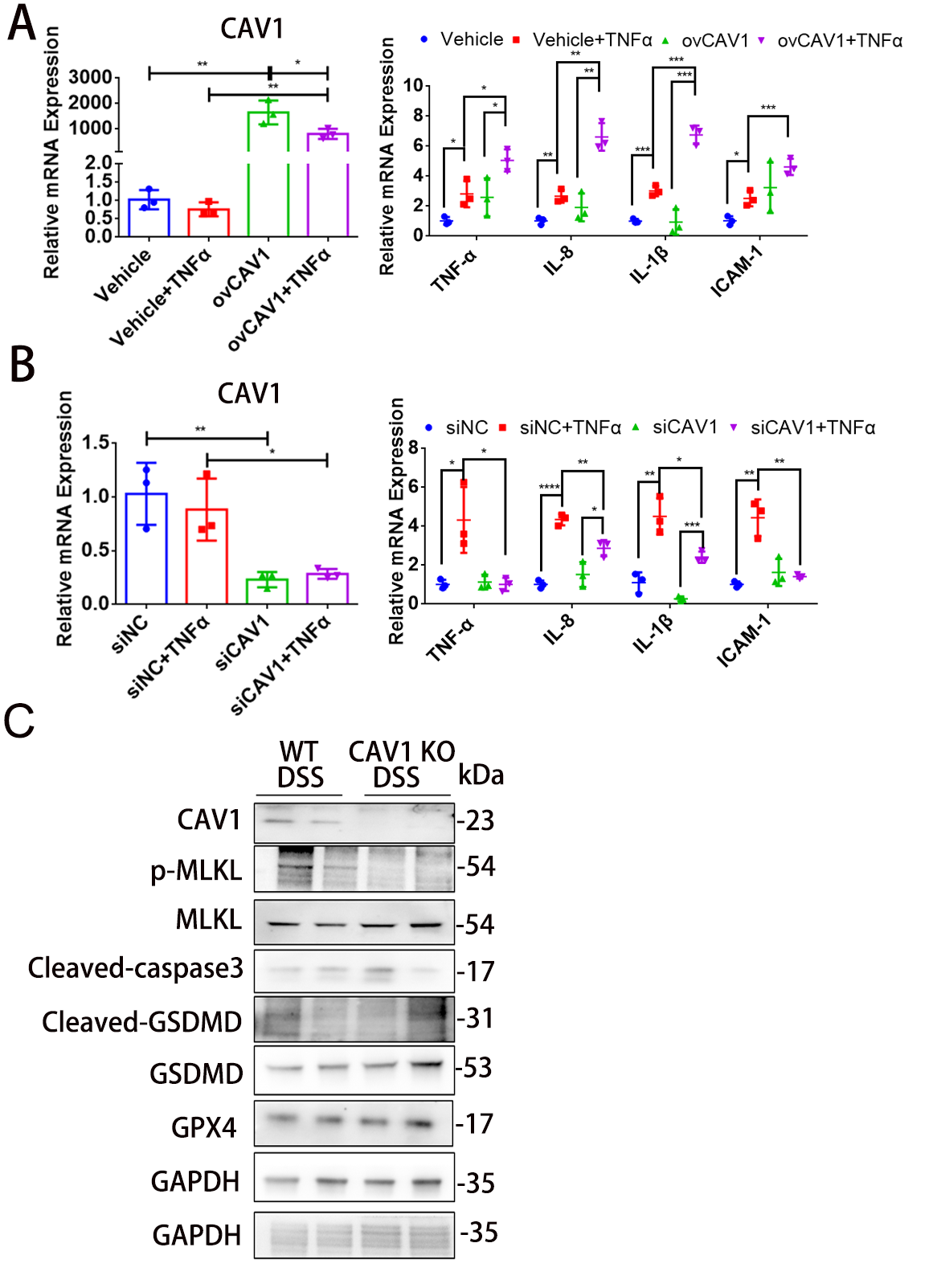


**Supplementary Figure 3 Epithelial CAV1 accumulation contributed to intestinal inflammation.**

(A) CAV1 overexpression was induced by transfecting cells with a Flag-tagged CAV1 plasmid for 24 h and stimulating cells with 100 ng/ml TNF-α. Inflammatory cytokines, such as TNF-α, IL-8, IL-1β and ICAM-1, were quantified by using quantitative PCR (n=3).(B) CAV1 expression was knocked down in HCT116 cells by siRNAs for 24 h, and then, the cells were stimulated with 100 ng/ml of TNF-α for another 24 h. Proinflammatory cytokines were quantified using quantitative PCR (n=3). (C) Western blot analysis was performed to assess the protein expression of cell death markers in DSS-treated WT and CAV1 KO mice. All data are the means±SD. *p<0.05, **p<0.01, ***p<0.001, ****p<0.0001, two-tailed.


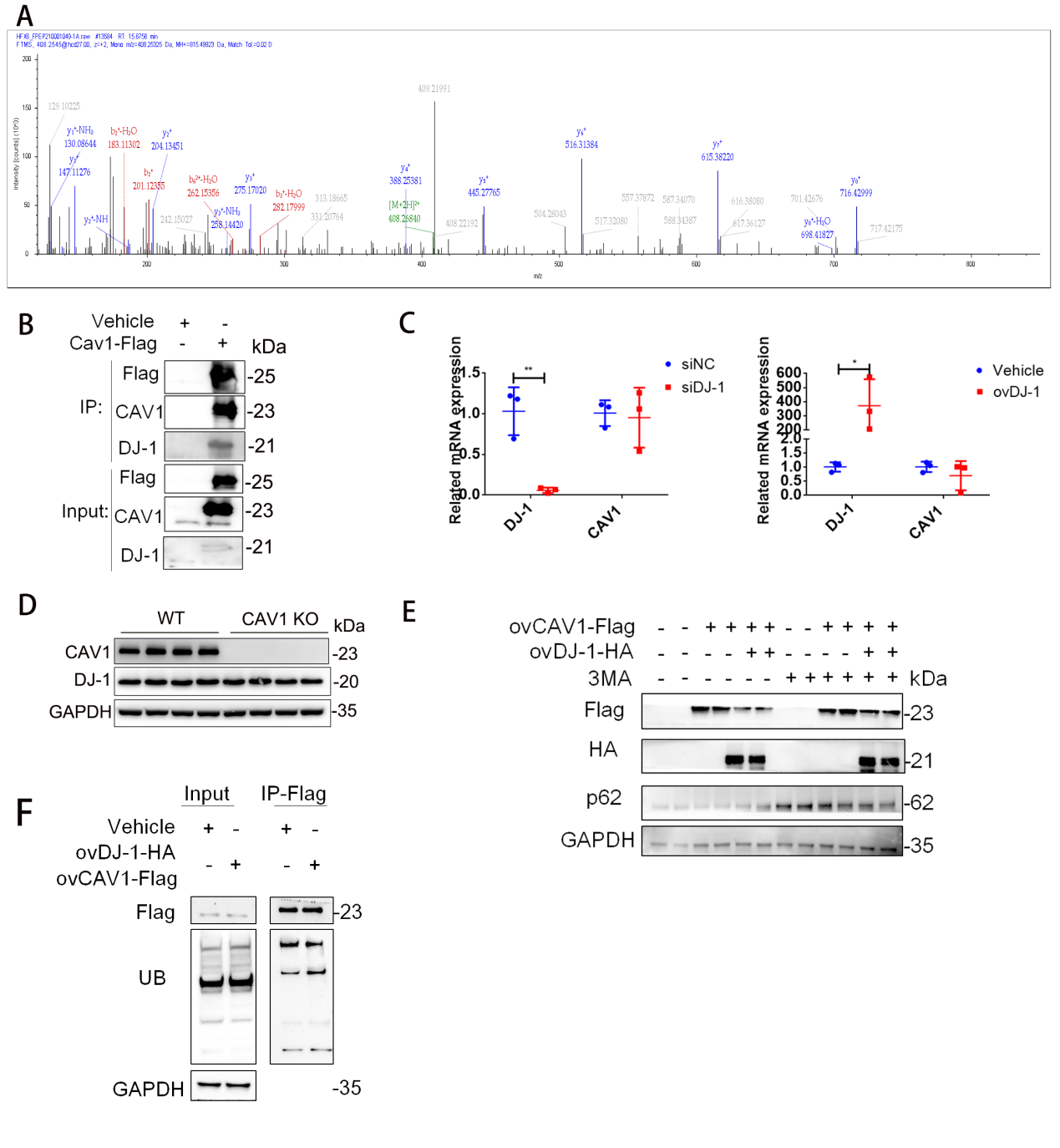


**Supplementary Figure 4 The relationship between CAV1 and DJ-1.**

（A）The CAV1-FLAG plasmid was overexpressed in HEK293 cells, and the cell lysates were immunoprecipitated by FLAG beads. Protein profile sequencing showed that the pulled down proteins contained DJ-1-specific protein peptides. (B) HEK293 cells were infected with a FLAG-tagged CAV1 overexpression plasmid or the empty vector (vehicle), and total FLAG-tagged CAV1 was immunoprecipitated. (C) Quantitative PCR analysis was used to assess the mRNA expression of DJ-1 and CAV1 in HCT116 cells after DJ-1 knockdown or overexpression (n=3). (D) The expression of the colonic CAV1 and DJ-1 proteins in the WT and CAV1 KO mice (n=4). (E) HEK293 cells were infected with FLAG-tagged CAV1, HA-tagged DJ-1 plasmid and empty vector were stimulated with 100 ng/ml TNF-α and 25 µM zVAD-fmk for 24 h and then treated with 3-MA (10 mM，MCE) for another 6 h. (F) HCT116 cells were infected with FLAG-tagged CAV1, HA-tagged DJ-1 plasmid and empty vector, and total FLAG-tagged CAV1 was immunoprecipitated. All data are the means±SD. *p<0.05, **p<0.01, two-tailed.


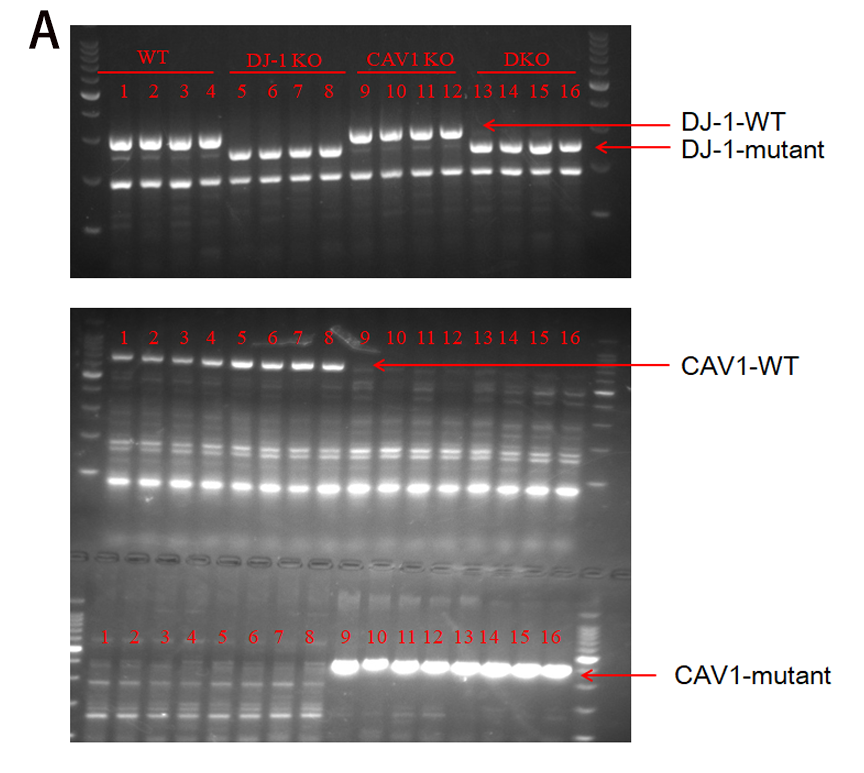


**Supplementary Figure 5 PCR analysis of genomic DNA.**

(A) From top to bottom are the DJ-1 wild-type allele, DJ-1 mutant allele, CAV1 wild-type allele, and CAV1 mutant allele (WT mice: 1-4, DJ-1 KO mice: 5-8; CAV1 KO mice: 9-12, DKO mice: 13-16)

**
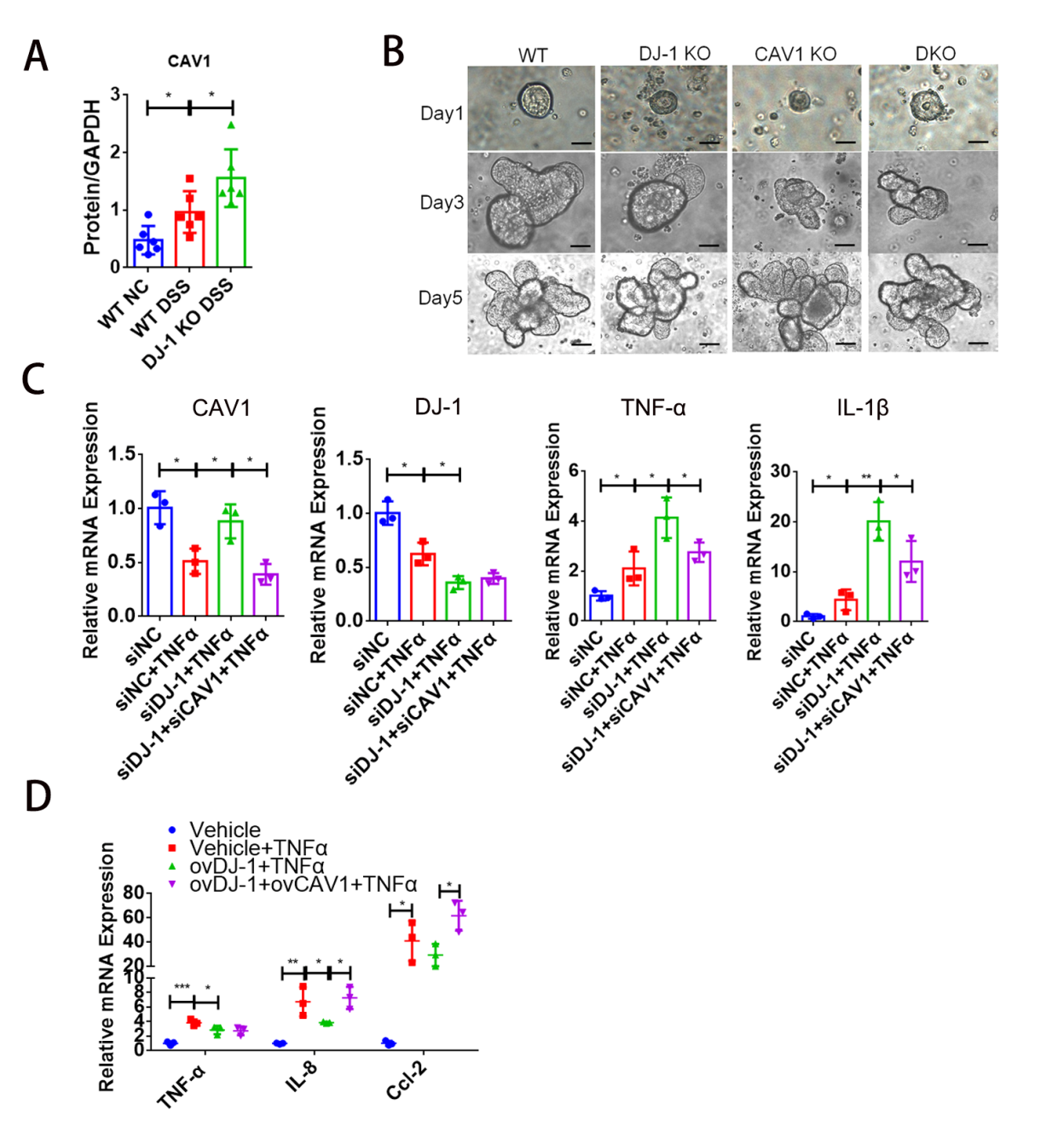
**

**Supplementary Figure 6 The DJ-1/CAV1 pathway regulated epithelial necroptosis in vitro.**

(A) Quantitative analysis of the CAV1 protein levels (n=6). (B) Images of cultured organoids from mice of different genotypes were collected every two days (magnification ×200；scale bar=50 µm).（C）DJ-1 and CAV1 were knocked down in HCT116 cells by siRNAs for 24 h, and the cells were stimulated with 100 ng/ml of TNF-α for 24 h. Proinflammatory cytokines were quantified using quantitative PCR (n=3). (D) Quantitative PCR analysis of TNF-α, IL-8, and CCL2 in the TNF-α-stimulated HCT116 cells after overexpression of CAV1 and DJ-1 for 24 h (n=3). All data are means±SD. *p<0.05, **p<0.01, ***p<0.001, two-tailed.

**
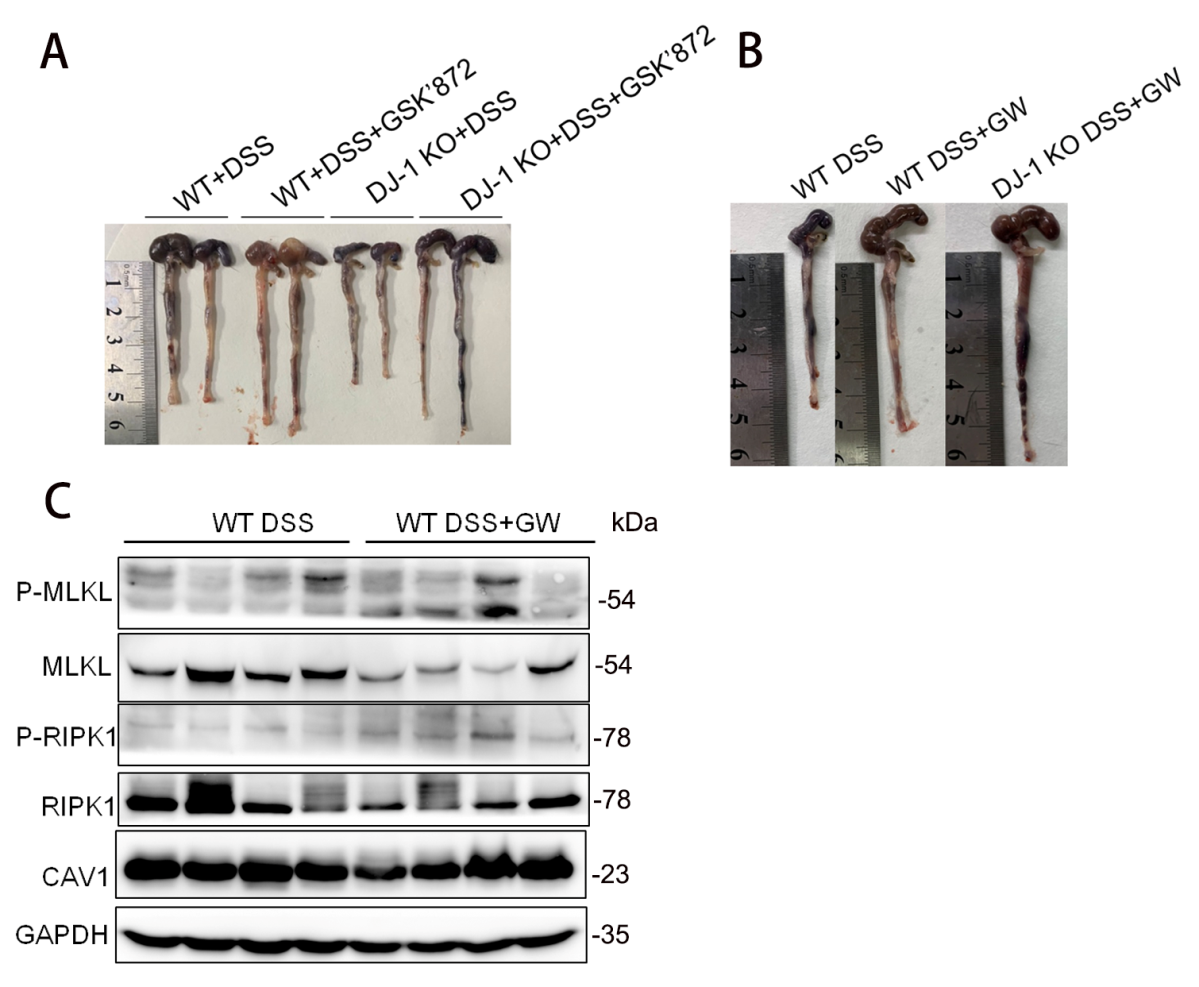
**

**Supplementary Figure** 7 **Pharmacologic inhibition of necroptosis relieved DJ-1-deficient DSS-induced experimental colitis.**

(A) The colon appearances of the GSK’872-treated WT and DJ-1 KO mice. (B) The colon appearances of the GW806742X (GW)-treated WT and DJ-1 KO mice. (C) Western blot analysis of necroptosis-related proteins in the colon of the GW-treated mice.

**Supplementary tables**

**Supplementary table 1**

Clinical Characteristics of IBD Patients and Healthy controls.

|  | Healthy controls | Active UC | Active CD |
| --- | --- | --- | --- |
| Number of patients | 14 | 10 | 12 |
| Age (y) | 50.57 ± 4.59 | 47.80 ± 5.88 | 37.25 ± 3.90 |
| Gender |  |  |  |
| Male | 8 | 5 | 8 |
| Female | 6 | 5 | 4 |
| Current therapy |  |  |  |
| 5-aminosalicylates |  | 10 | 12 |
| Immunosuppressants |  | 0 | 3 |
| Biologics |  | 0 | 0 |
| Nutritional therapy |  | 0 | 0 |
| Corticosteroids therapy |  | 0 | 0 |
| Probiotic therapy |  | 2 | 0 |
| Disease extent (UC)^a^ |  |  |  |
| E1: ulcerative proctitis |  | 2 |  |
| E2: left-sided UC |  | 3 |  |
| E3: extencive UC |  | 5 |  |
| Disease extent (CD)^a^ |  |  |  |
| L1: distal ileum |  |  | 3 |
| L2: colon |  |  | 2 |
| L3: ileum and colon |  |  | 5 |
| L4: [upper](javascript:;) [gastrointestinal](javascript:;) [tract](javascript:;) |  |  | 2 |

^a^ According to the Montreal classification system.

**Supplementary table 2**

**Primers for mice identification**

| Primer name | Sequence 5’-3’ |
| --- | --- |
| CAV1-Wild type F | GTGTATGACGCGCACACCAAG |
| CAV1-Mutant F | CTAGTGAGACGTGCTACTTCC |
| CAV1-Common R | CTTGAGTTCTGTTAGCCCAG |
| DJ1-oIMR7006 | GCTGAAACTCTGCCATGTGA |
| DJ1-oIMR7007 | ACTTGGAGTCCCCTCGTTTT |
| DJ1-oIMR8162 | TGGATGTGGAATGTGTGCGAG |

**Primers for Quantitative PCR dectection in mice (**[**1**](#_ENREF_1)**,** [**2**](#_ENREF_2)**)**

| Primer name | Sequence 5’-3’ |
| --- | --- |
| m-CAV1-F | ATGTCTGGGGGCAAATACGTG |
| m-CAV1-R | CGCGTCATACACTTGCTTCT |
| m-GAPDH-F | TGGCCTTCCGTGTTCCTAC |
| m-GAPDH-R | GAGTTGCTGTTGAAGTCGCA |
| m-PARK7(DJ-1)-F | AGCCGGGATCAAAGTCACTG |
| m-PARK7(DJ-1)-R | GGTCCCTGCGTTTTTGCATC |
| m-IL-6-F | TAGTCCTTCCTACCCCAATTTCC |
| m-IL-6-R | TTGGTCCTTAGCCACTCCTTC |
| m-TNF-α-F | CAGGCGGTGCCTATGTCTC |
| m-TNF-α-R | CGATCACCCCGAAGTTCAGTAG |
| m-IL-1β-F | TTCAGGCAGGCAGTATCACTC |
| m-IL-1β-R | GAAGGTCCACGGGAAAGACAC |
| m- Ccl-2(MCP1)- F | GAGGACAGATGTGGTGGGTTT |
| m- Ccl-2(MCP1)-R | AGGAGTCAACTCAGCTTTCTCTT |

**Primers for Quantitative PCR dectection in human (**[**1**](#_ENREF_1)**,** [**2**](#_ENREF_2)**)**

| Primer name | Sequence 5’-3’ |
| --- | --- |
| h-CAV1-F | GCGACCCTAAACACCTCAAC |
| h-CAV1-R | ATGCCGTCAAAACTGTGTGTC |
| h-GAPDH-F | GGAGCGAGATCCCTCCAAAAT |
| h-GAPDH-R | GGCTGTTGTCATACTTCTCATGG |
| h- PARK7(DJ-1)-F | AACCGGAAGGGCCTGATAG |
| h- PARK7(DJ-1)-R | GCAAGAGGGTGTGTTGTAACT |
| h- TNF-α-F | GAGGCCAAGCCCTGGTATG |
| h- TNF-α-R | CGGGCCGATTGATCTCAGC |
| h- IL-8-F | ACTGAGAGTGATTGAGAGTGGAC |
| h- IL-8-R | AACCCTCTGCACCCAGTTTTC |
| h- IL-1β-F | AGCTACGAATCTCCGACCAC |
| h- IL-1β-R | CGTTATCCCATGTGTCGAAGAA |
| h- Ccl-2-F | GAGGACAGATGTGGTGGGTTT |
| h- Ccl-2-R | AGGAGTCAACTCAGCTTTCTCTT |

Abbreviations: m-, mouse; h-, human

**Supplementary materials and methods**

**Human subjects**

To investigate the difference in expression of the target proteins in the intestine of healthy controls and IBD patients, we recruited a subset of healthy controls who underwent colonoscopy and patients who were highly suspicious of IBD based on their clinical presentation. We informed them of the details of our study and sought their consent and signed written informed consent. We pre-selected 20 clinical specimens for each group and collected intestinal epithelial tissue endoscopically. Patients who met the pathological diagnosis of UC (n=10) and CD (n=12) were re-selected to be included in the IBD group, and other healthy patients without colon polyps or intestinal inflammation were re-selected to be in the healthy control group (n=14). Relevant laboratory tests were then performed on these clinical specimens. The risk in this clinical study is extremely low, mainly from delayed bleeding after colonoscopy biopsy. To prevent the risk of alteration from occurring, subjects will be followed up for 3 days post-procedure to look for adverse effects such as black stools, bloody stools and abdominal pain. Although there is no direct benefit to the patient from this study, it is hoped that the findings gained from this study will be of use in guiding the clinical management of other IBD patients in the future. Patients will be compensated for free treatment in the event of serious adverse events, such as delayed bleeding, during participation in this clinical study. To protect patient privacy, records will be kept by case number and no personal information such as patient names will be released to the public.

**DNA extraction and genetic background identification**

The DNA of hair follicles was extracted from toe tissues of WT, DJ-1 KO, CAV1 KO, and DKO mice. Briefly, the toes were placed in a 1.5 mL EP tube and mixed with 180 µL of a 50 mmol/L sodium hydroxide solution. The mixture was then vortexed and heated at 95°C for 10 minutes. After adding 20 µL of tris-HCl (pH=8.0), centrifugation was performed at 12000 rpm for 5 minutes to obtain the supernatant for subsequent PCR amplification. Additionally, the DNA of WT-WT mice and CAV1 KO-WT mice was extracted from peripheral bloods three weeks after bone marrow transplantation using orbital veins as collection sites. DNA extraction followed the manufacturer's instructions (Simgen, Hangzhou, China). To determine the genetic background, transgenic mice were identified through PCR analysis on genomic DNA using primers listed in Table S2. Amplified DNA samples were obtained using Green Taq Mix according to the manufacturer's protocol (Vazyme, Nanjin, China). A gel electrophoresis system consisting of agarose gel supplemented with Gelred stain in a TAE solution was prepared as a medium for separating DAN bands by loading both ladder and PCR-amplified DNA samples into sample wells on the agarose gel. The separated bands were observed under UV light and photographed.

**Immunoprecipitation**

HEK293 cells and HCT116 cells were transfected with the Flag-tagged CAV1 and Flag-tagged DJ-1 plasmids. Subsequently, the cells were treated with lysis buffer (Pierce) and then immunoprecipitated with anti-Flag beads (Sigma, USA) for 3 h (7.5 circles/min). For endogenous protein IP, HEK293 cells and HT-29 cells lysates were immunoprecipitated using an anti–DJ-1 antibody (Abcam, 76008) and anti-IgG plus protein agarose (Thermo Fisher Scientific). After the immunoprecipitates were washed, the immunoprecipitated proteins were incubated with loading buffer and horizontally vibrated. The final supernatants were analyzed using western blotting .

**Determination of DAI scores and histological scores**

The DAI scores and histological scores were performed as described previously ([1](#_ENREF_1)). Briefly, for DAI scores, the maximum total sum of the DAI score is 12, each mouse was calculated daily according to the following criteria: (1) Stool consistency: 0, well-formed stool, 2, pasty and semi-formed stool that does not adhere to the anus; 4, liquid stool that adheres to the anus. (2) Fecal blood: 0, negative occult blood test (Beckman Coulter); 2, positive occult blood test; 4, gross bleeding. (3) Body weight loss from baseline: 0, no weight loss; 1, 1%-5% weight loss; 2, 6%-10% weight loss; 3, 11%-20% weight loss; 4, >20% weight loss. For inflammation histological scores, inflammatory cell infiltration was scored as 0–3, and tissue damage was scored as 0–3.

**Intestinal permeability to fluorescein**

The model mice's intestinal permeability was evaluated using FITC-dextran (Sigma, USA), which is a fluorescently labeled dextran compound. Following a 6-hour fasting period, the mice were orally administered with a solution containing FITC-dextran at a dosage of 600 mg/kg. After another 6 hours, the mice were sacrificed and their plasma was separated through centrifugation. The diluted plasma in PBS was then analyzed using an excitation wavelength of 480 nm and an emission wavelength of 520 nm.

**Immunohistochemistry and immunofluorescence staining**

The immunohistochemistry and immunofluorescence staining were described in detail in our previous study ([3](#_ENREF_3)). For immunohistochemistry, the sections were incubation with primary antibodies CAV1 (CST, 3267, 1:150), DJ-1(Abcam, 18257, 1:150), ZO-1(Abcam, 96587, 1:200), p-RIPK1 (CST, 38662, 1:200), cleaved-caspase3 (CST, 9664, 1:500), p-MLKL (Abcam, 187091, 1:200) at 4°C overnight. For immunofluorescence staining, the primary antibodies of CAV1 and DJ-1(Abcam, 11251, 1:100) were used, and sections were incubated with DyLight 488-conjugated goat anti-mouse IgG or DyLight 594-conjugated goat anti-rabbit IgG (Abbkine) and then stained with DAPI (ZSGB Bio, China). Three fields at least were randomly selected for each sample, and the IOD of the positive staining in was measured with ImagePro Plus 6.0 software.

**TUNEL staining**

The intestinal tissues sections were detected by TUNEL staining according to the manufacturer’s instructions (Roche, Mannheim, Germany).

**Western blotting analysis**

Protein extraction from cells or intestinal samples was performed as previously described ([1](#_ENREF_1)). Primary and secondary antibodies were used as follows: antibodies anti-CAV1 (3267, 1:1000), anti-HA tag (5017, 1:2000), anti-GAPDH (5176, 1:5000), anti-Ubiquitin (3936, 1:2000), anti-p-RIPK1 (38662 for mouse, 1:500; 44590 for human, 1:500), anti-RIPK1 (3493, 1:1000), anti-p-MLKL(37333 for mouse, 1:500; 91689 for human, 1:500), anti-MLKL(37705 for mouse, 1:1000; 14993 for human, 1:1000), anti-p-RIPK3 (91702 for mouse, 1:500; 93654 for human, 1:500), anti-RIPK3 (15828 for mouse, 1:1000; 13526 for human, 1:1000), anti-Cleaved-caspase3 (9664, 1:1000), anti-Cleaved-GSDMD (10137, 1:1000), anti-GSDMD (39754, 1:1000) were obtained from Cell Signaling Technology. Antibodies anti-DJ-1(18257, 1:1000) was obtained from Abcam. Anti-Flag tag (20543-1-AP, 1:5000) and anti-GPX4 (67763-1-lg, 1:2000) were purchased from Proteintech. Anti-p62/SQSTM1 (PM045) was obtained from MBL International. And the corresponding HRP-conjugated secondary antibody (Sigma) at a 1:5,000 dilution was applied followed. Proteins were detected with an ECL light detection kit (Lianke Multi Sciences, China). GAPDH was used as the control.

**RNA isolation and real-time quantitative PCR**

The total RNA was extracted using TRIzol Reagent (manufactured by TaKaRa, Otsu, Japan) following the manufacturer's instructions. PrimeScript RT master mix (manufactured by TaKaRa, Otsu, Japan) was used for cDNA synthesis. For quantitative real-time PCR analysis, Evo M-MLV RT Premix for qPCR (provided by Accurate Biotechnology, China) and a Bio-Rad CFX384 system were utilized. Real-time PCR was performed using SYBR Green Premix Pro Taq HS qPCR Kit (supplied by Accurate Biotechnology, China) and an Applied Biosystem 7500 instrument. GAPDH served as the reference gene in ∆∆Ct method to determine relative expression levels. Please refer to Table S2 for primer sequences.

**REFERENCES**

1. Zhang J, Xu M, Zhou W, Li D, Zhang H, Chen Y, et al. Deficiency in the anti-apoptotic protein DJ-1 promotes intestinal epithelial cell apoptosis and aggravates inflammatory bowel disease via p53. The Journal of biological chemistry. 2020;295(13):4237-51.

2. Li C, Chen Y, Zhu H, Zhang X, Han L, Zhao Z, et al. Inhibition of Histone Deacetylation by MS-275 Alleviates Colitis by Activating the Vitamin D Receptor. Journal of Crohn's & colitis. 2020;14(8):1103-18.

3. Yu M, Wu H, Wang J, Chen X, Pan J, Liu P, et al. Vitamin D receptor inhibits EMT via regulation of the epithelial mitochondrial function in intestinal fibrosis. The Journal of biological chemistry. 2021;296:100531.
