# Supplementary material for: DJ-1 counteracts Caveolin-1-mediated necroptosis to inhibit epithelial barrier dysfunction in colitis
Source: Cell Death Dis. 2025 Aug 29;16(1):657. doi: 10.1038/s41419-025-07989-z (PMC12394565; doi:10.1038/s41419-025-07989-z)
Supplement: Supplementary file 2 — Original western blots [file 41419_2025_7989_MOESM2_ESM.docx]

**DJ-1 counteracts Caveolin-1-mediated necroptosis to inhibit epithelial barrier dysfunction in colitis**

Figure 1E


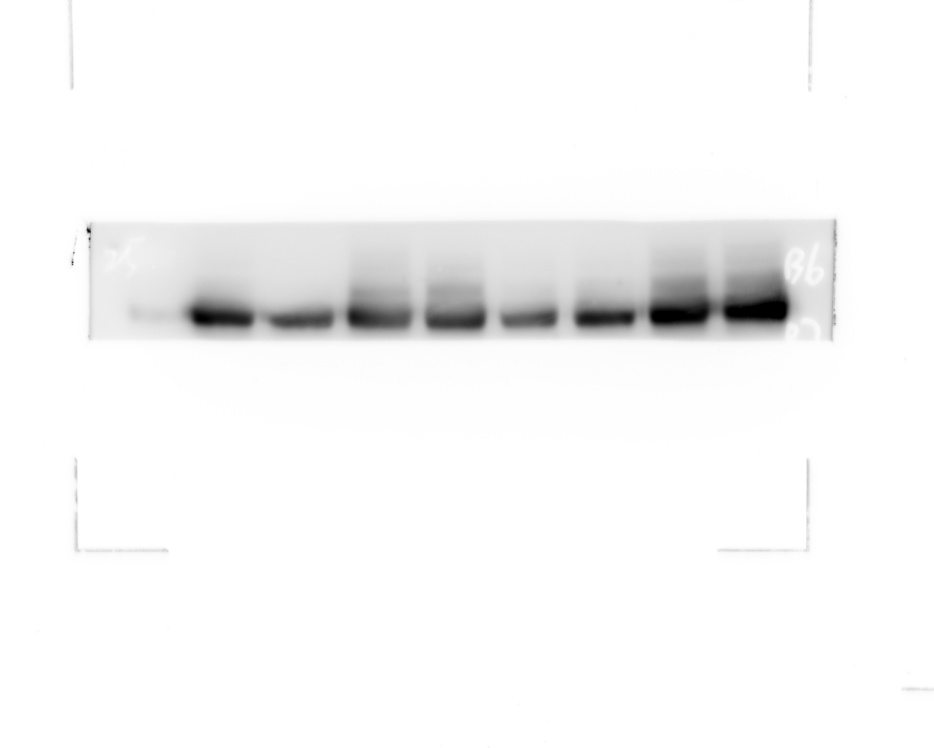


ZO-1 -220 kDa

GAPDH -35kDa


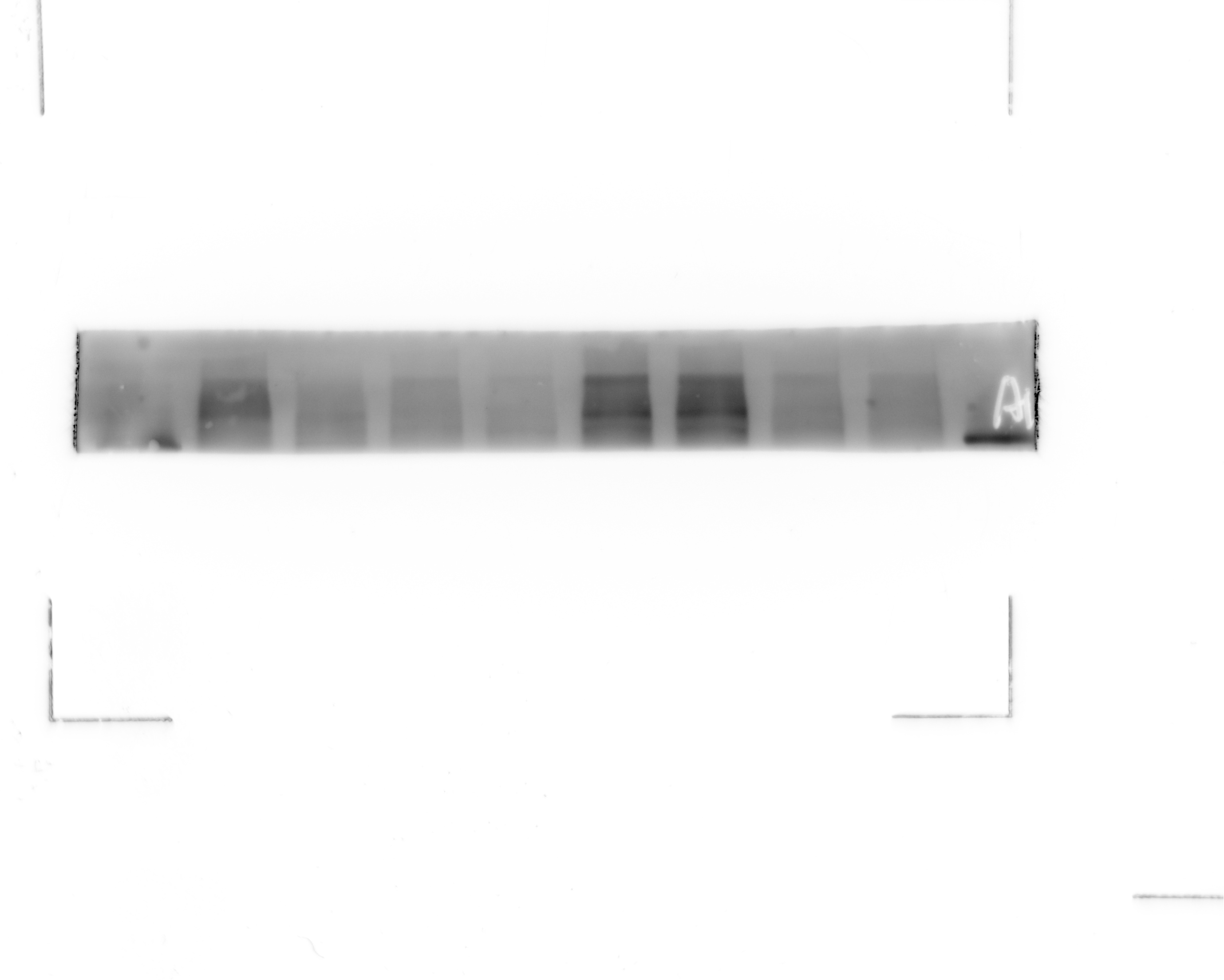

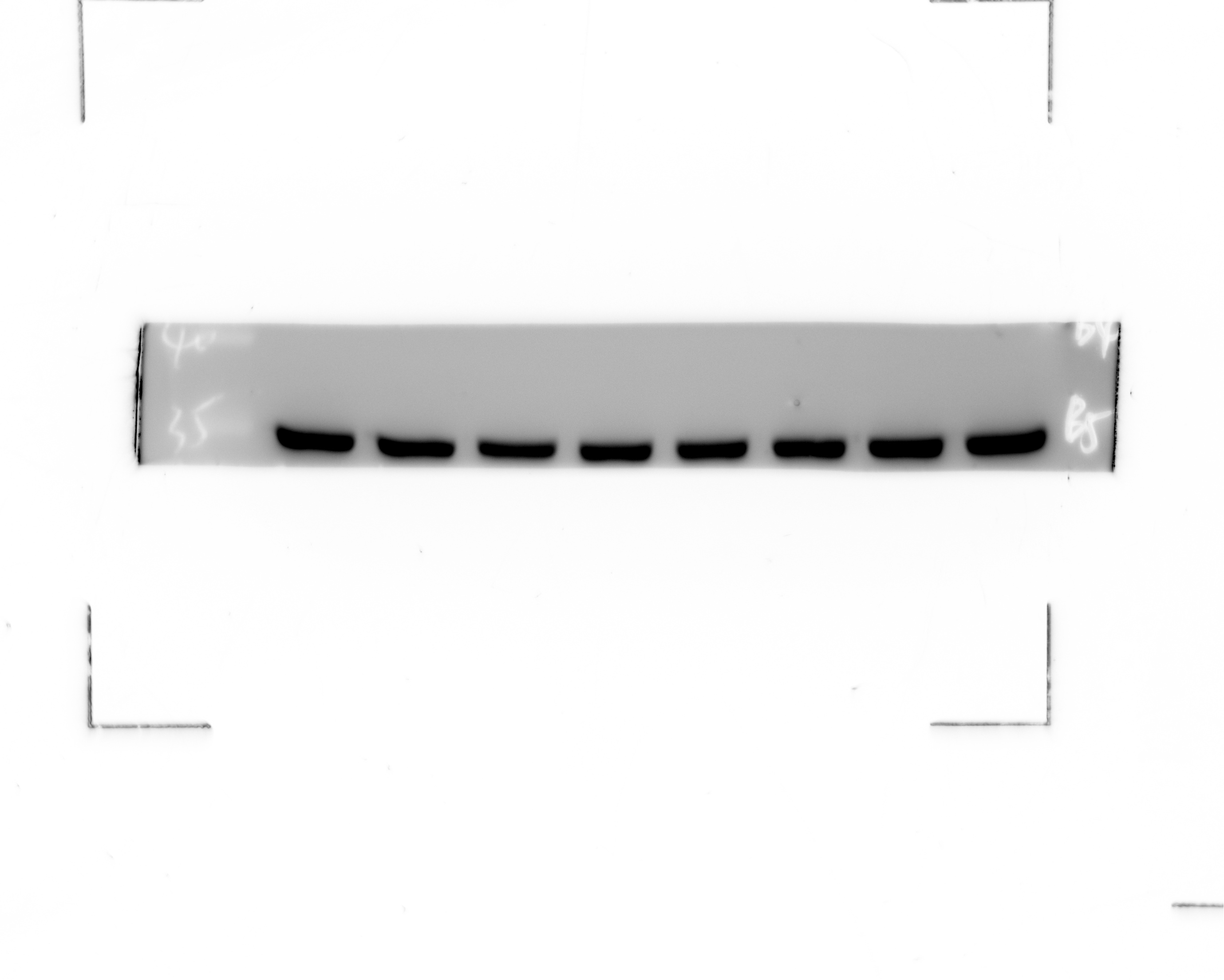


CAV1 -23kDa

Figure 1F


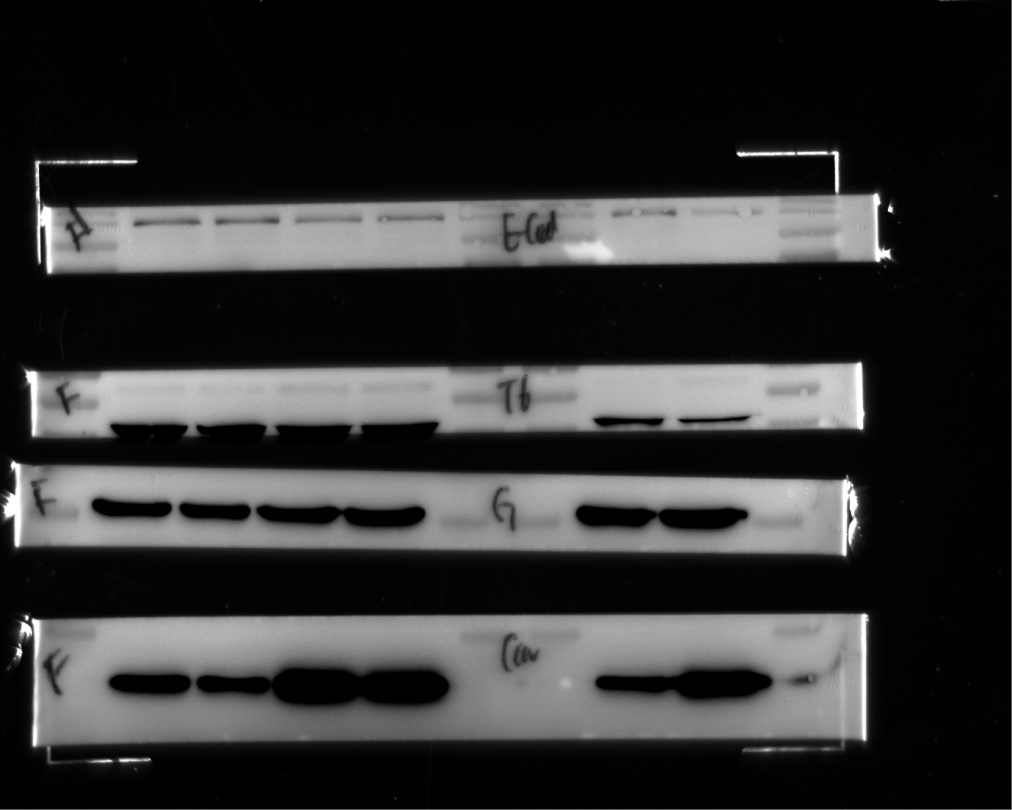


CAV1 -23kDa

GAPDH -35kDa

Figure 3H


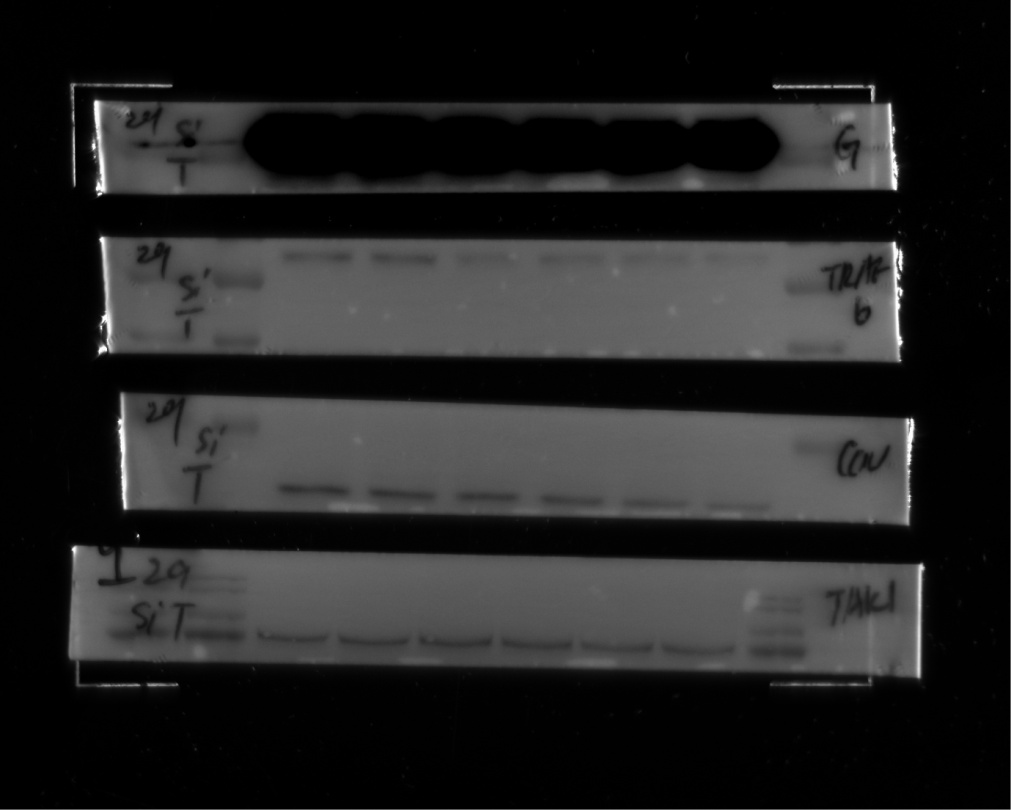


CAV1 -23kDa


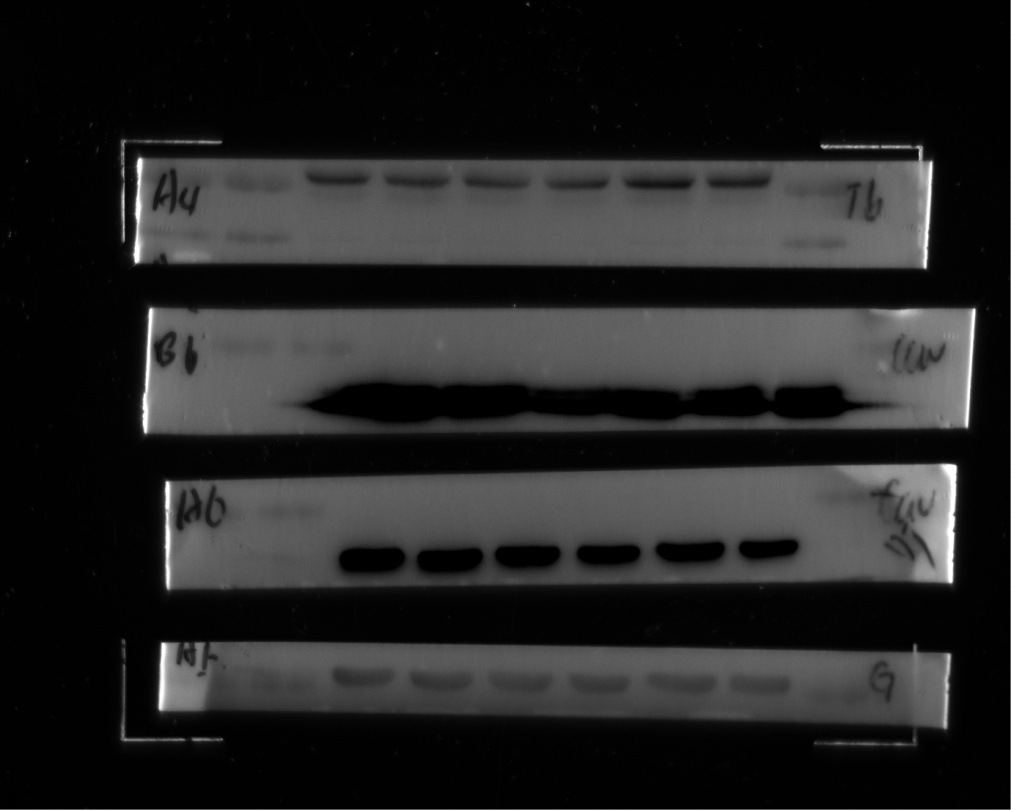


GAPDH -35kDa

DJ-1 -21kDa


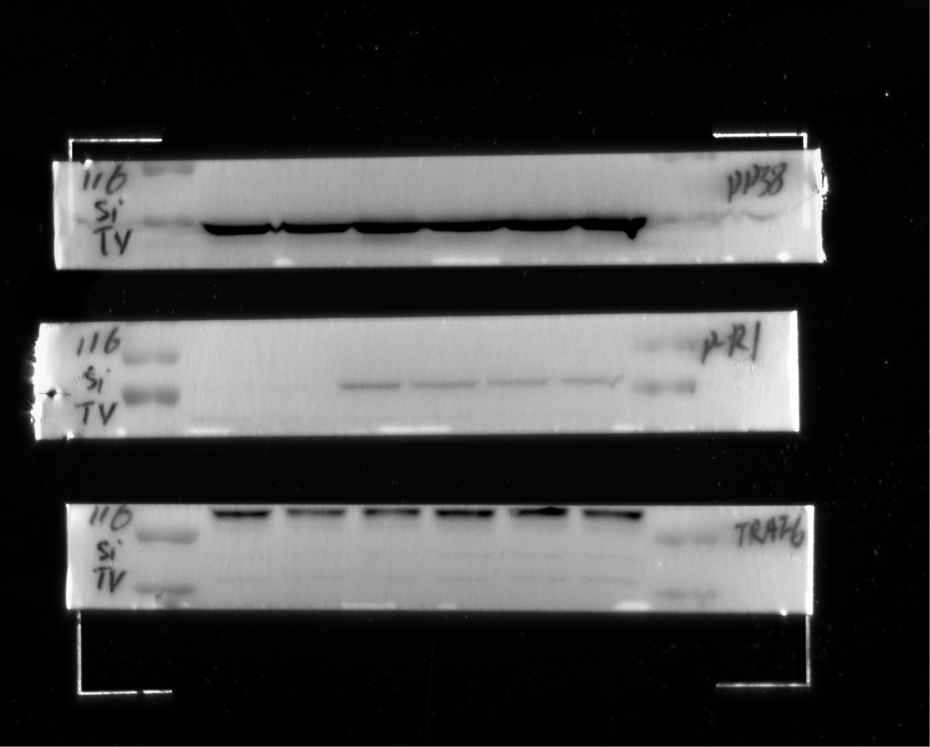


p-RIPK1 -78kDa


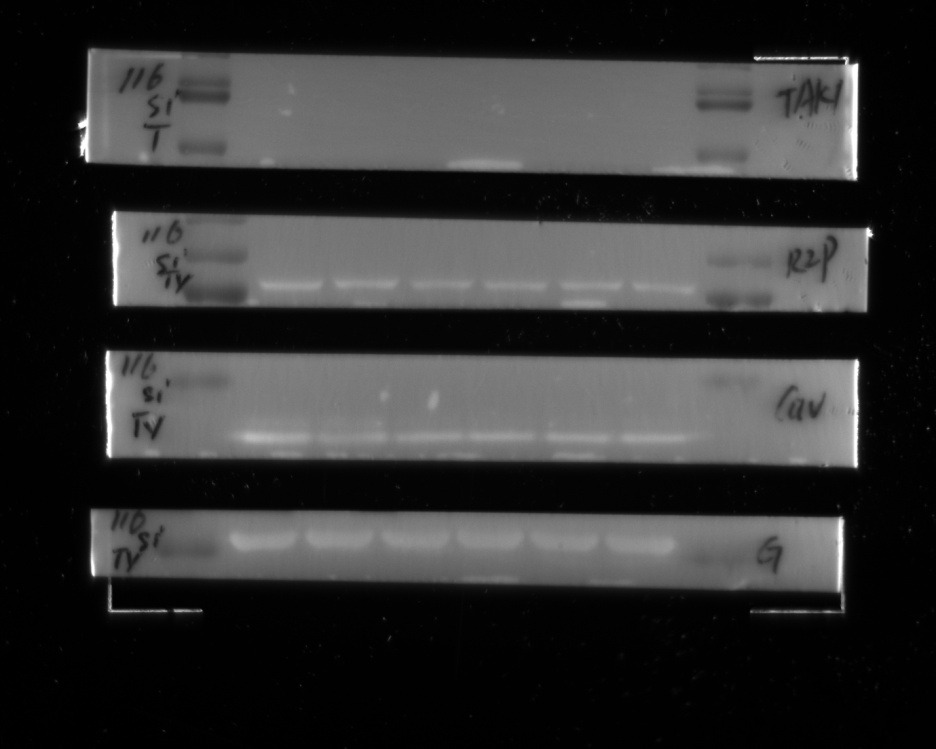


RIPK1 -78kDa

Figure 3I


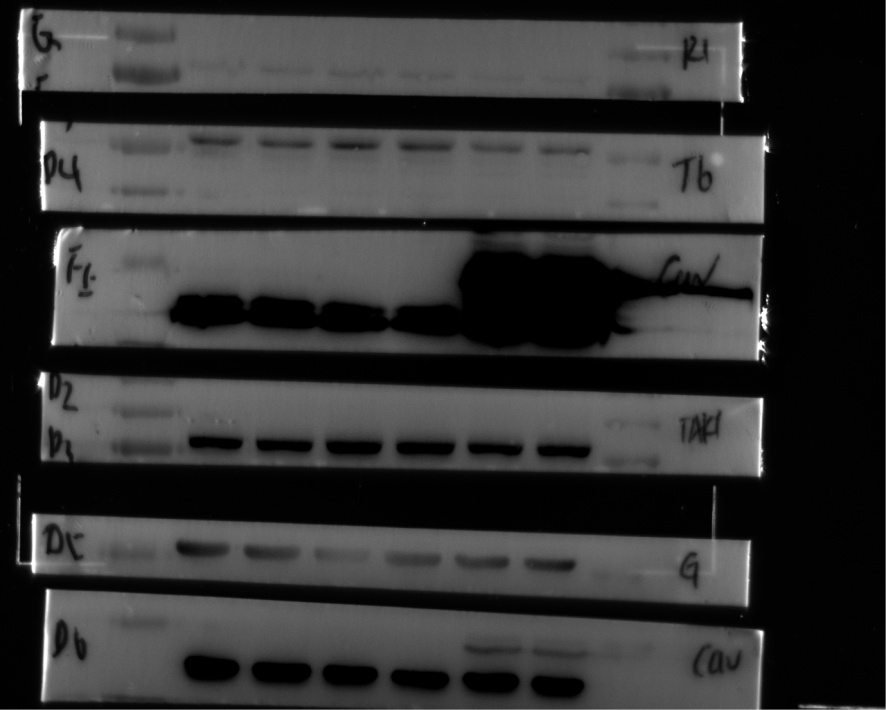


CAV1 -23kDa


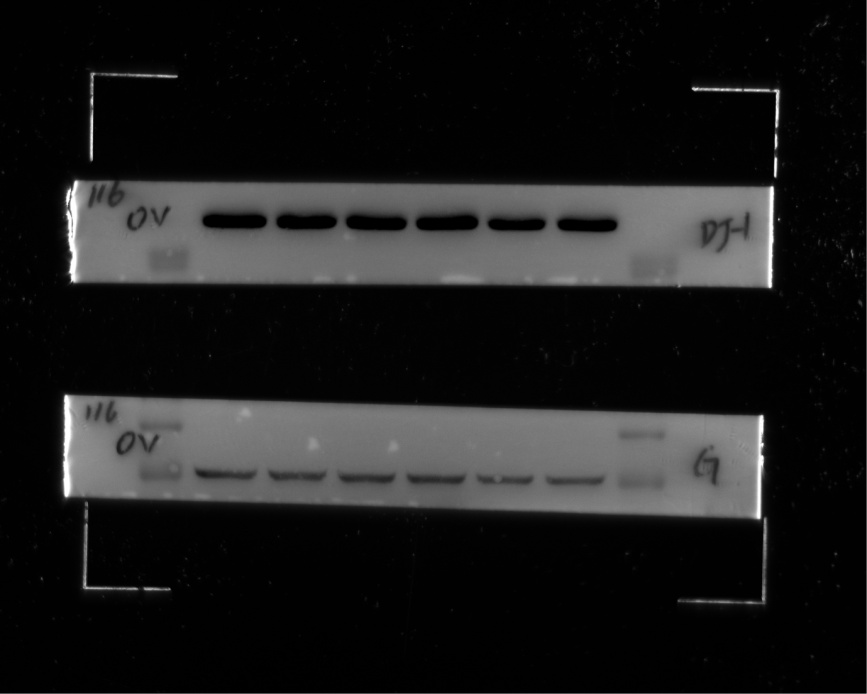


GAPDH -35kDa

DJ-1 -21kDa


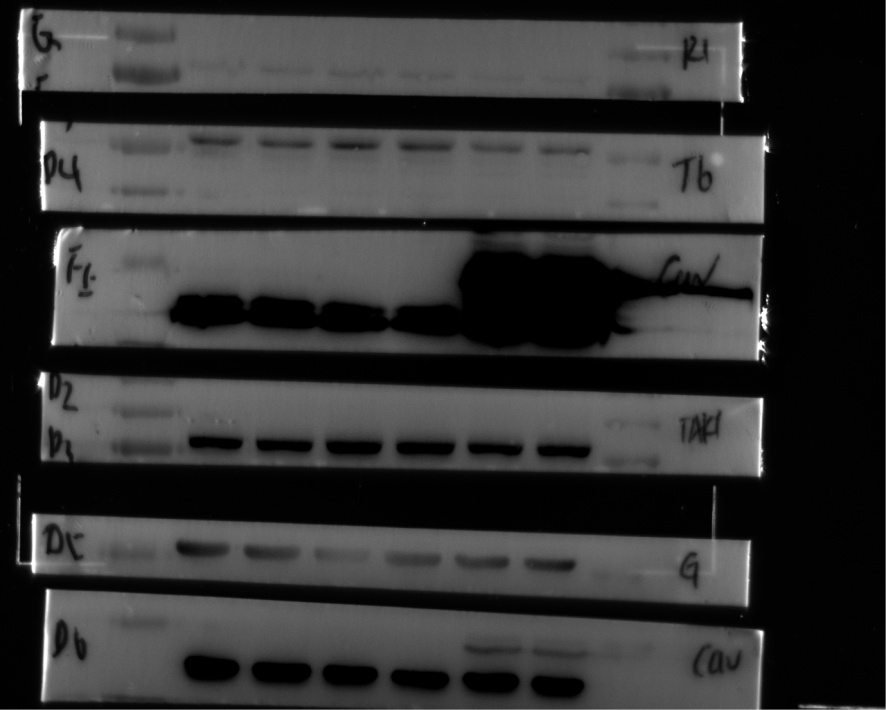


RIPK1 -78kDa


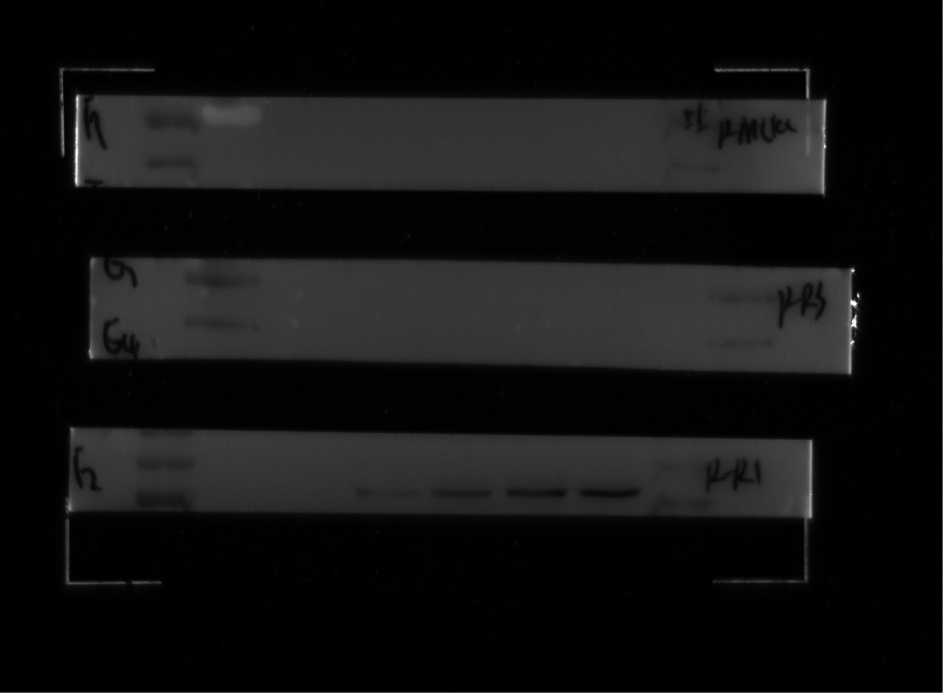


p-RIPK1 -78kDa

Figure 3J

**
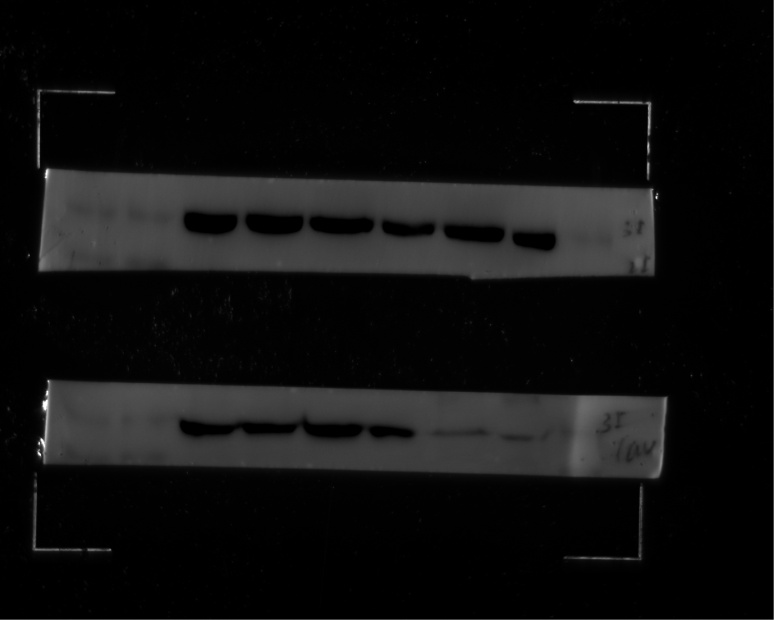
**

CAV1 -23kDa


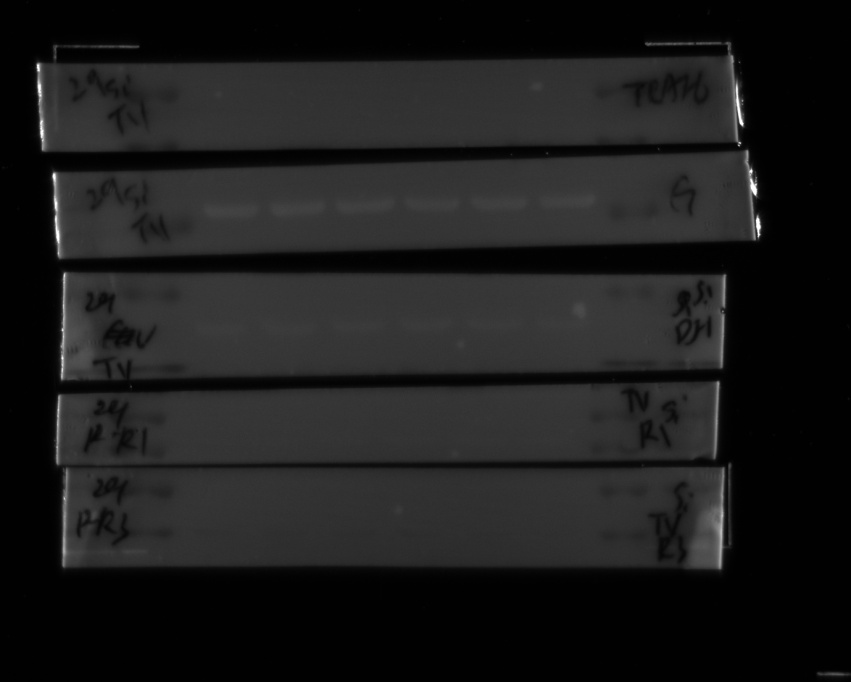


DJ-1 -21kDa


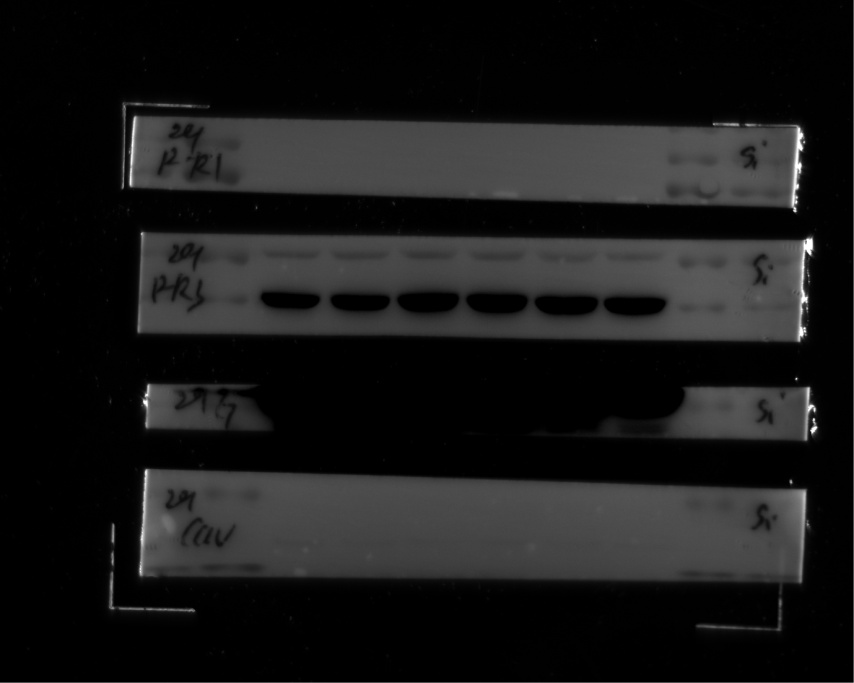


p-RIPK3 -46kDa

p-RIPK1 -78kDa


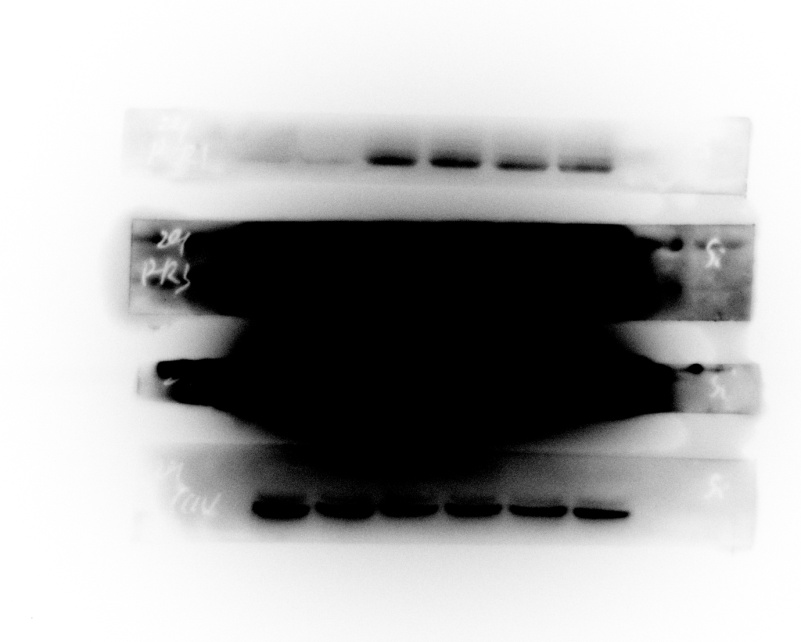


p-RIPK1 -78kDa


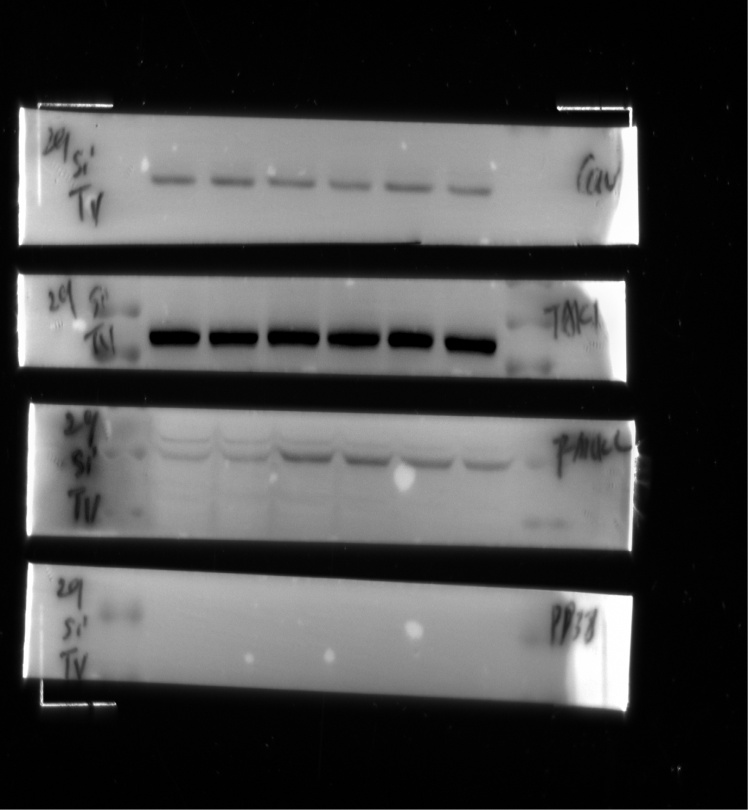


p-MLKL -54kDa


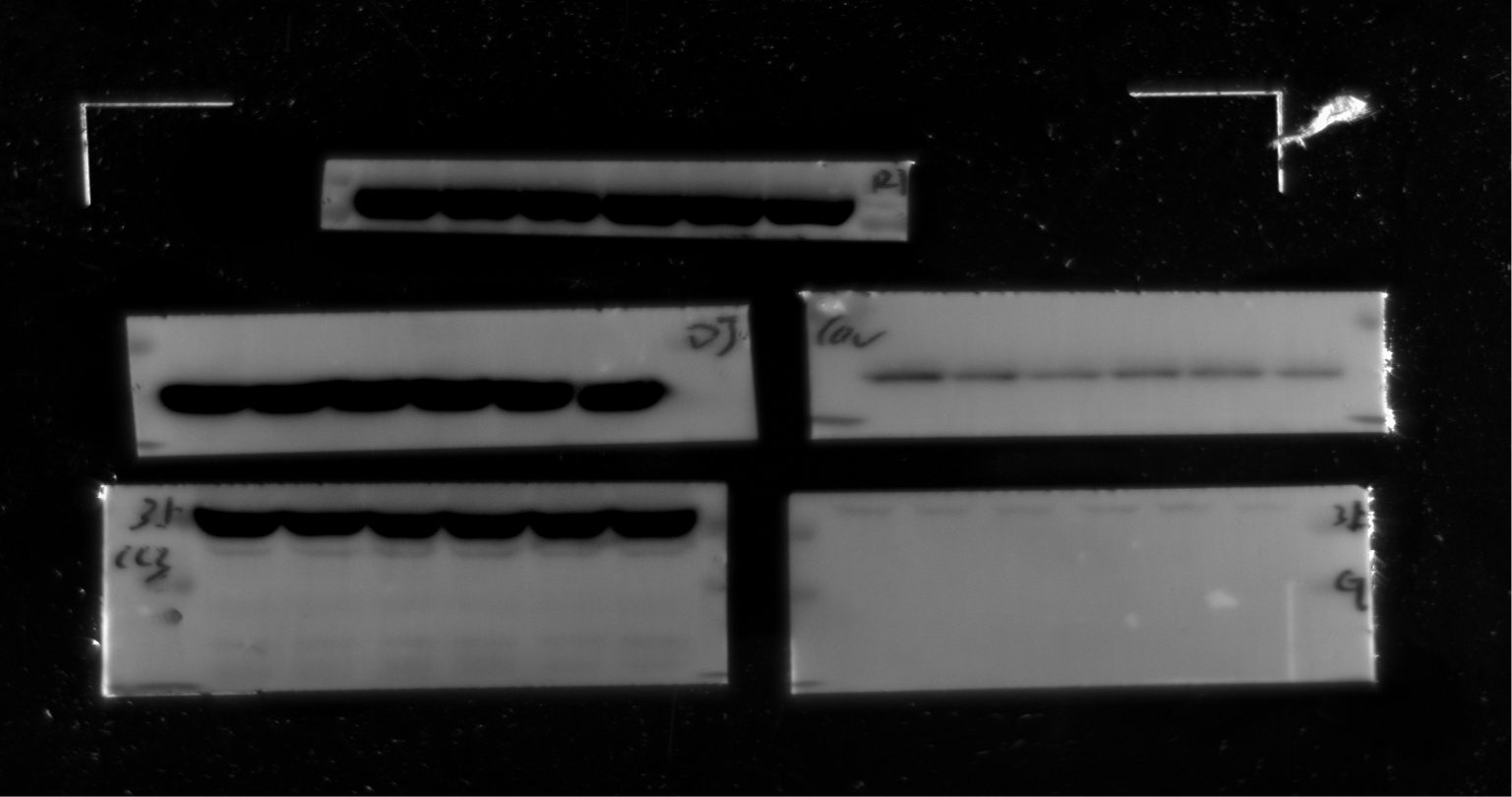


RIPK1 -78kDa


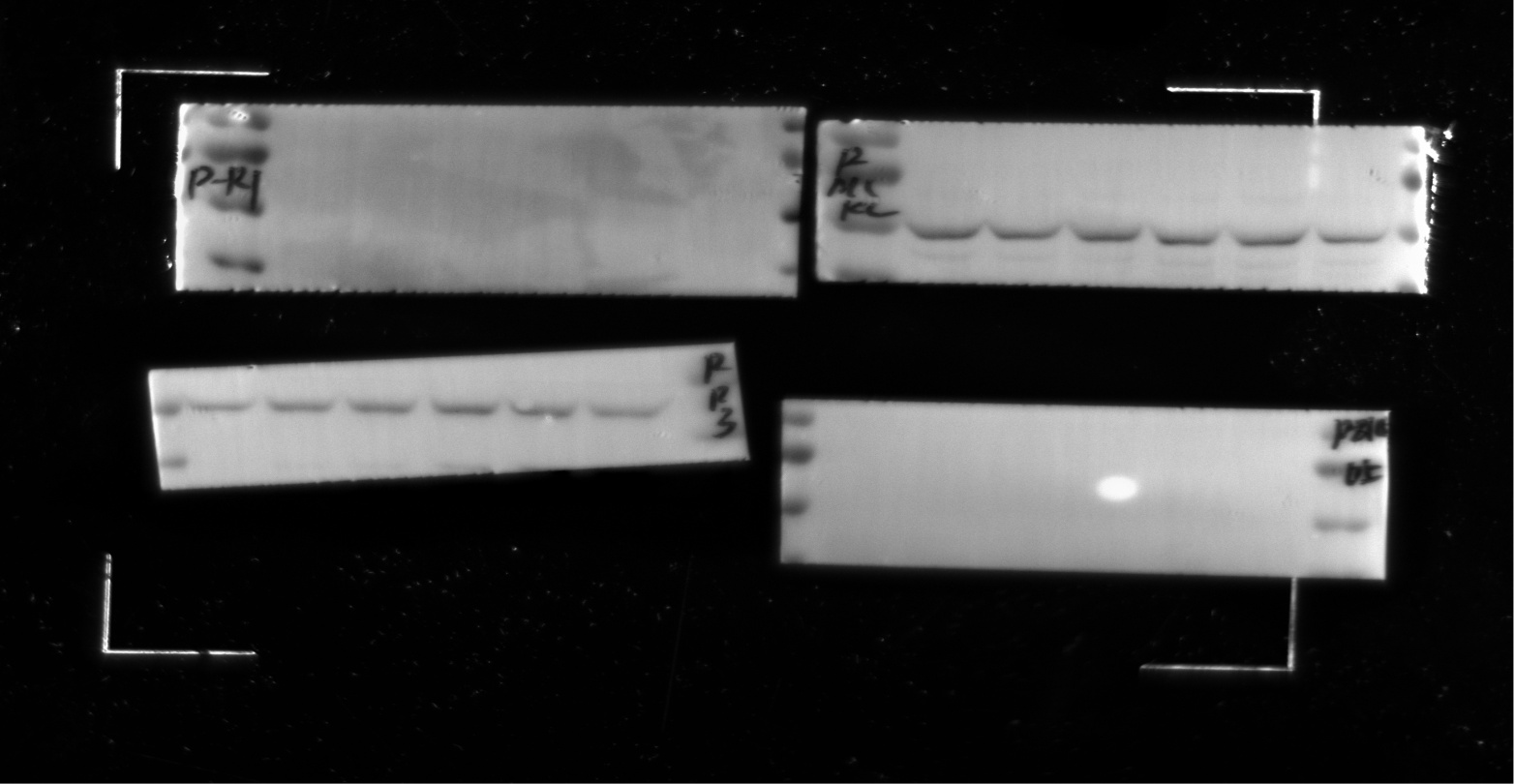


MLKL -54kDa


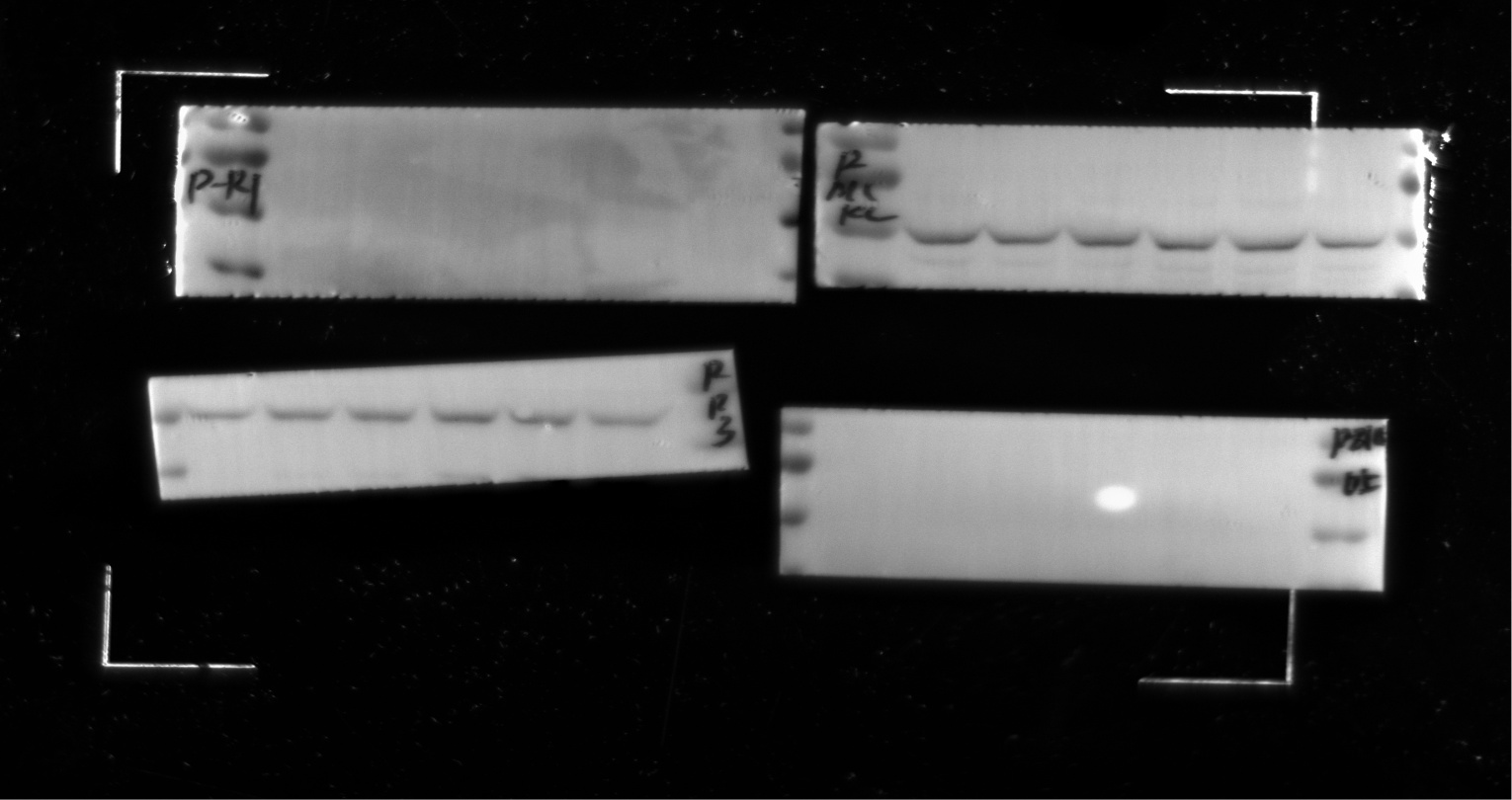


DJ-1 -21kDa

RIPK3 -46kDa


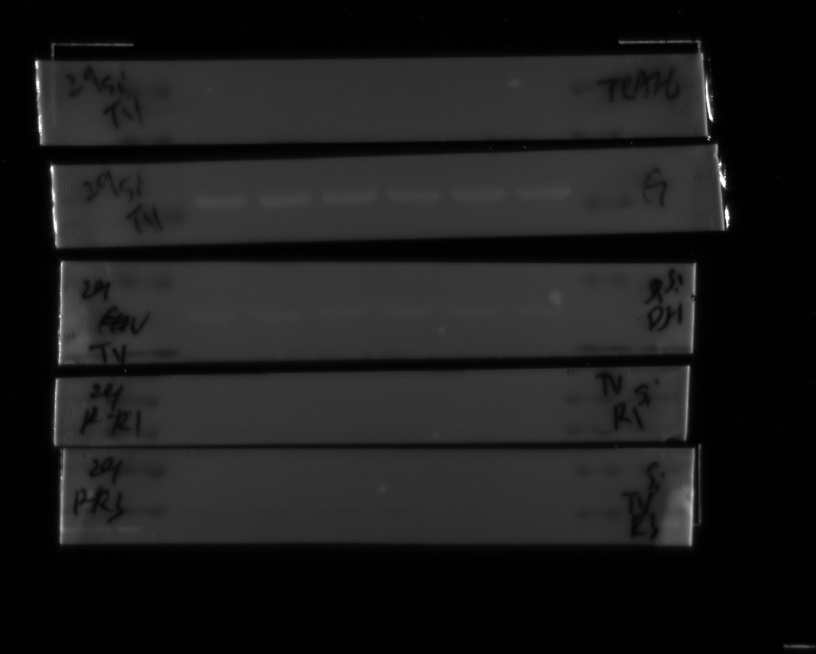


GAPDH -35kDa

Figure 4D

IP

Input

CAV1 -23kDa


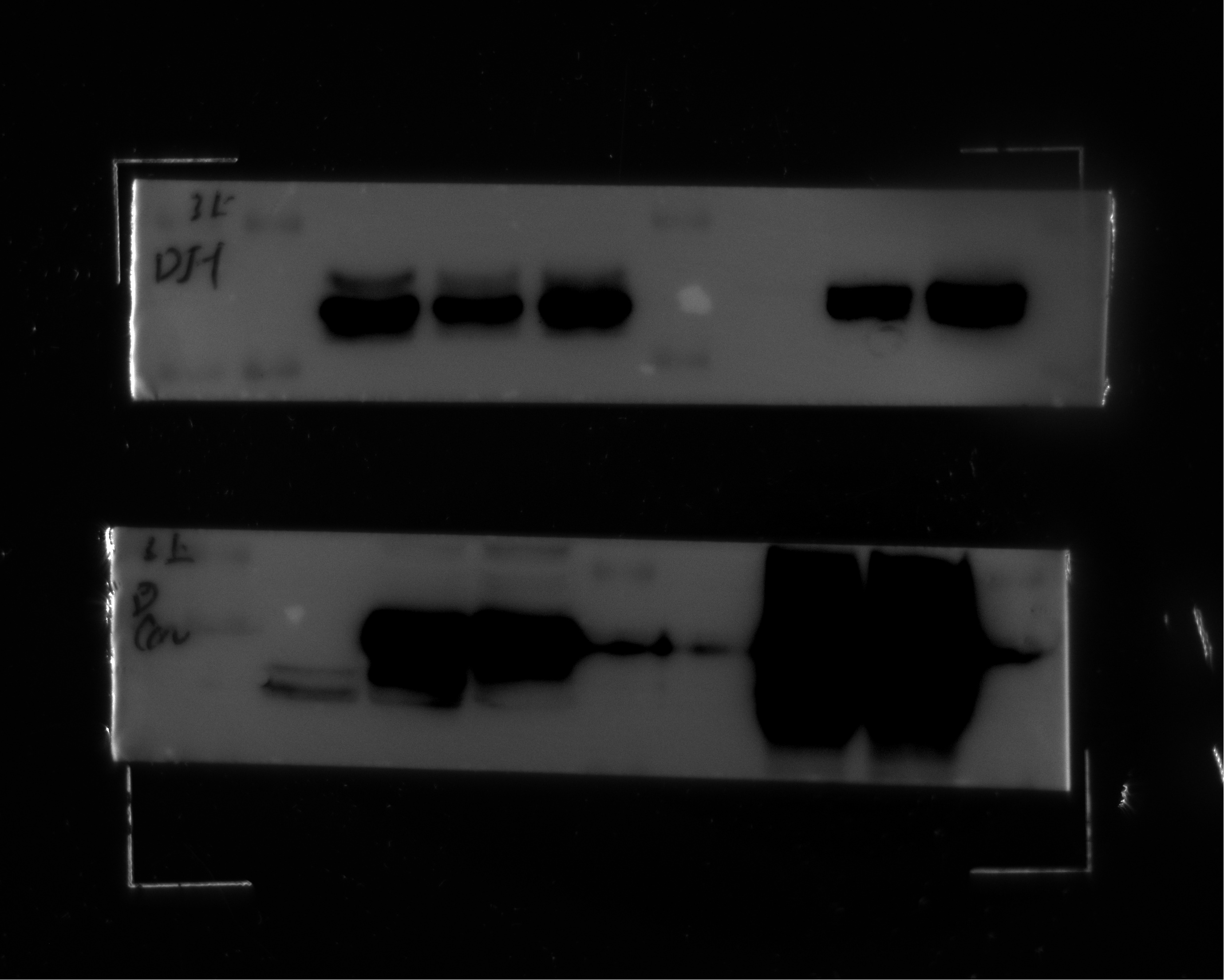


Figure 4E

IP

Input


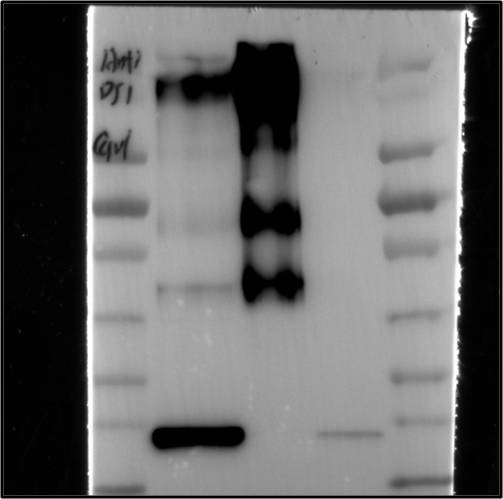


CAV1 -23kDa


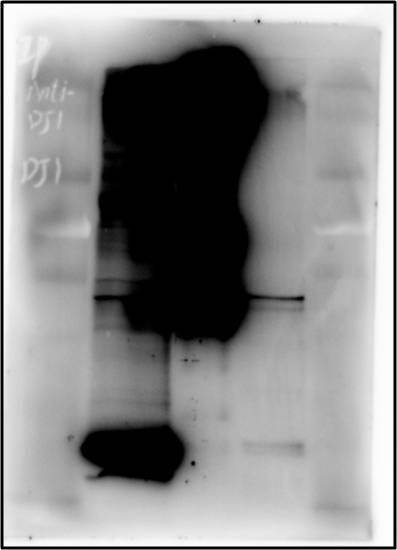


DJ-1 -21kDa

Figure 4F


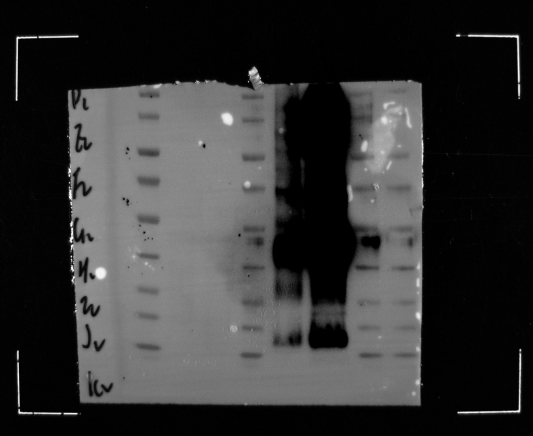


IP:CAV1 -23kDa


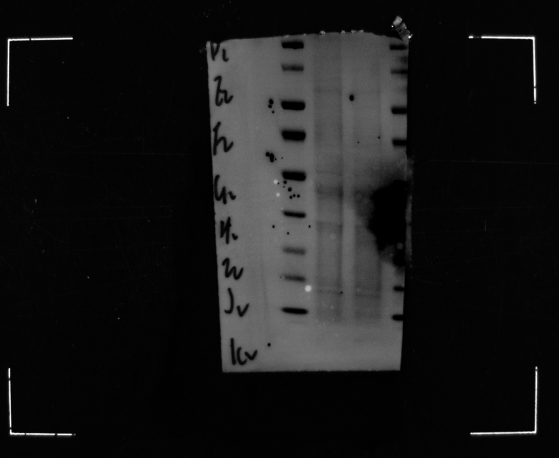


Input CAV1 -23kDa


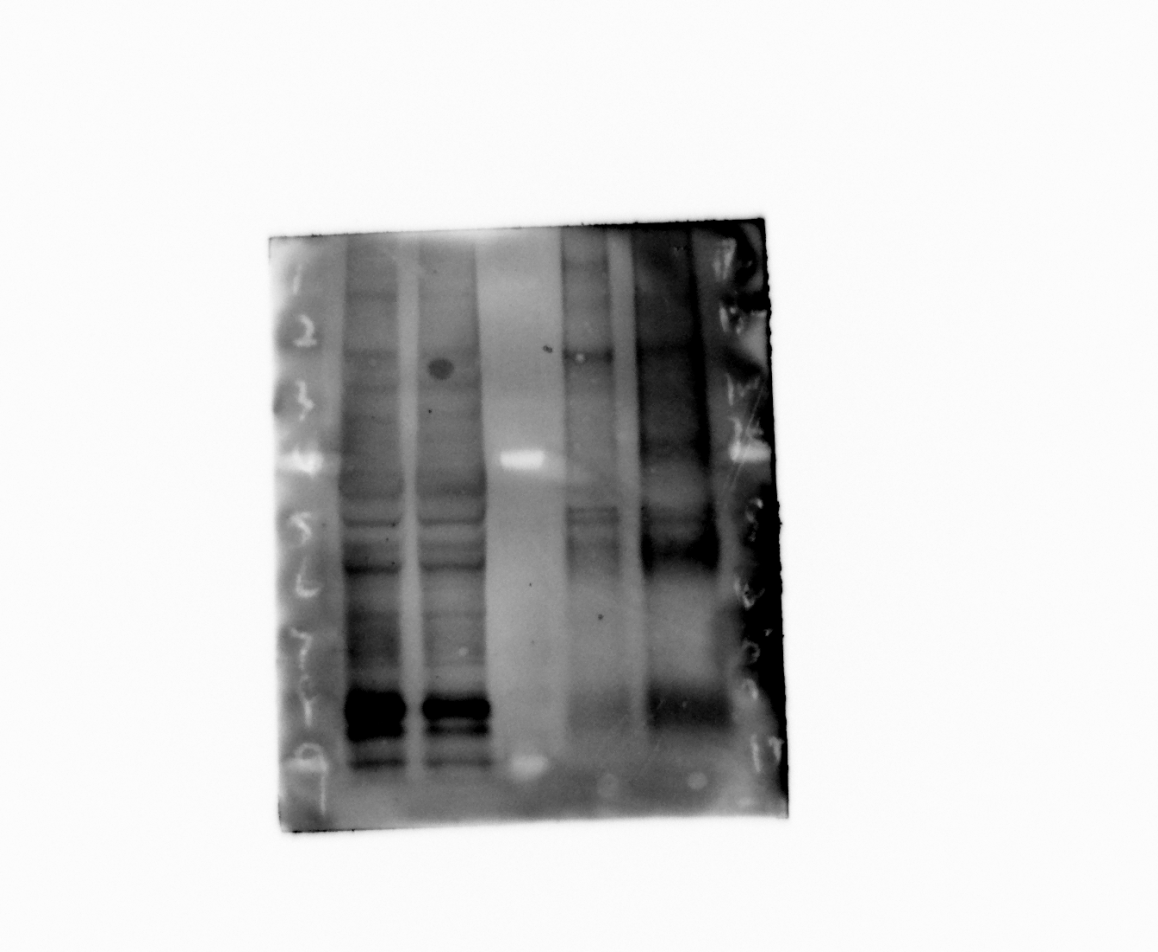


Input and IP: DJ-1 -21kDa

Figure 4H


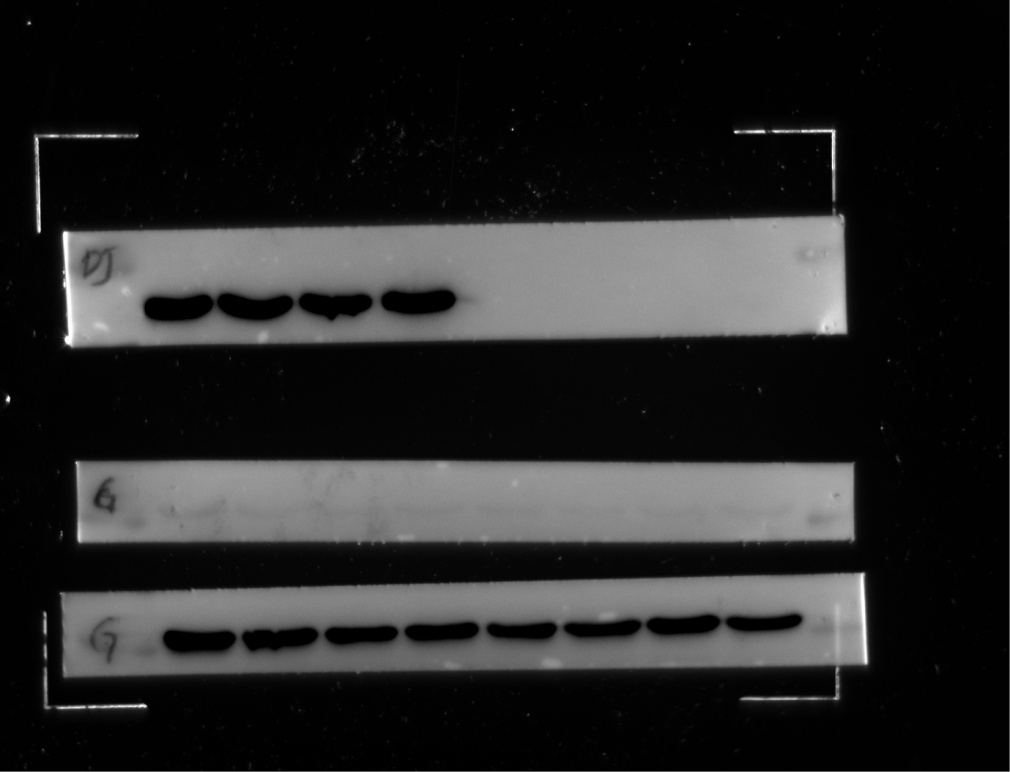


GAPDH -35kDa

DJ-1 -21kDa


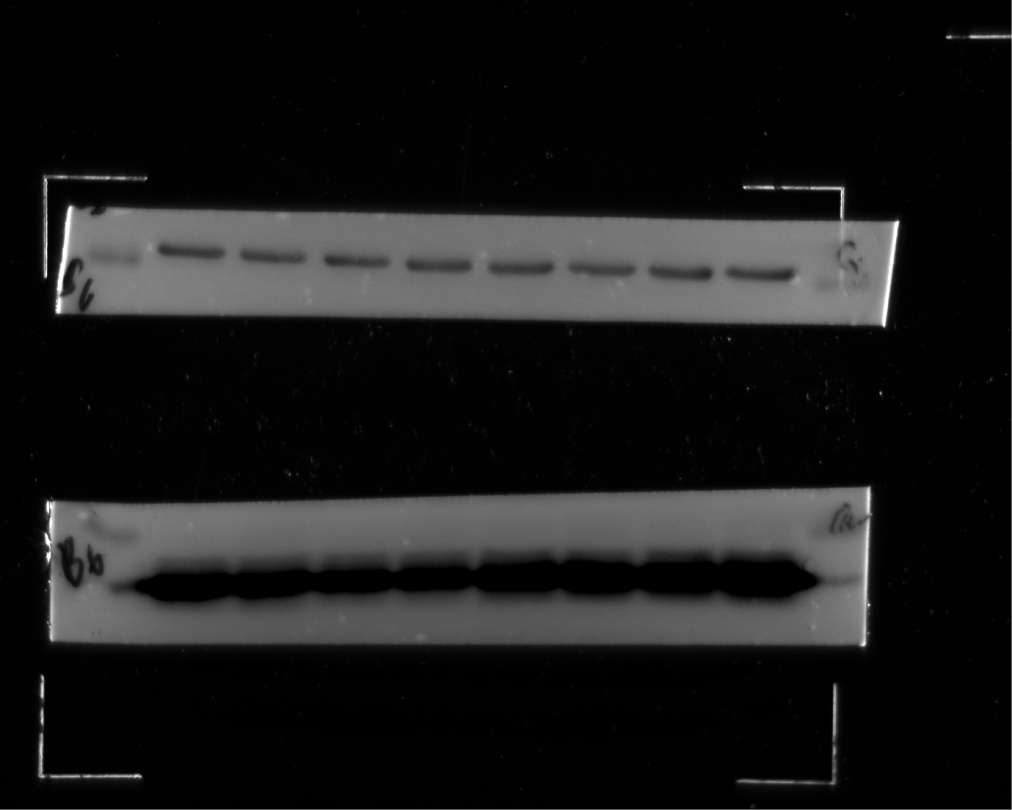


CAV1 -23kDa

Figure 4J


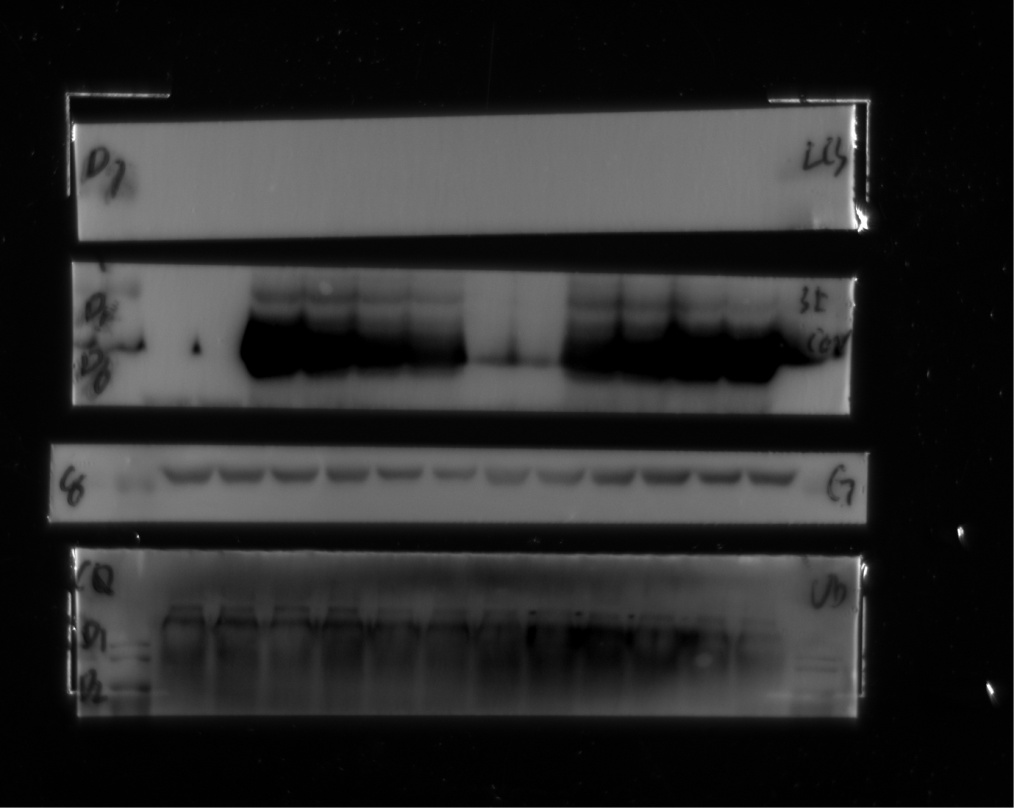


GAPDH -35kDa

CAV1 -23kDa


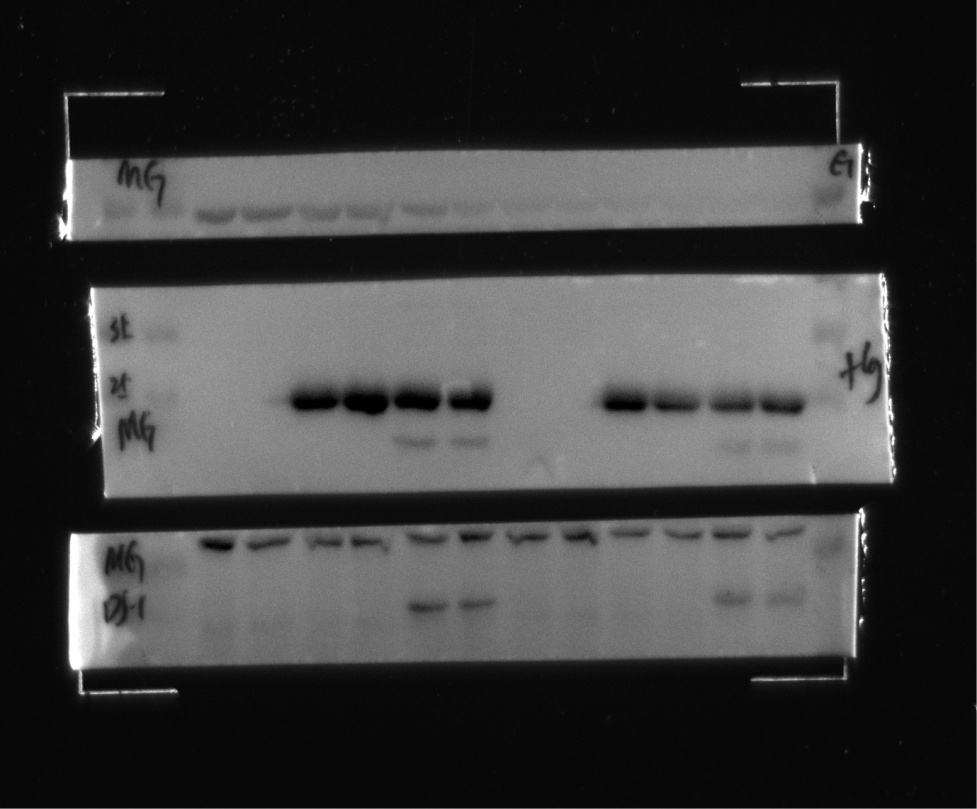


HA -21kDa


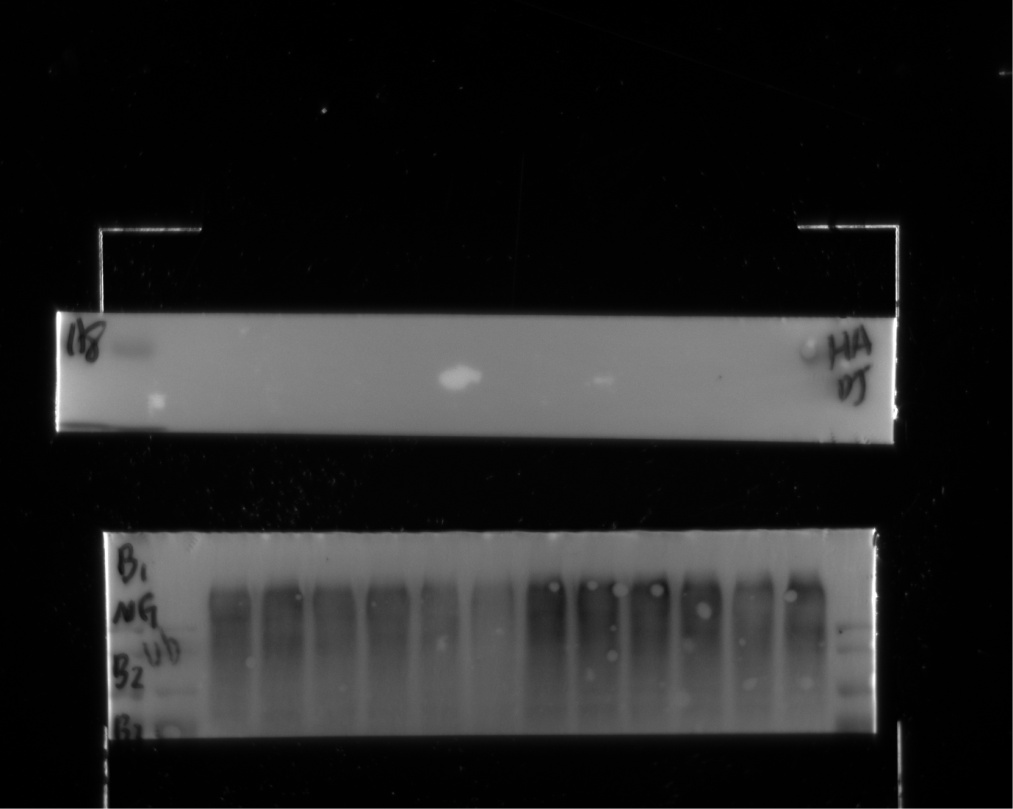


Ubiquitin -170kDa

Figure 6C


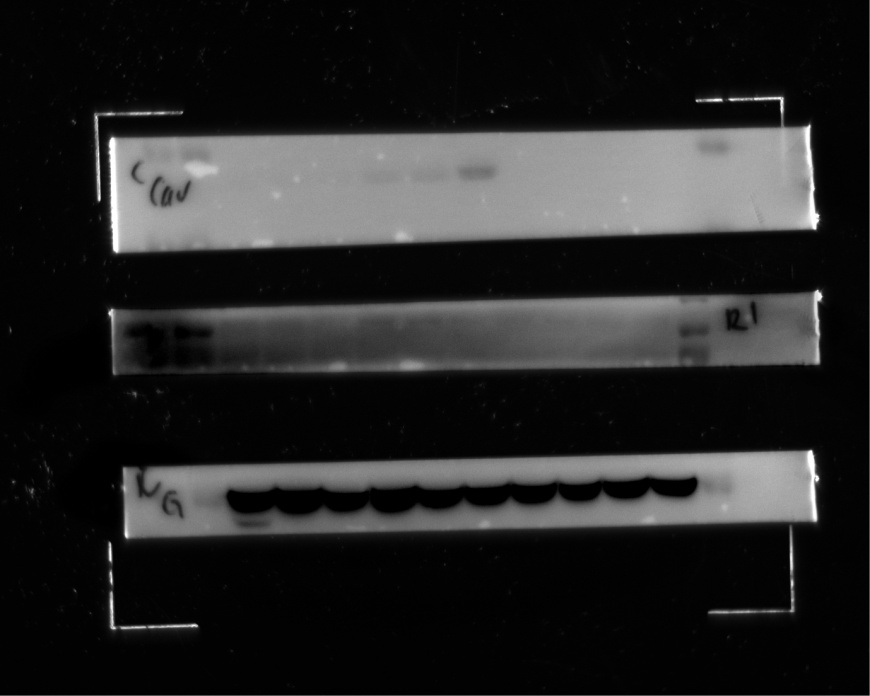


Repeat-1 GAPDH -35kDa

Repeat-1 CAV1 -23kDa

DJ-1 -21kDa


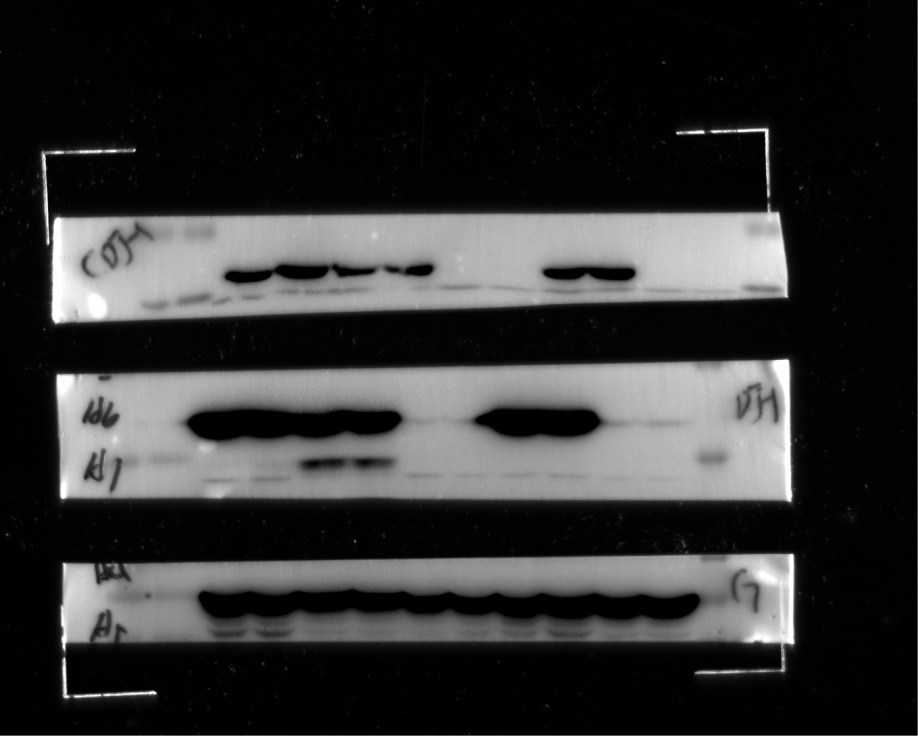


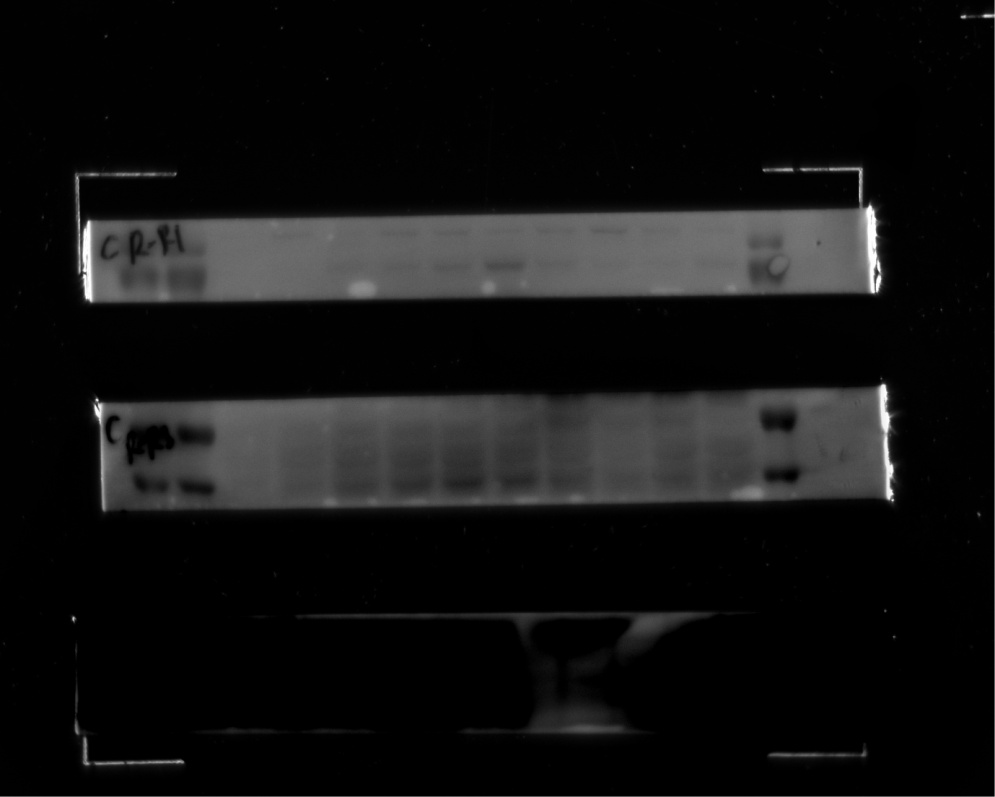


p-RIPK1 -78kDa


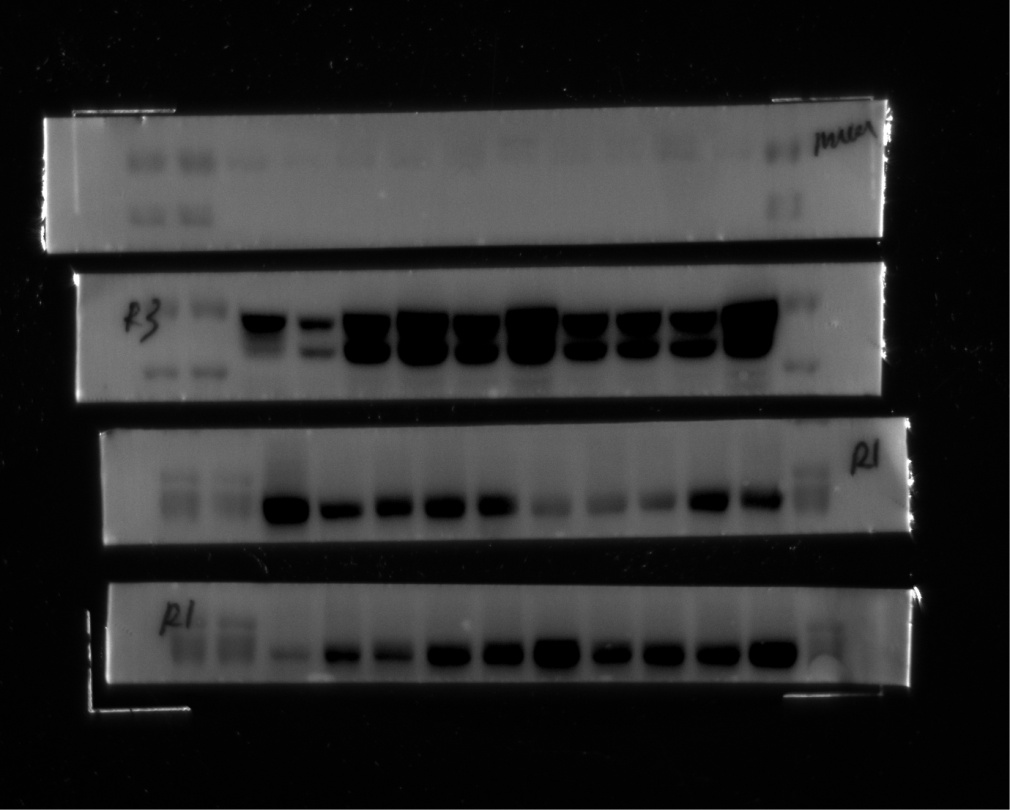


RIPK1 -78kDa


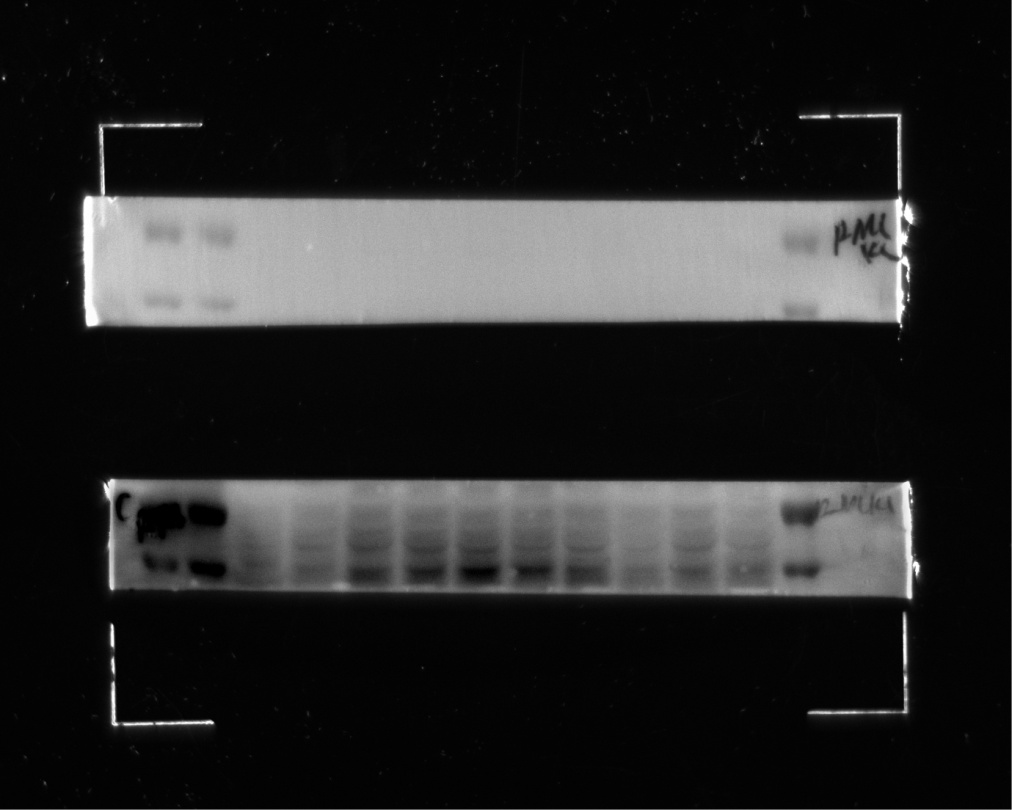


p-RIPK3 -46kDa


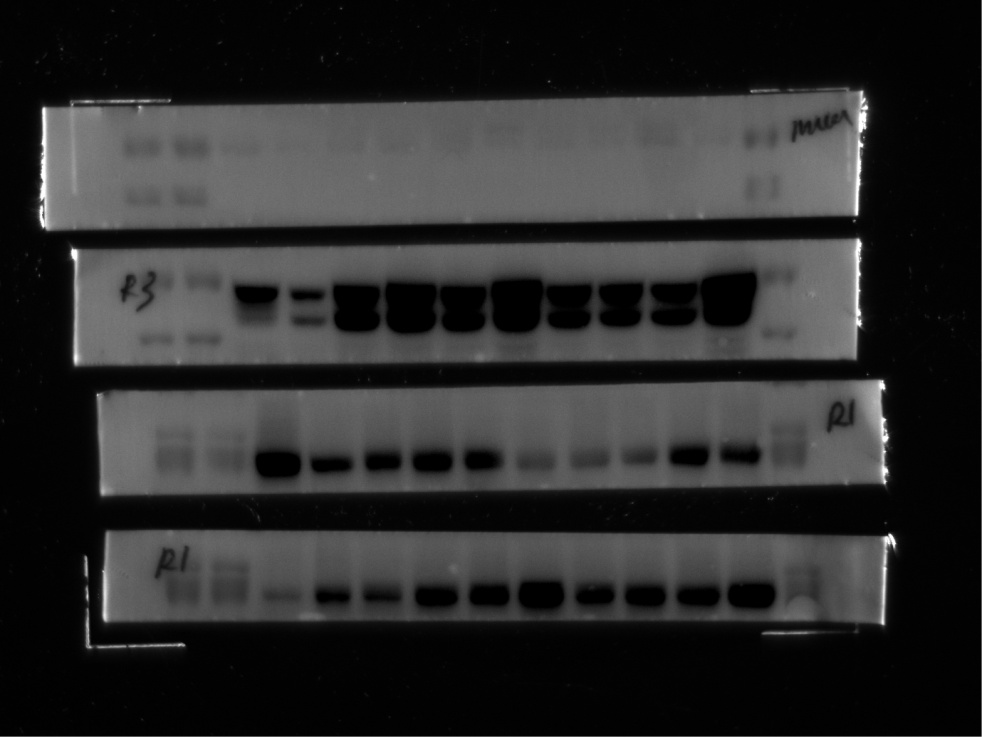


RIPK3 -46kDa


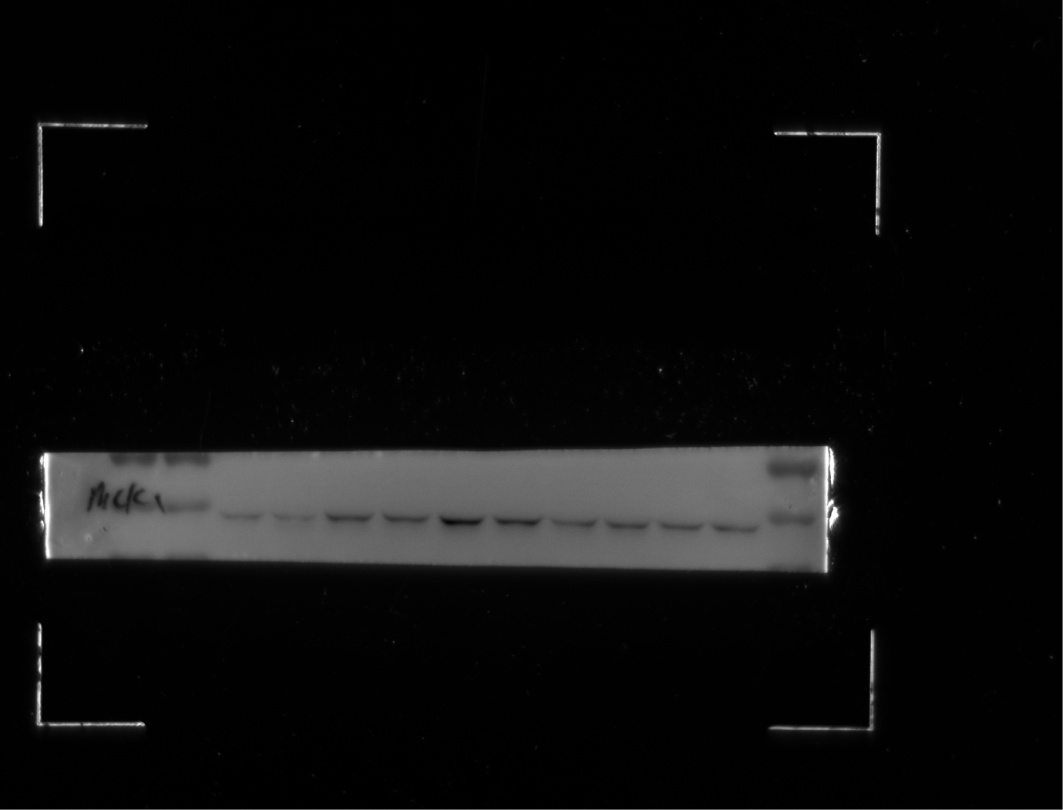


p-MLKL -54kDa


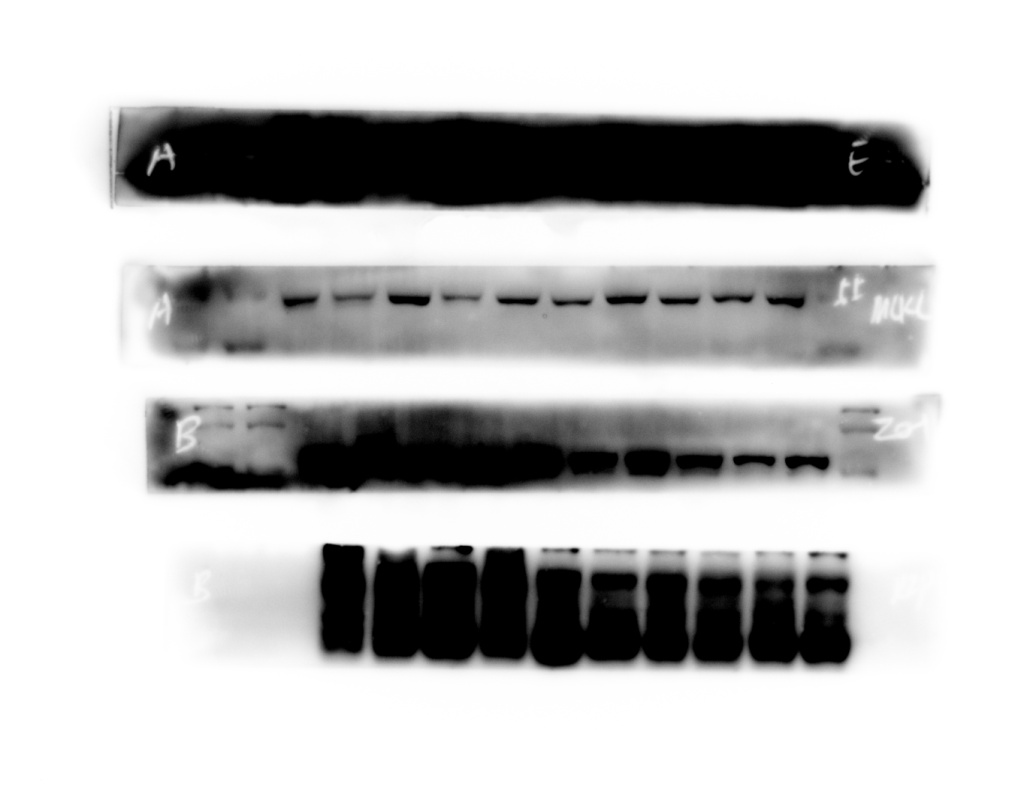


MLKL -54kDa

Repeat-2 GAPDH -35kDa


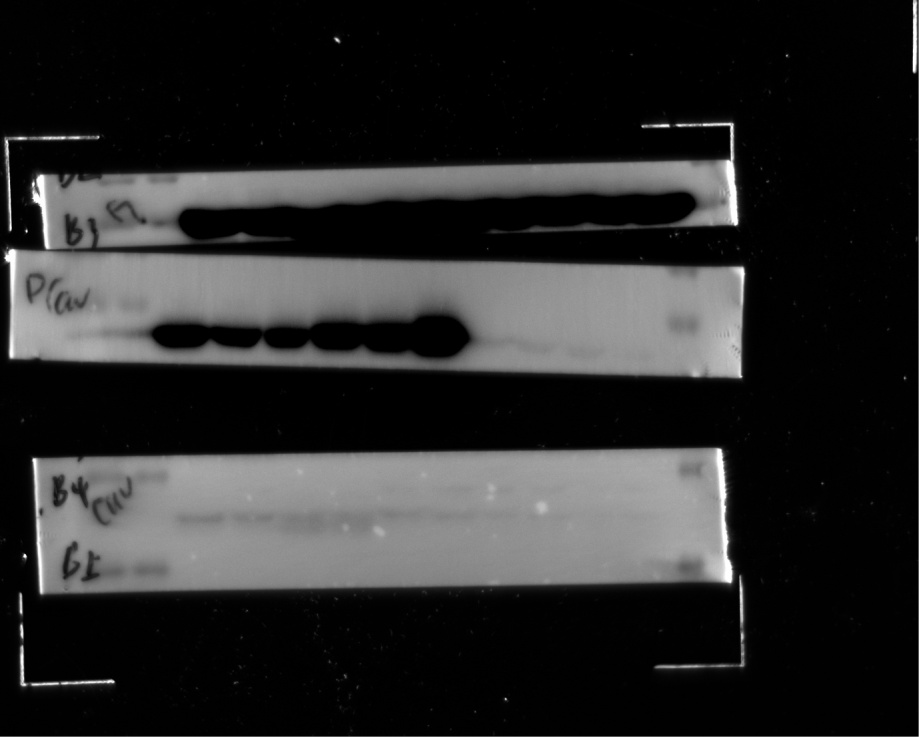


Repeat-2 CAV1 -23kDa


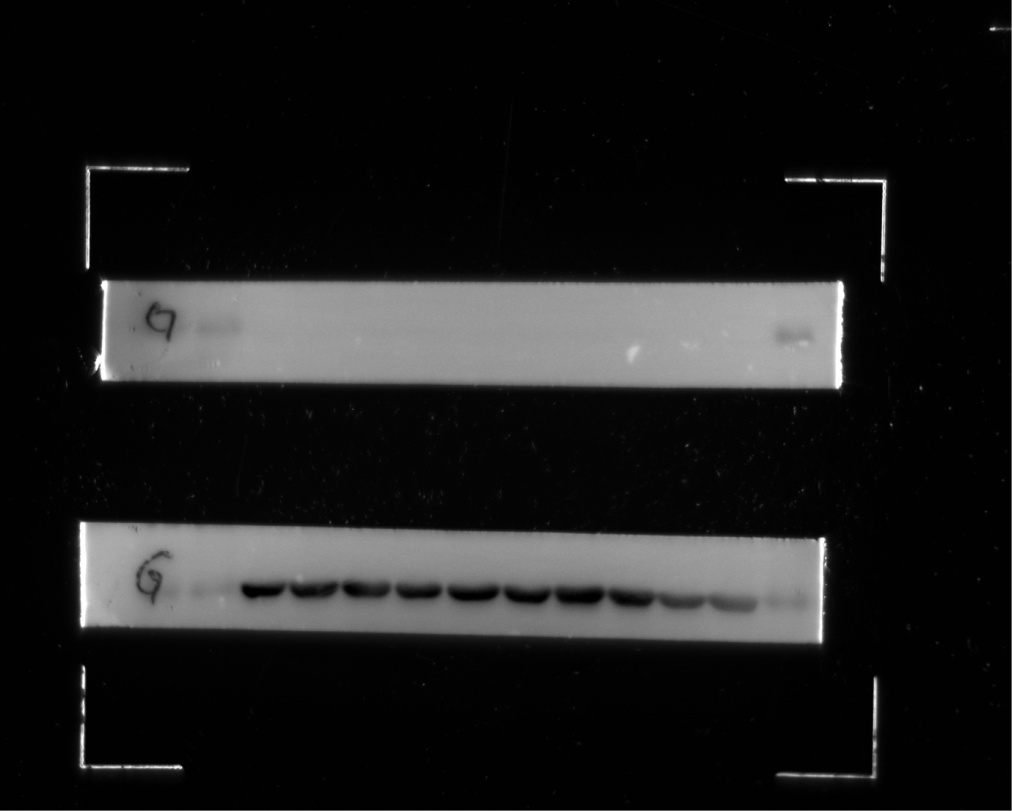


Repeat-3 CAV1 -23kDa

Repeat-3 GAPDH -35kDa


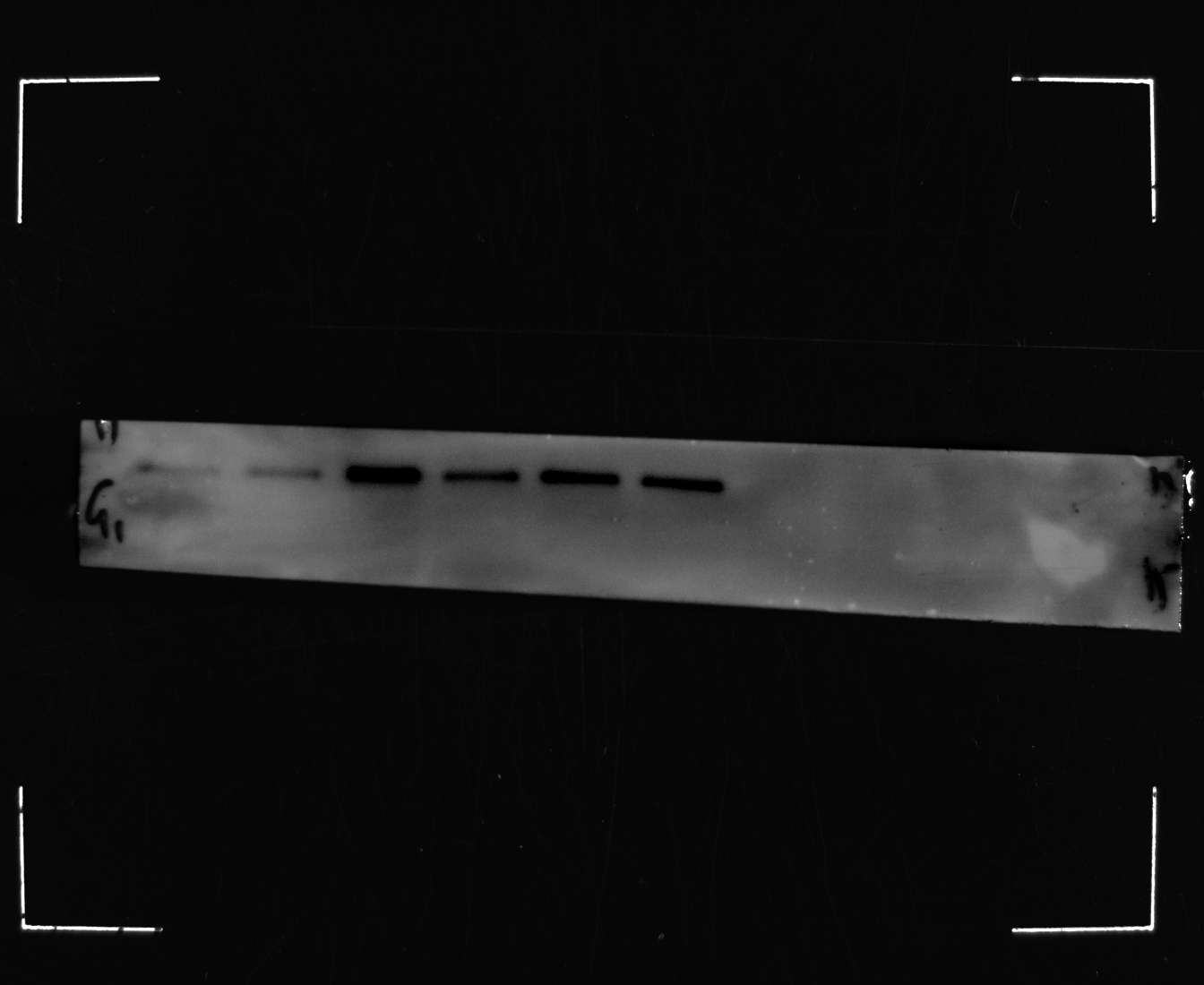

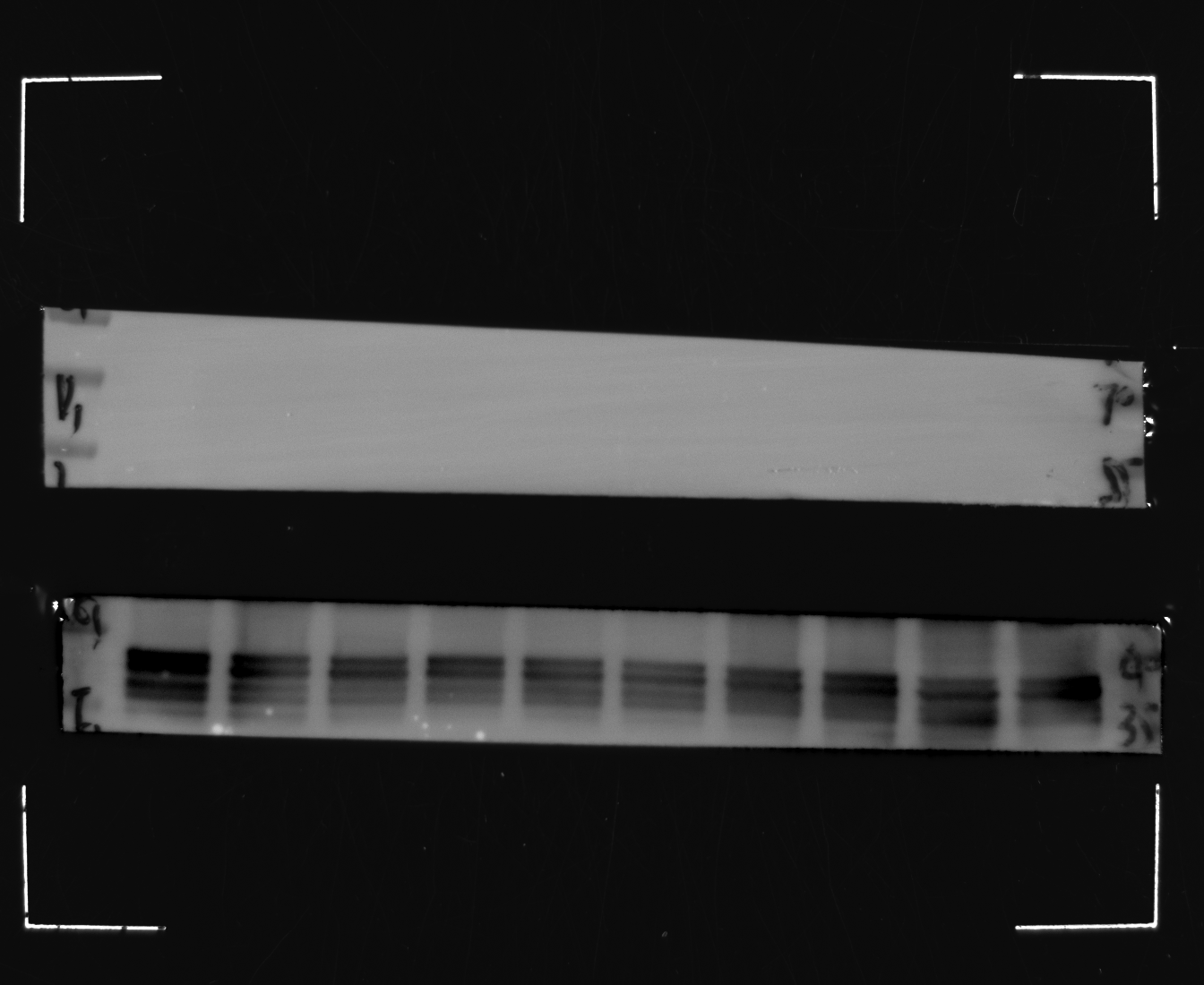


Figure 6F


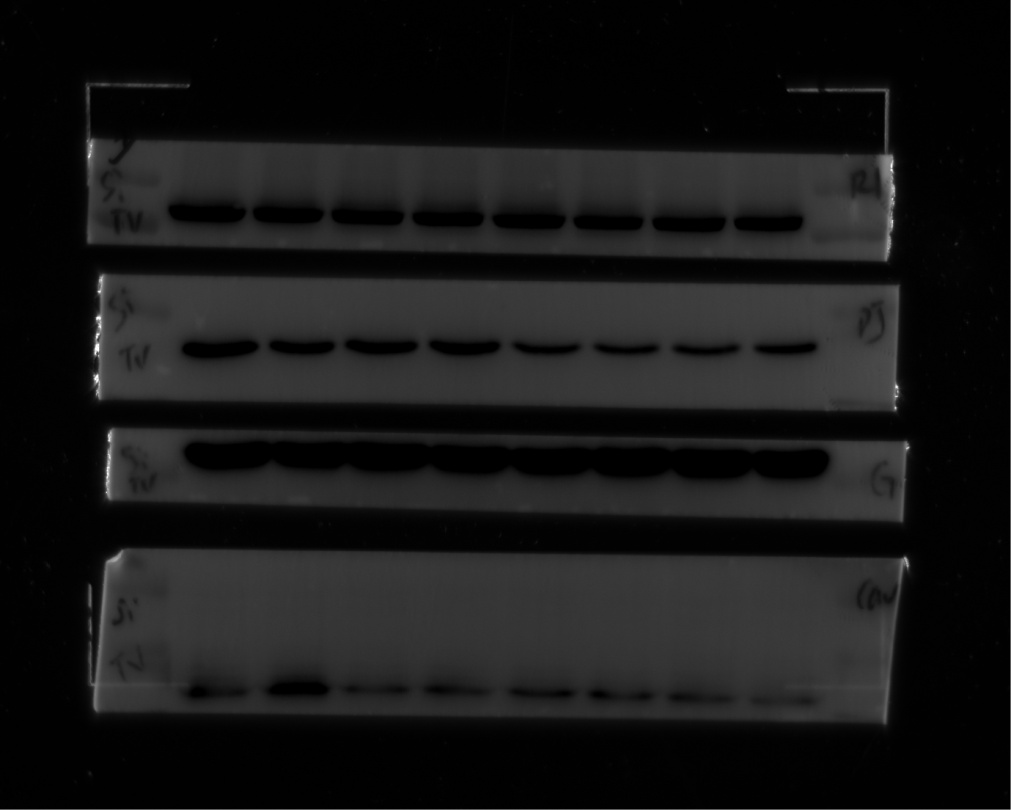


GAPDH -35kDa

CAV1 -23kDa

DJ-1 -21kDa

RIPK1 -78kDa


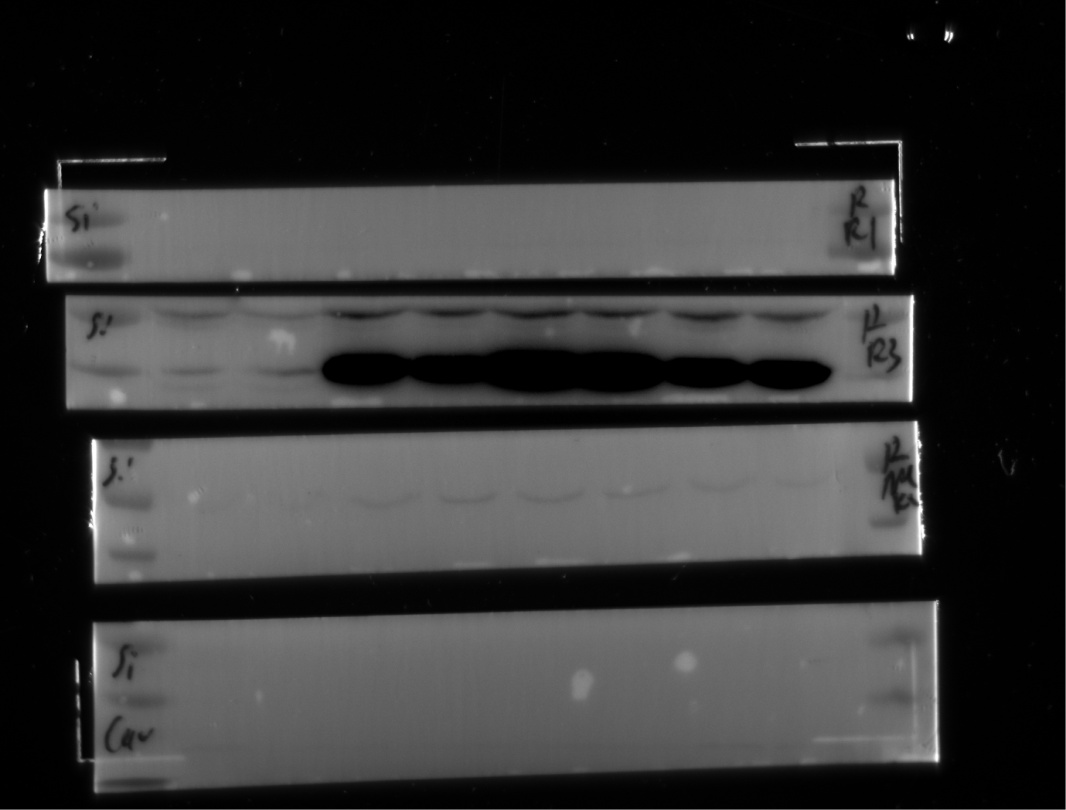


p-RIPK1 -78kDa

Figure 6G


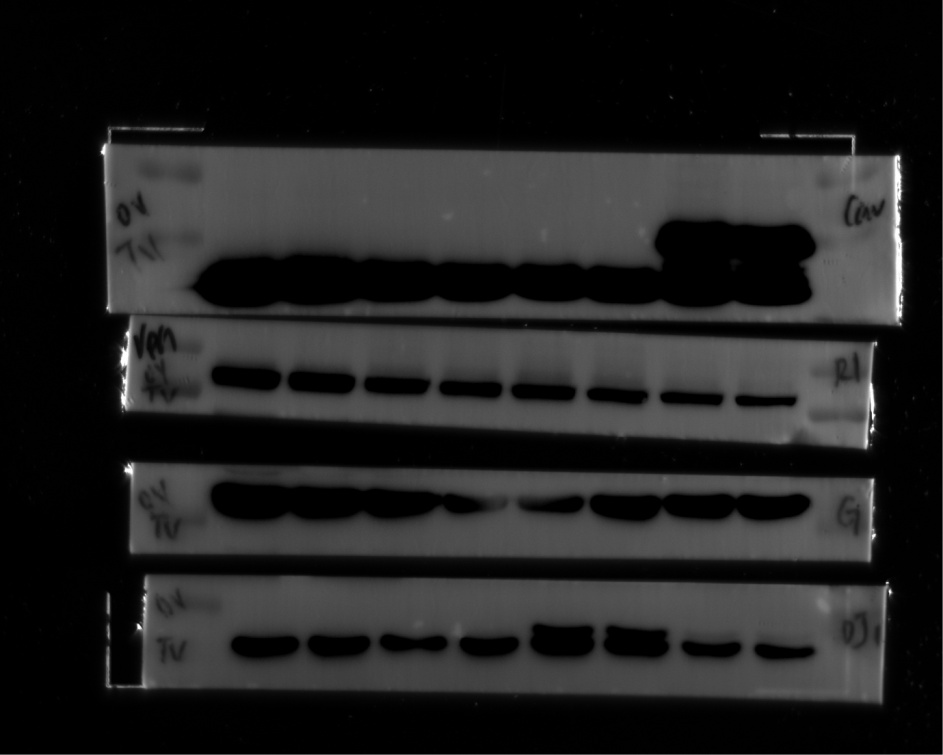


RIPK1 -78kDa

CAV1 -23kDa


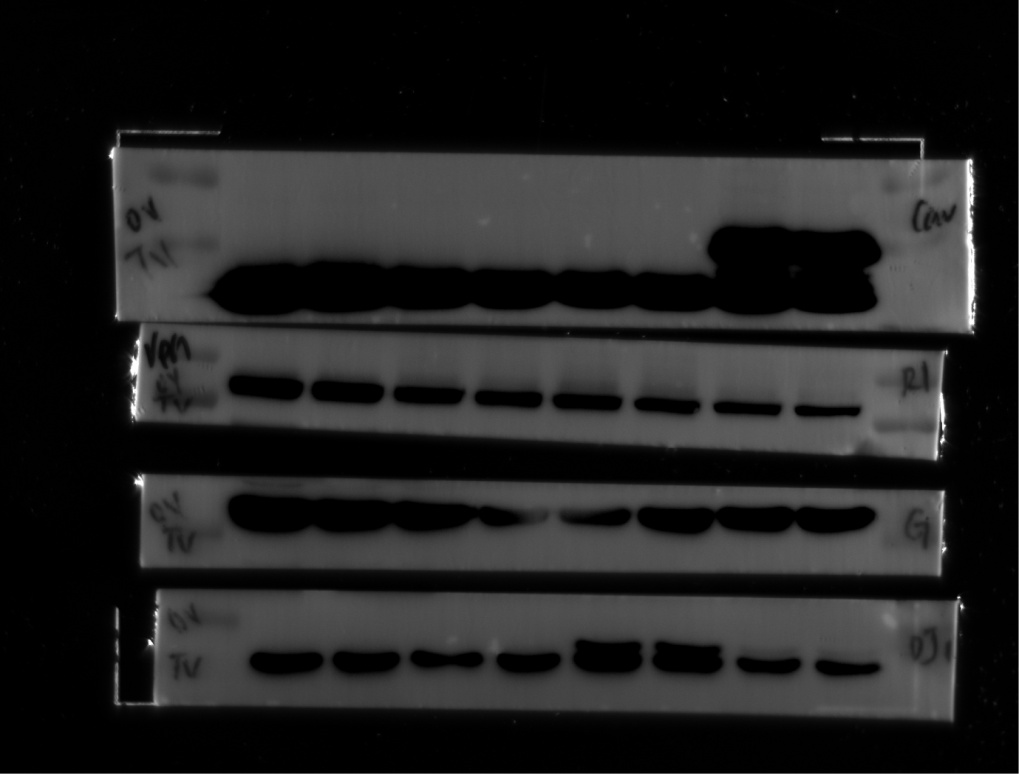


DJ-1 -21kDa


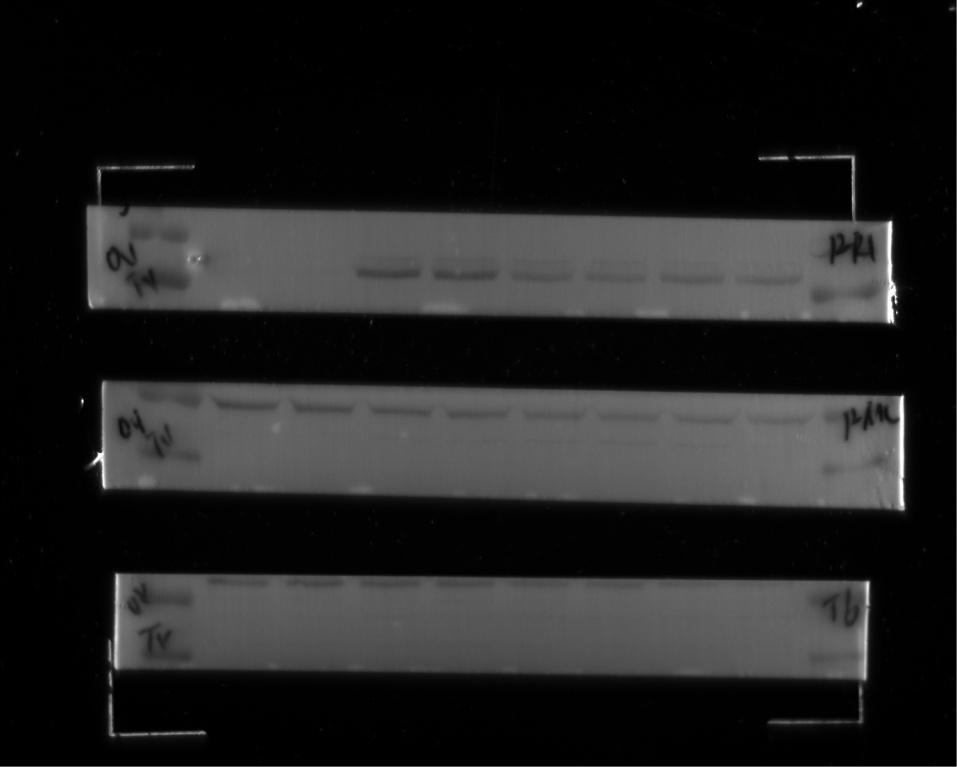


p-RIPK1 -78kDa


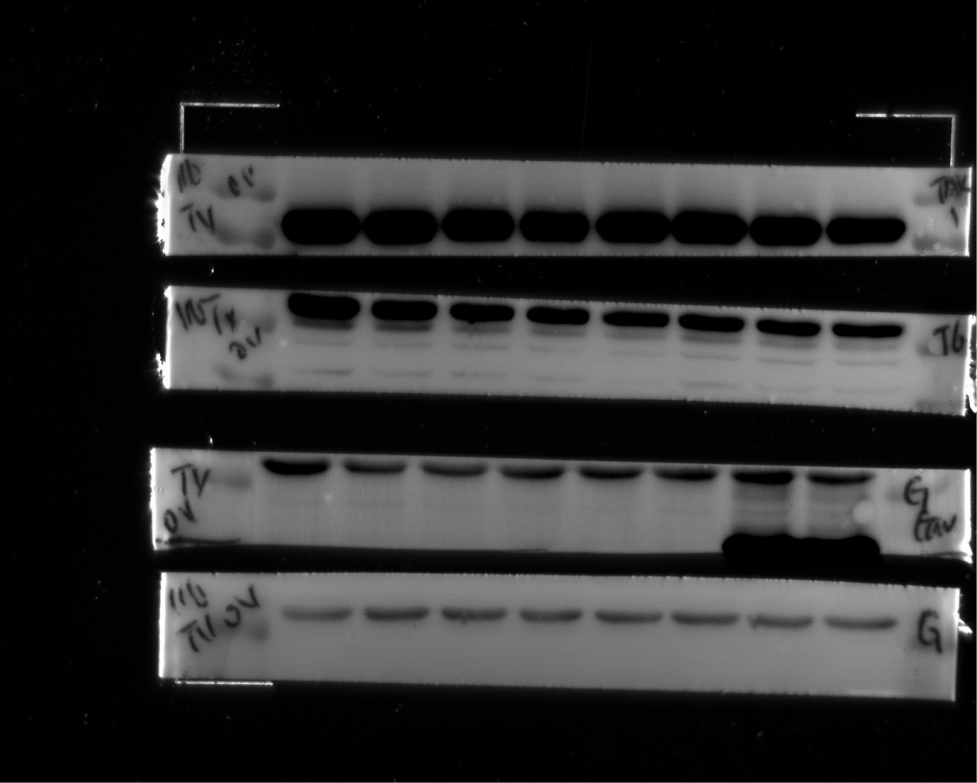


GAPDH -35kDa

**Supplementary Figure 1G**


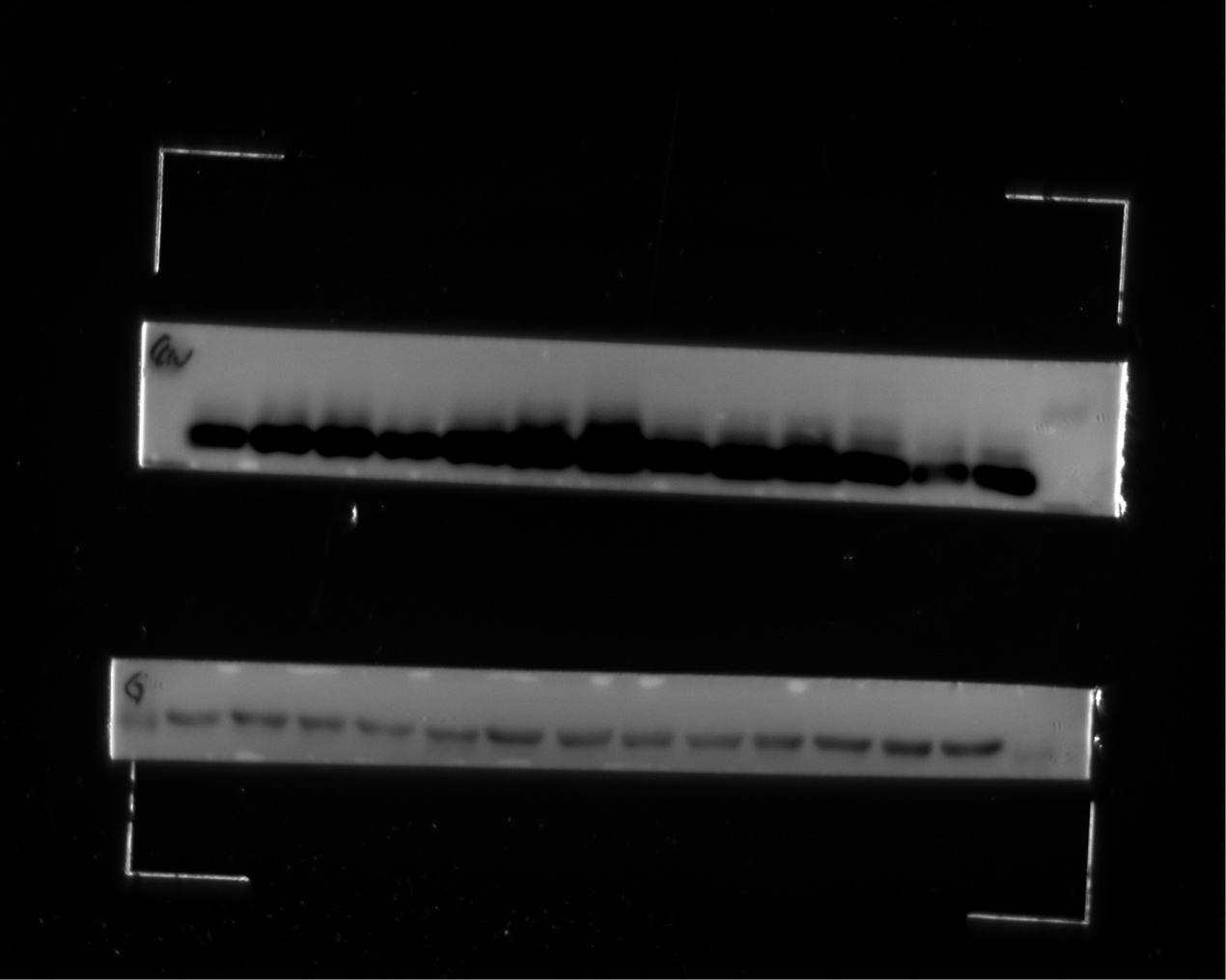


CAV1 -23kDa

GAPDH -35kDa

**Supplementary Figure 3C**

CAV1 -23kDa


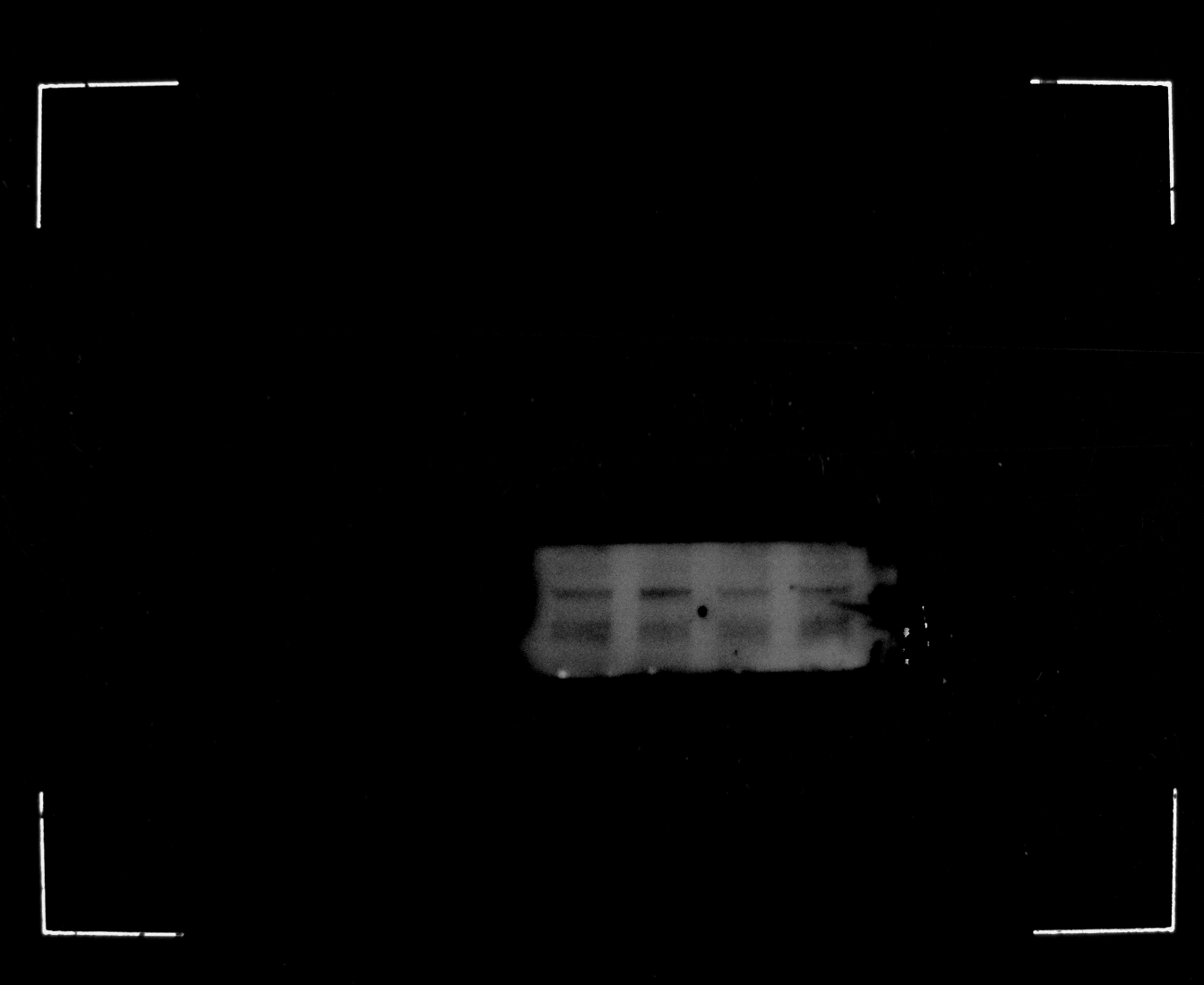


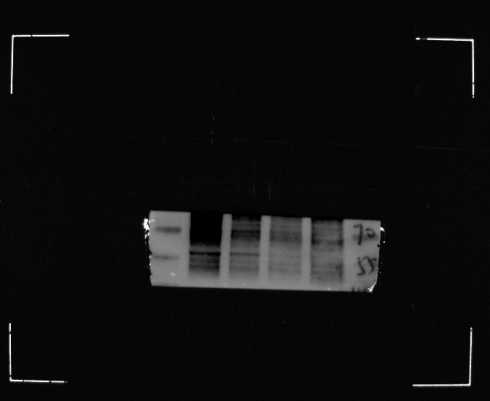


p-MLKL -54kDa

MLKL -54kDa


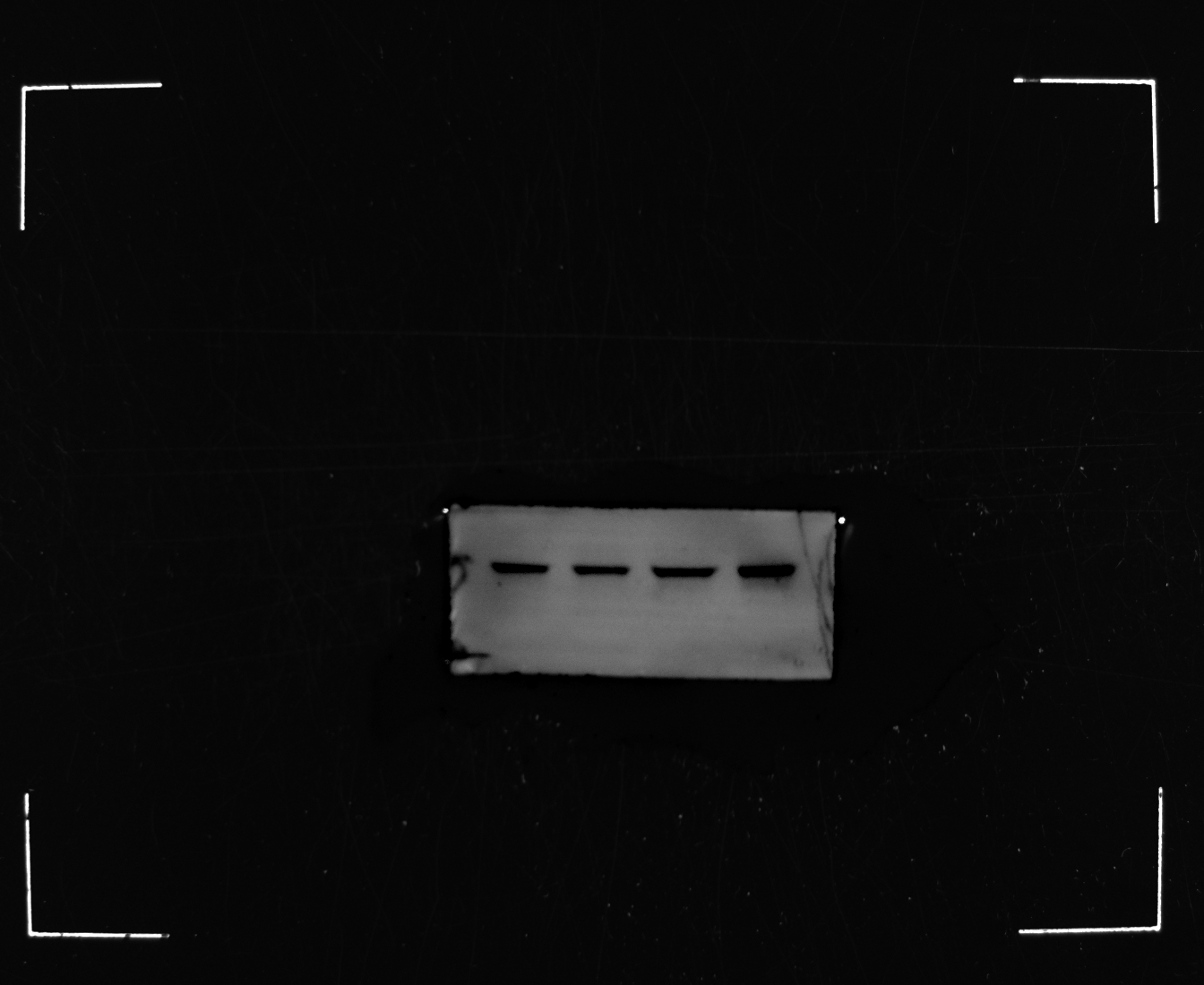


Cleaved-caspase3 -17kDa


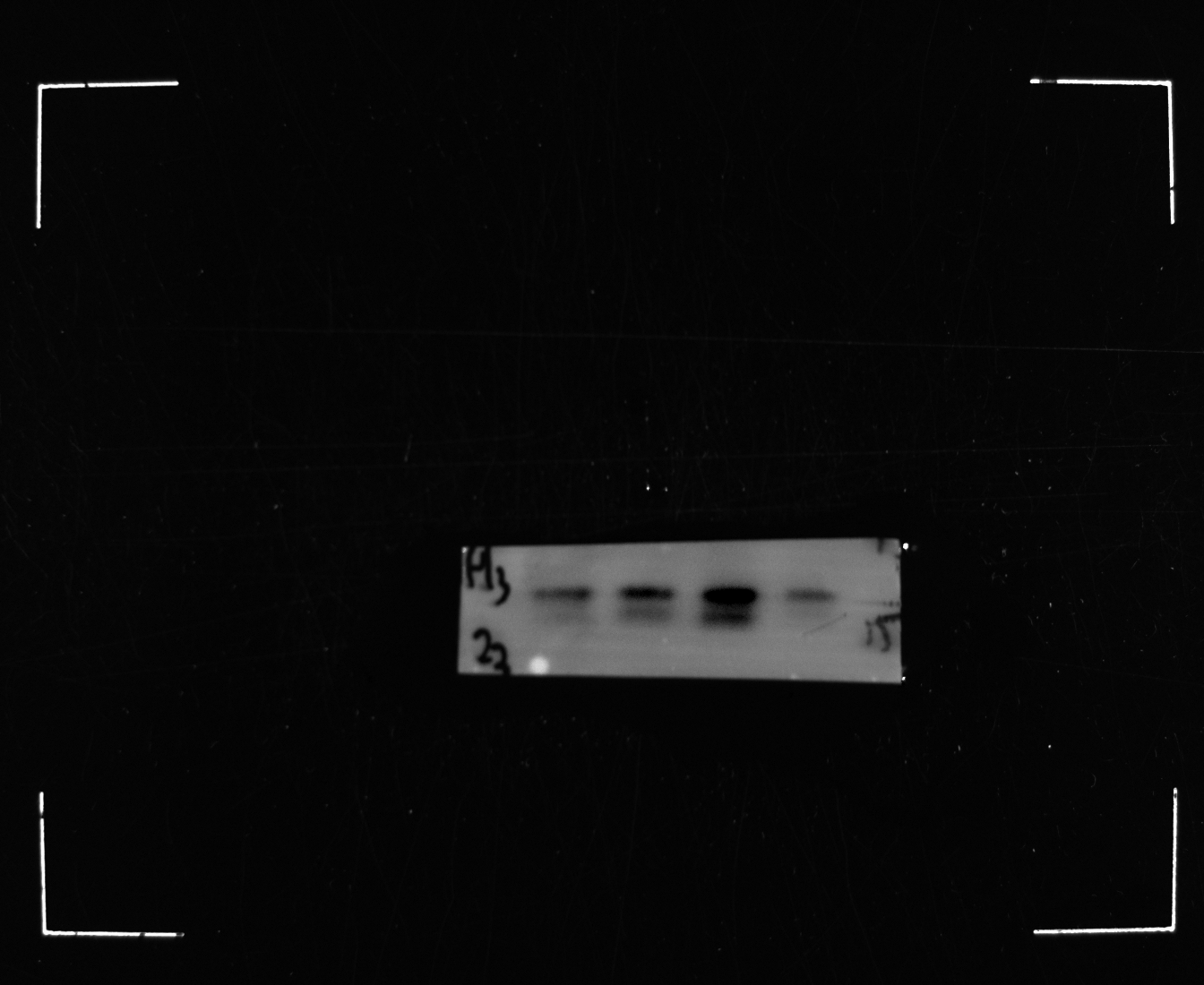


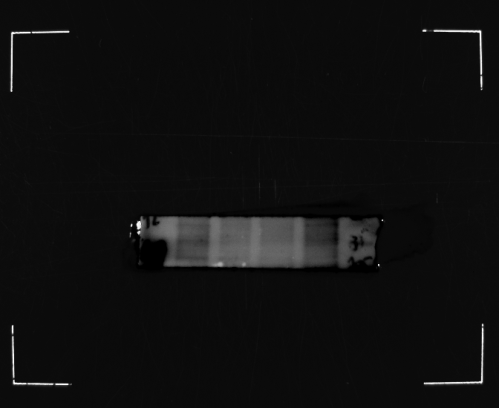


Cleaved-GSDMD -31kDa


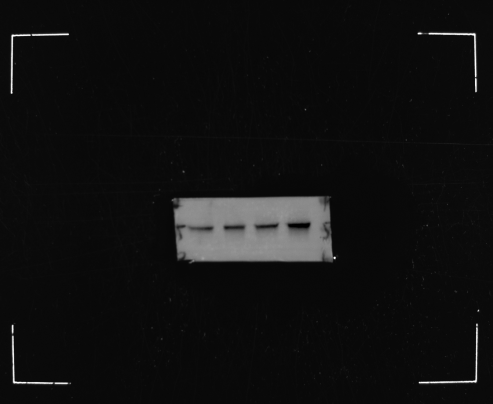


GSDMD -53kDa

GPX4 -17kDa


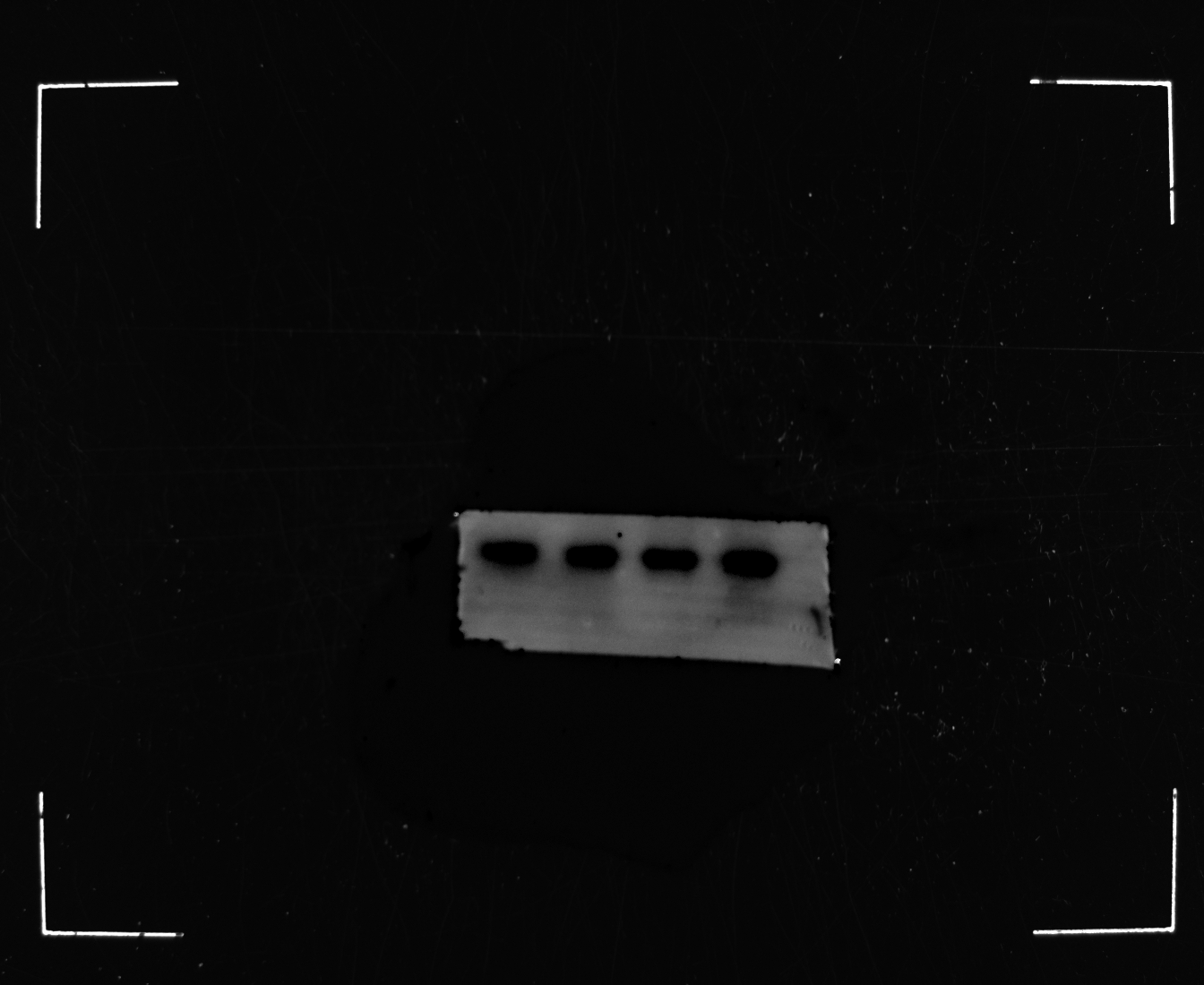


GAPDH -35kDa


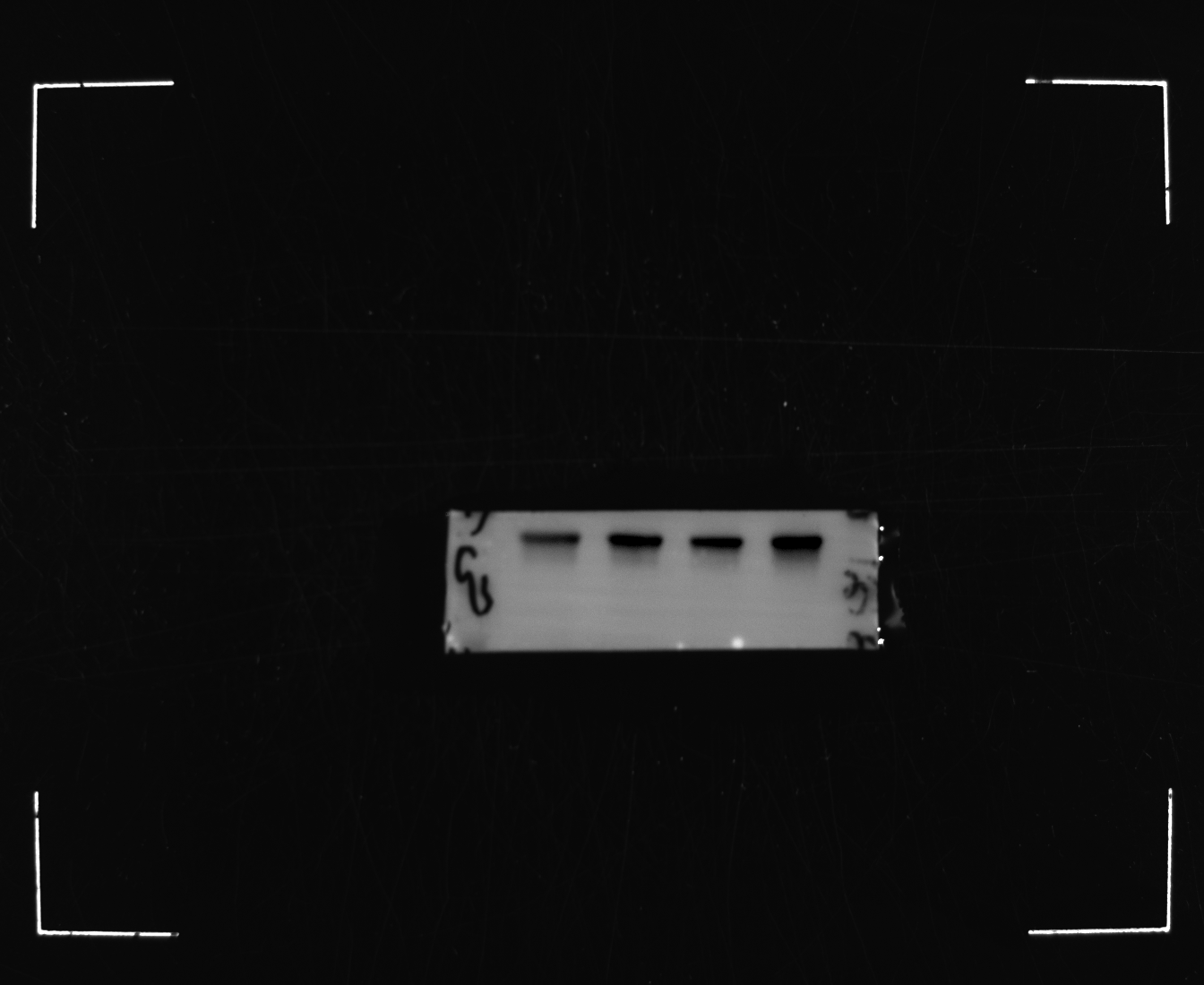


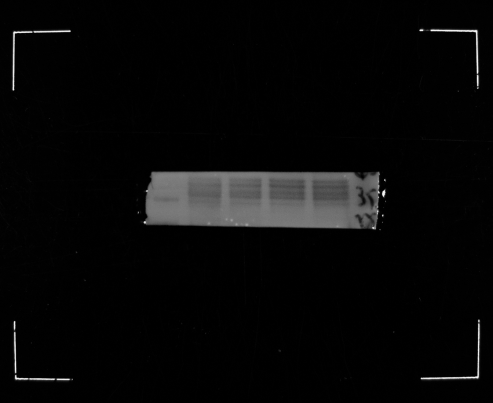


GAPDH -35kDa

**Supplementary Figure 4B**

Input

IP


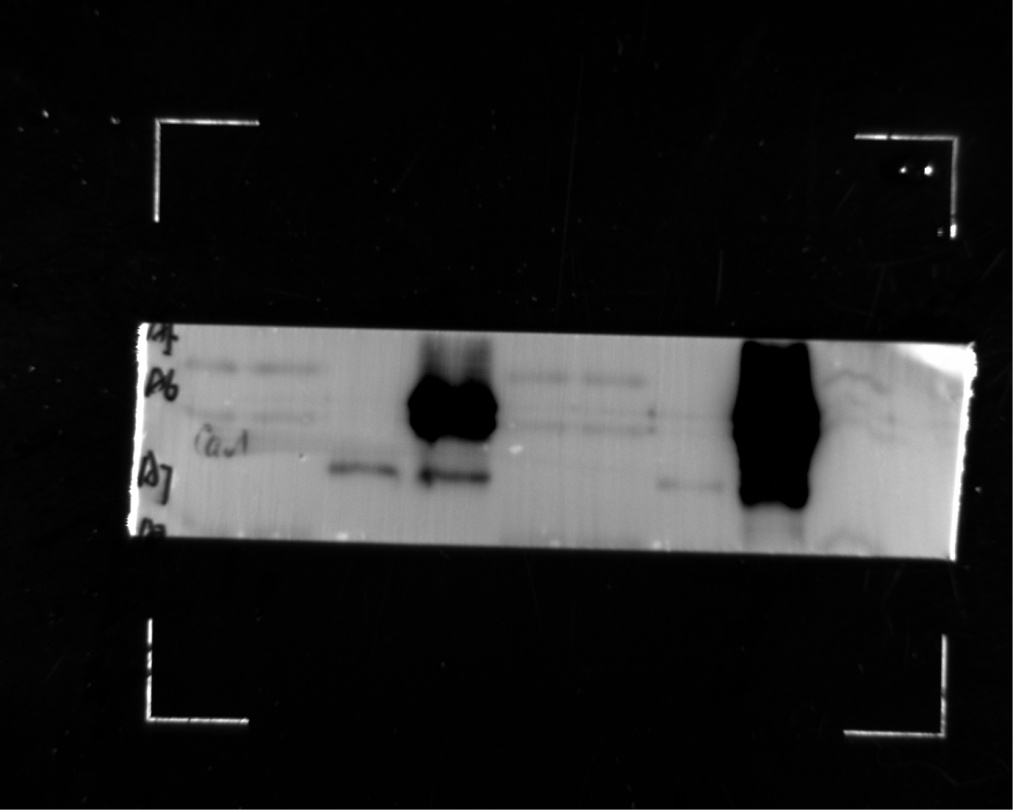


CAV1 -23kDa


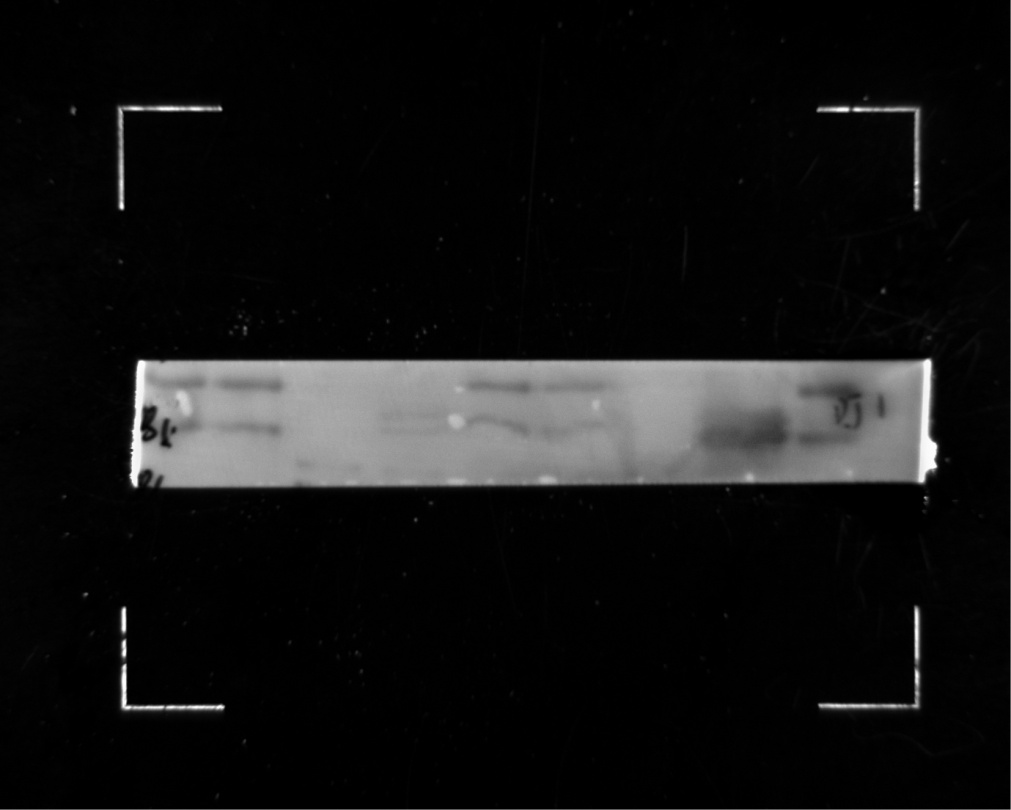


DJ-1 -21kDa


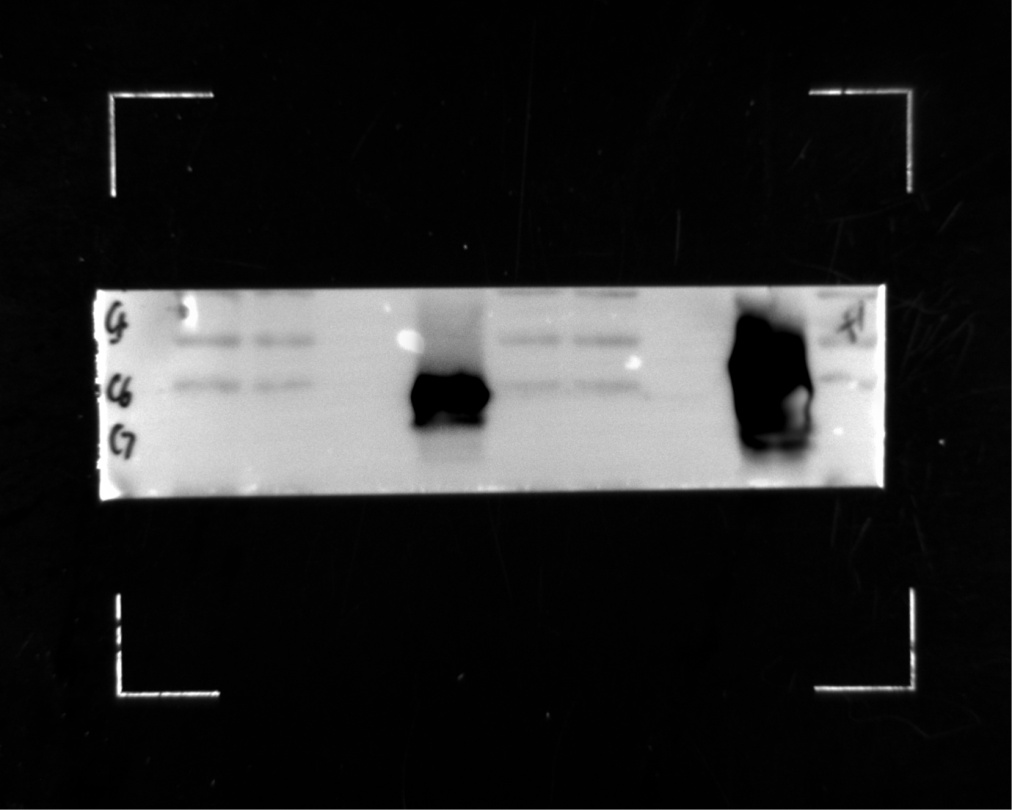


Flag -23kDa

**Supplementary Figure 4C**

**
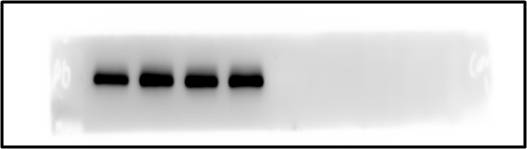
**

CAV1 -23kDa

**
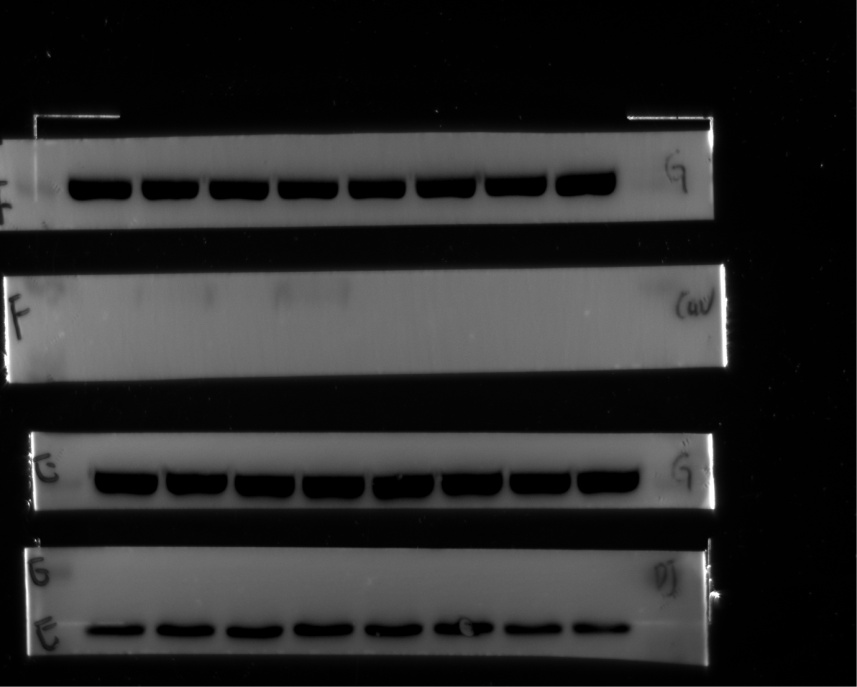
**

DJ-1 -21kDa

**
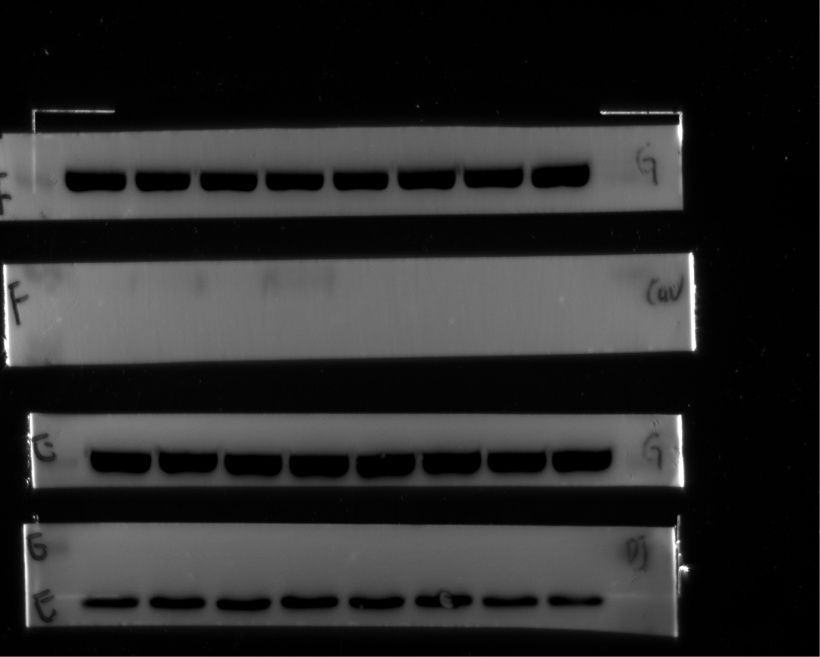
**

GAPDH -35kDa

**Supplementary Figure 4E**

Flag -23kDa


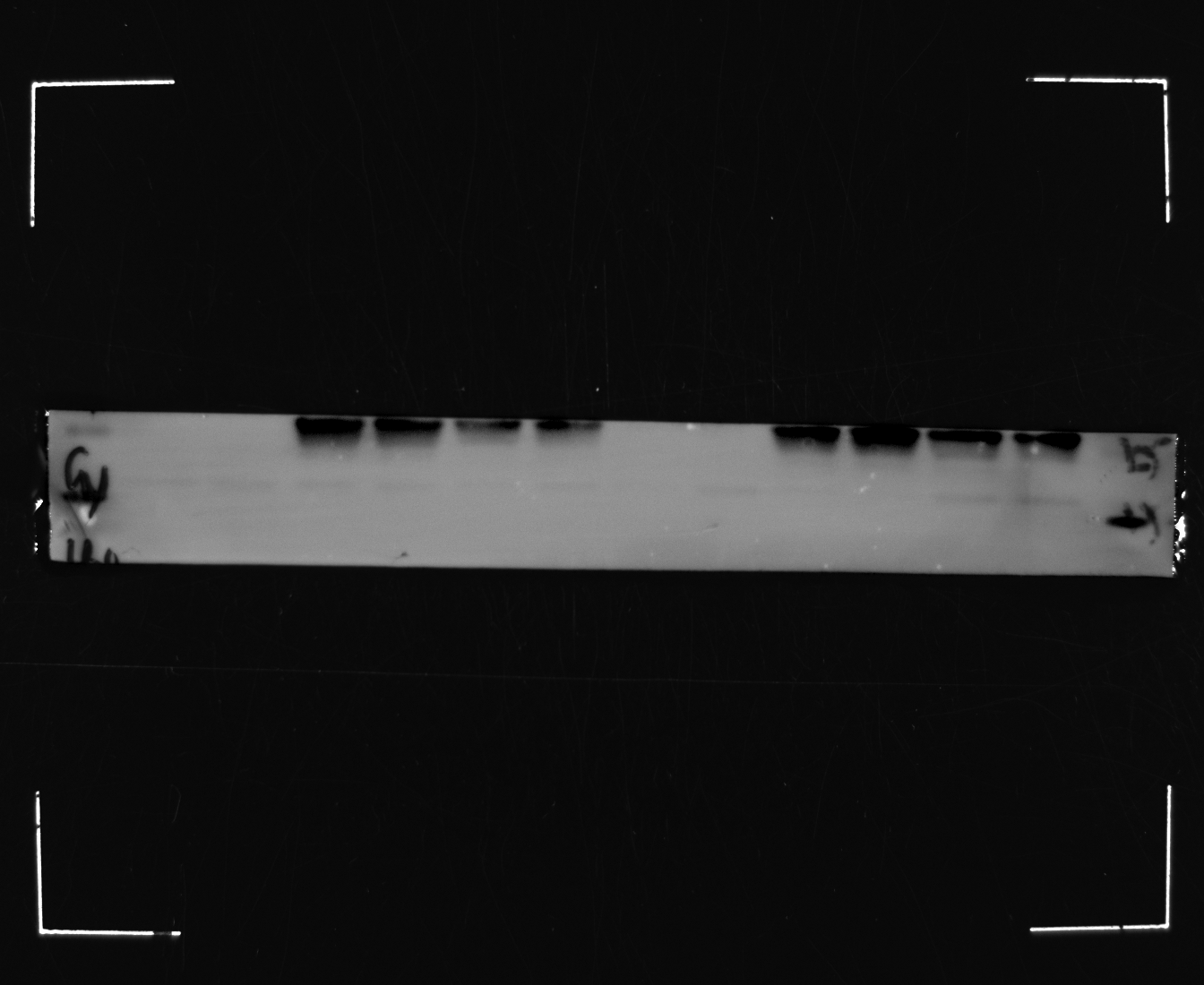


HA -21kDa


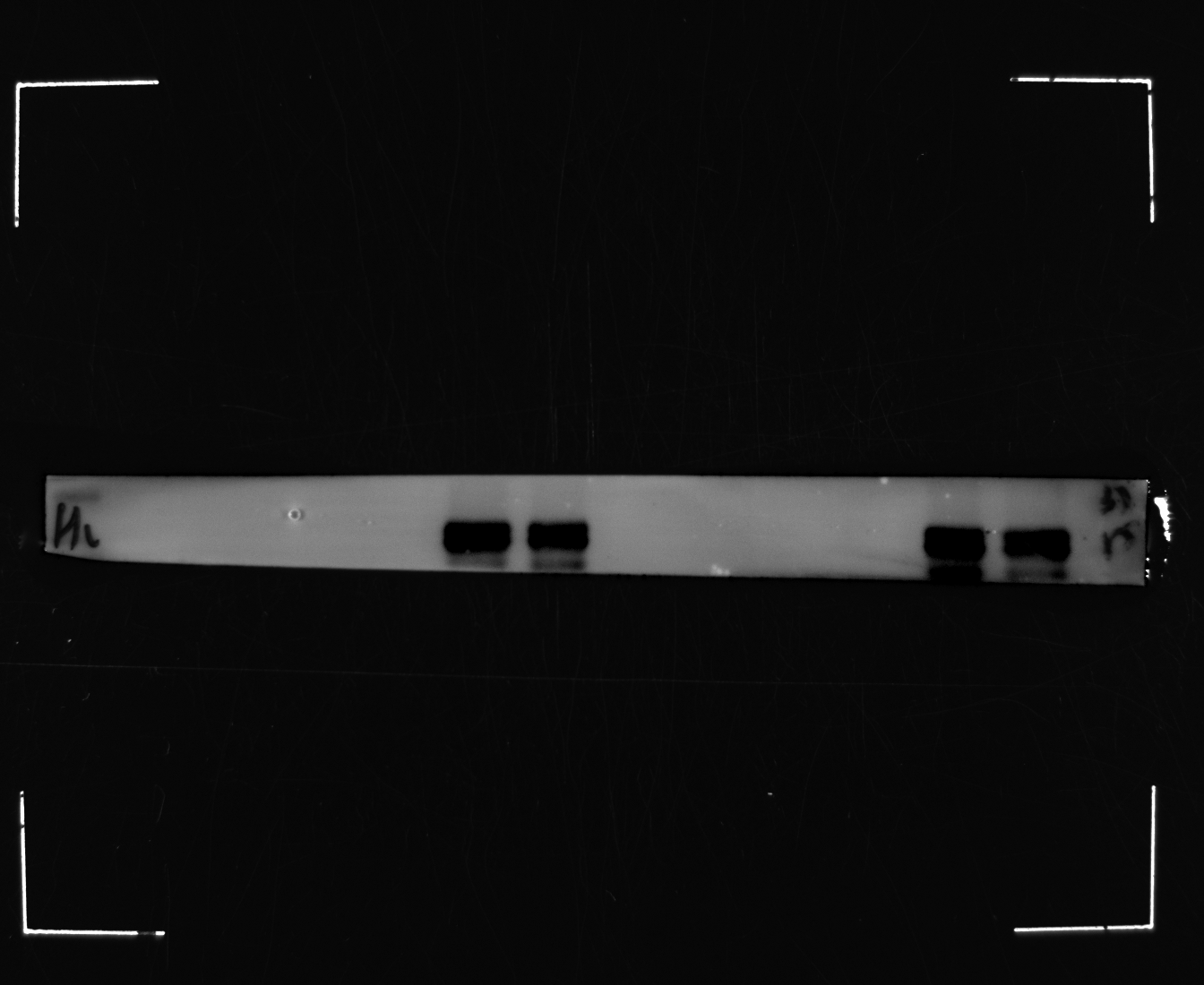


P62 -62kDa


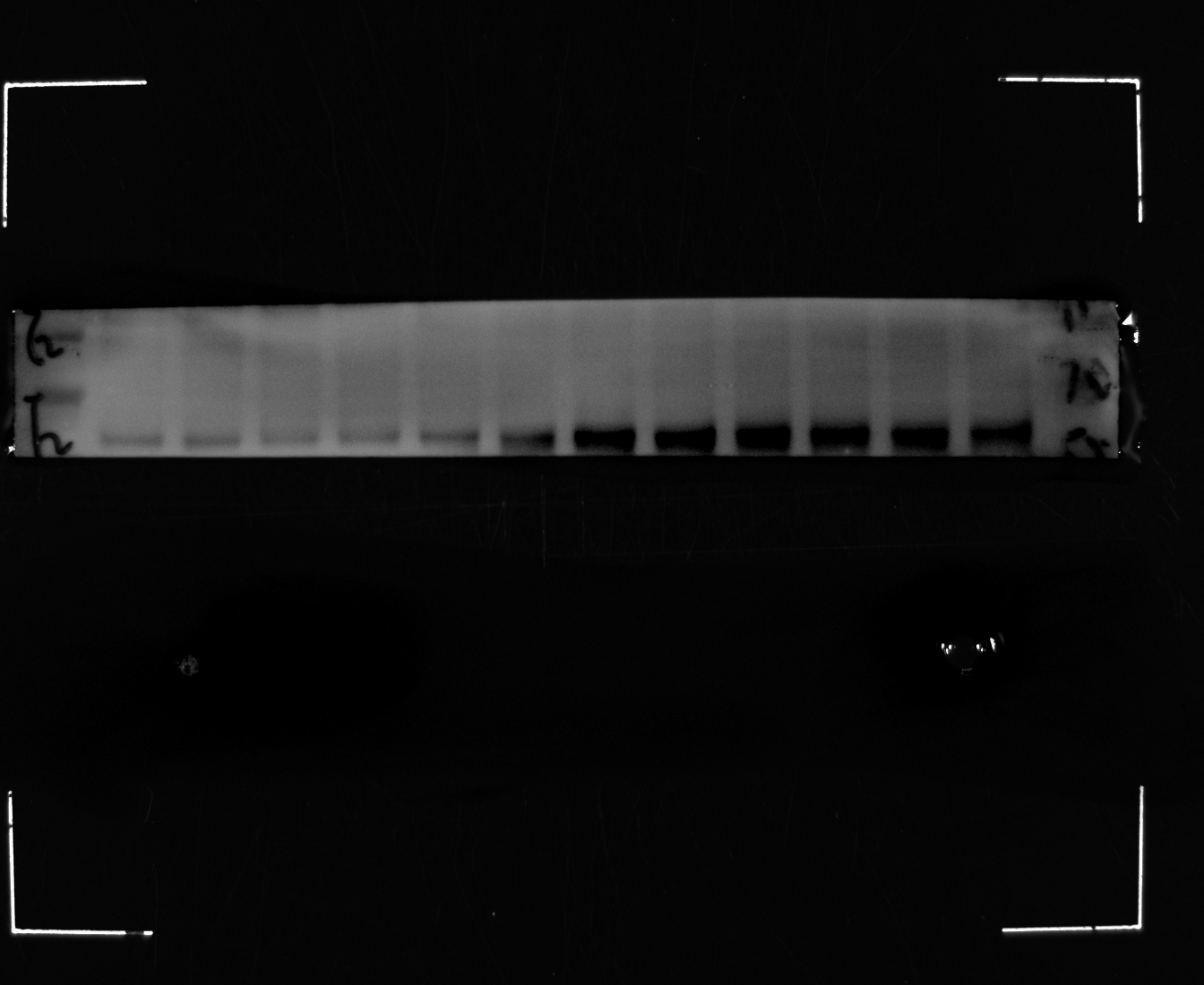


GAPDH -35kDa


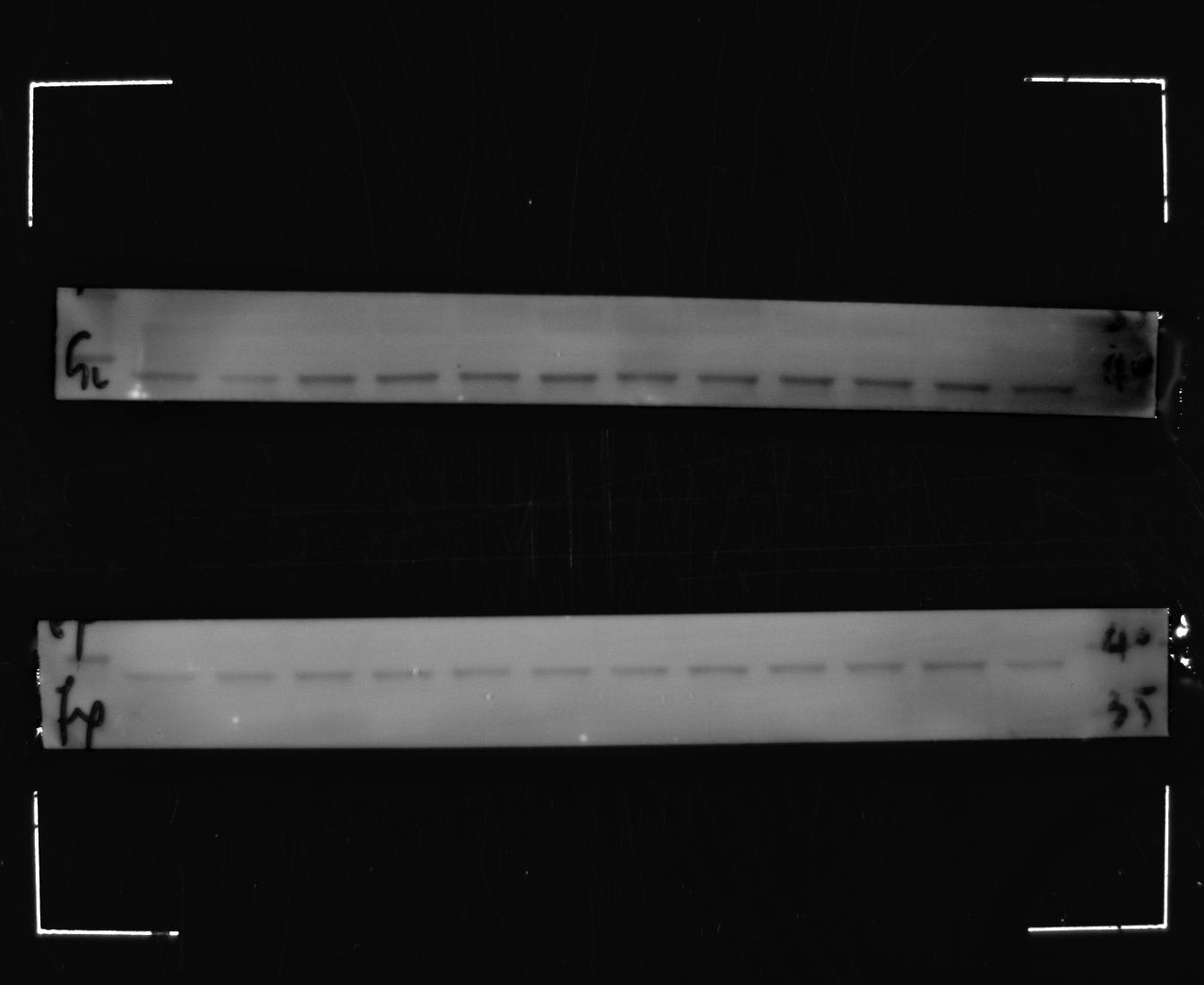


**Supplementary Figure 4F**

Flag -23kDa


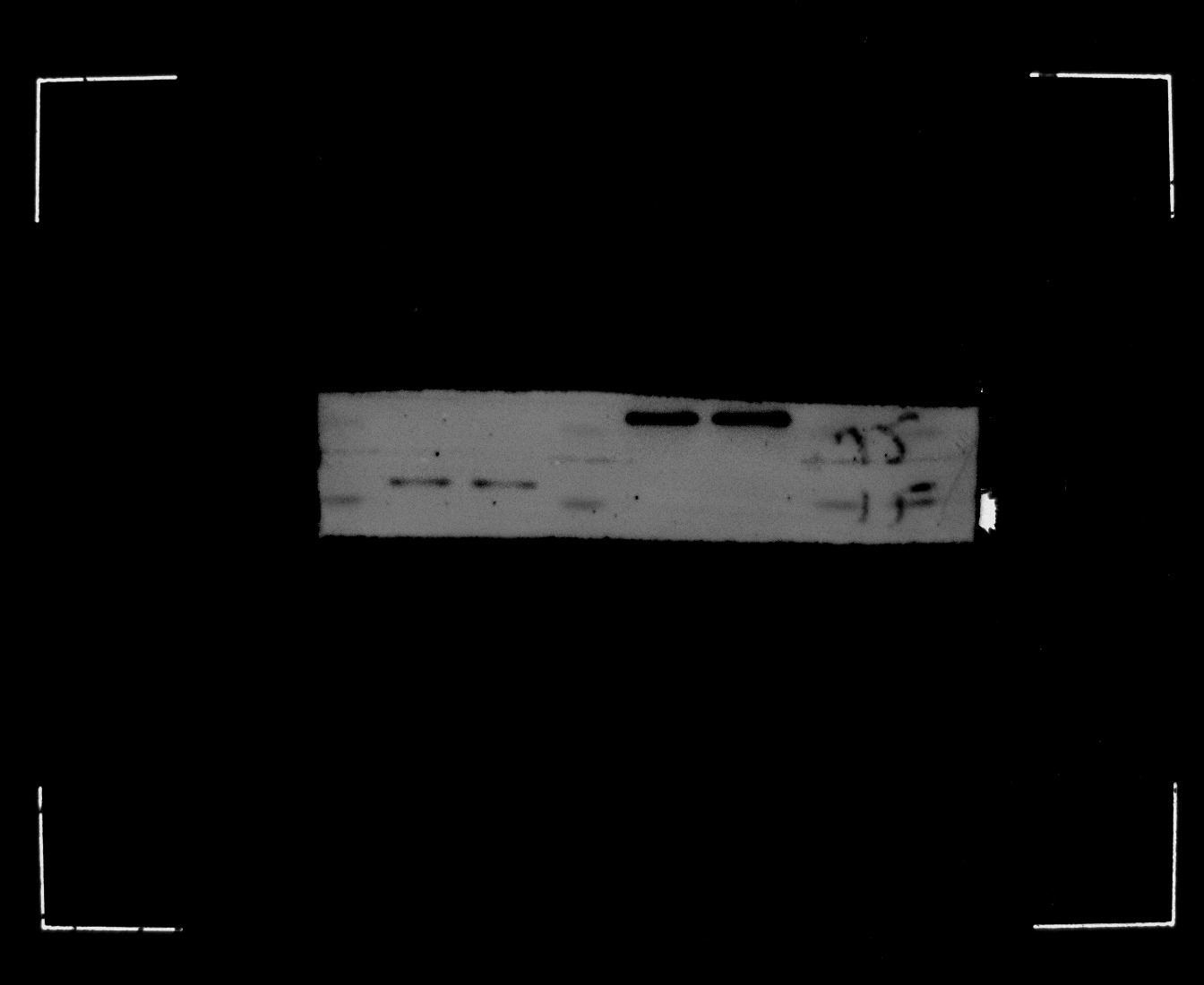


Ubiquitin


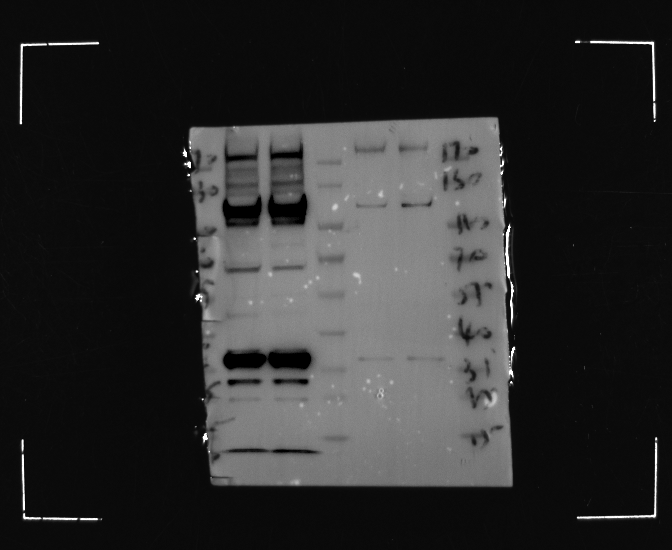


GAPDH -35kDa


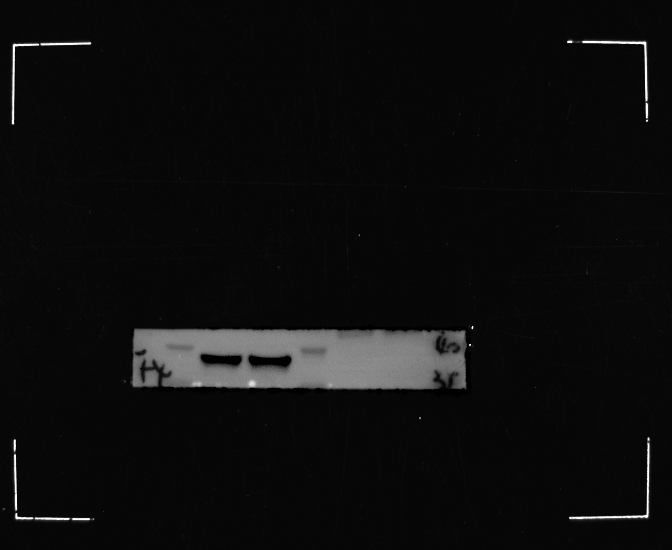


**Supplementary Figure 7C**

**
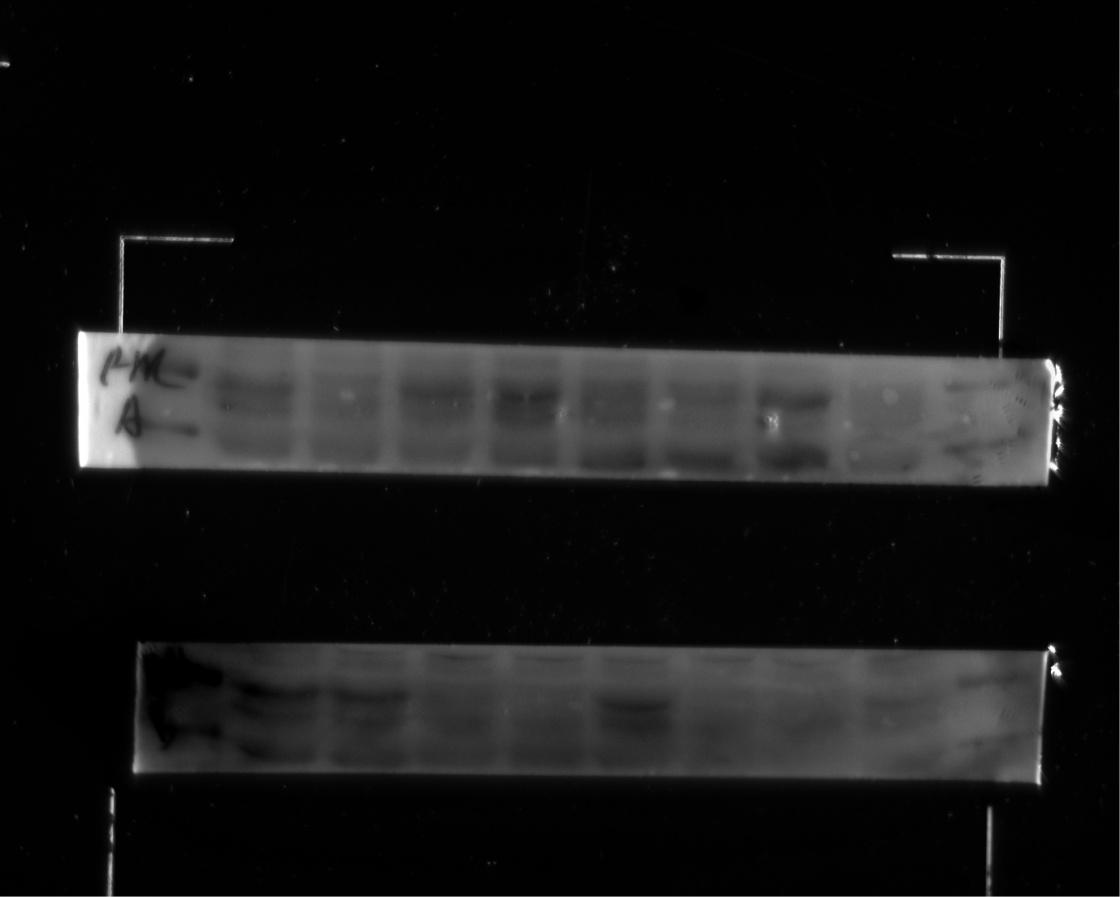
**

p-MLKL -54kDa

**
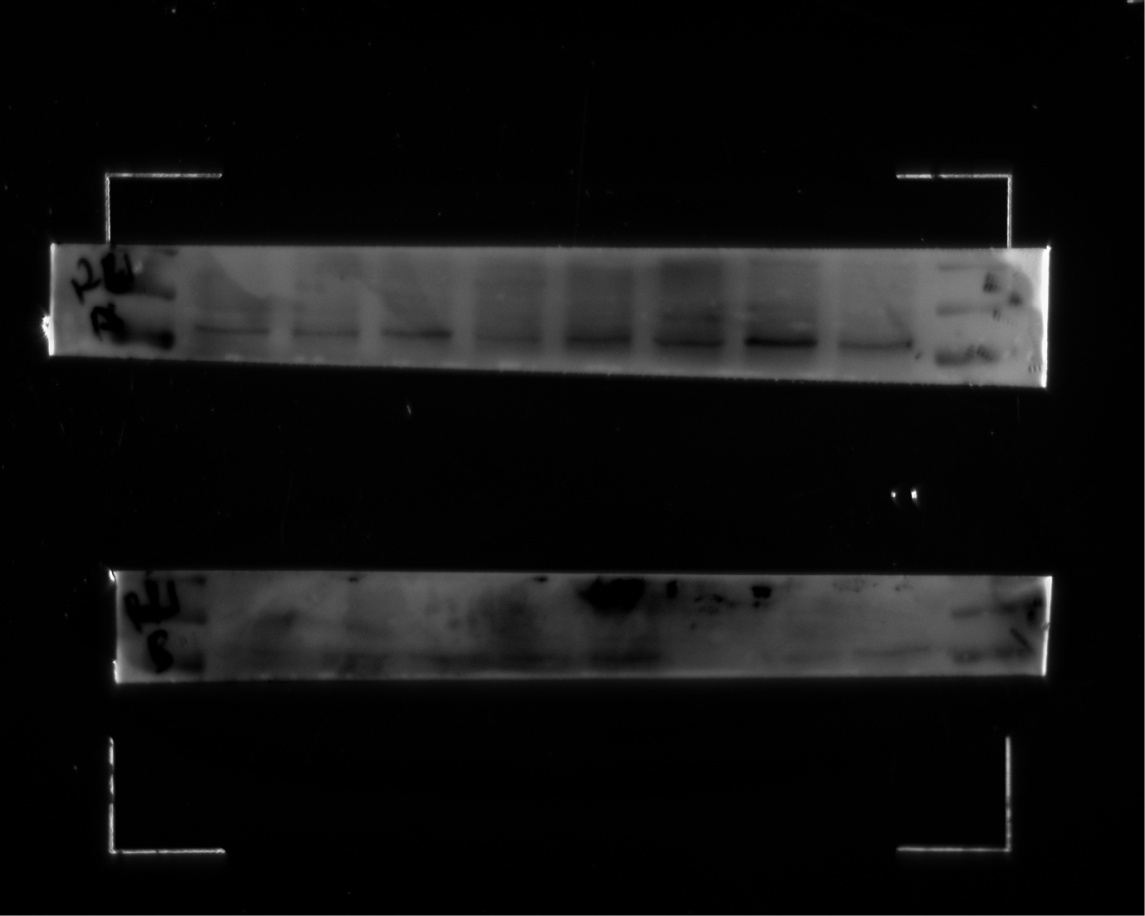
**

p-RIPK1 -78kDa

**
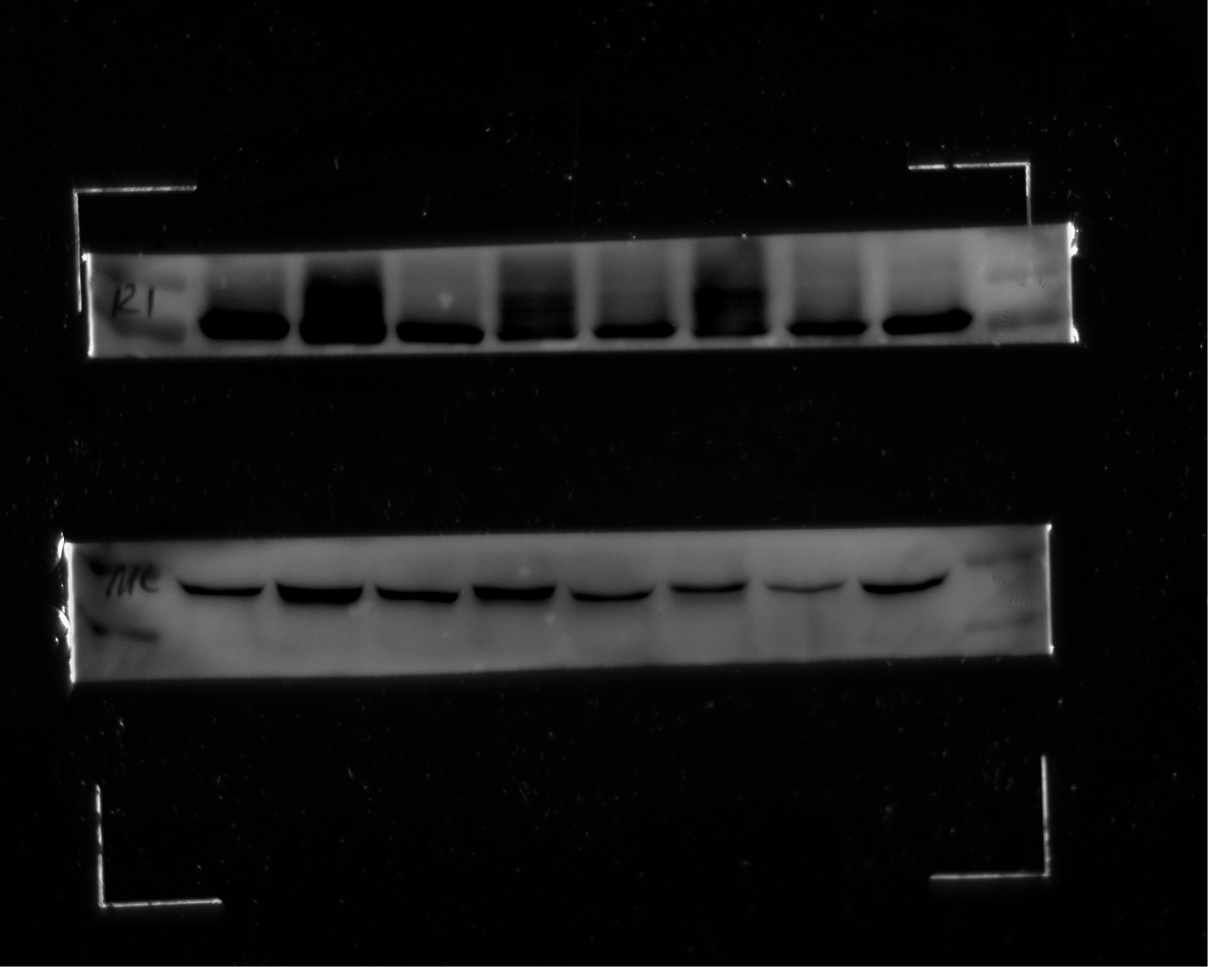
**

MLKL -54kDa

RIPK1 -78kDa

**
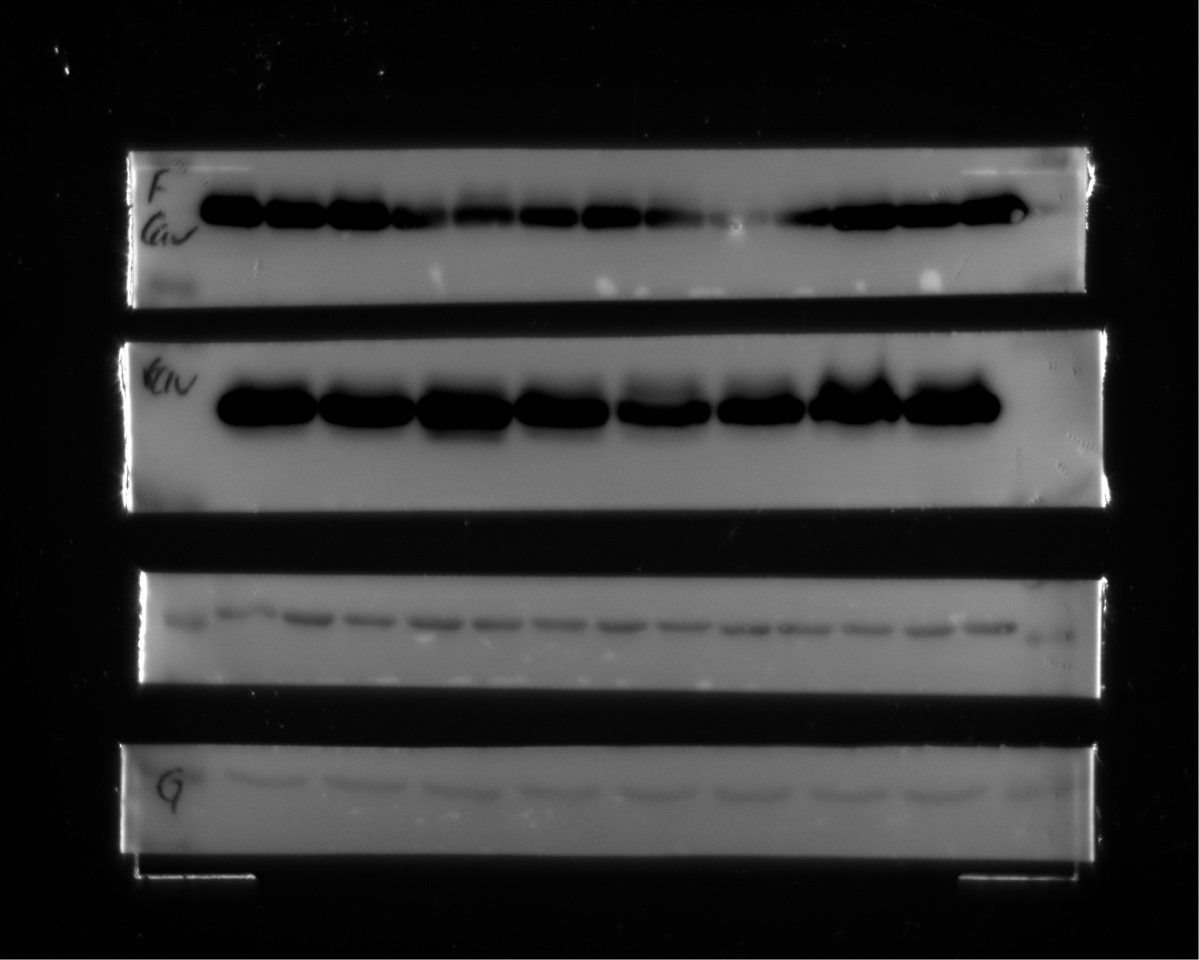
**

CAV1 -23kDa

**
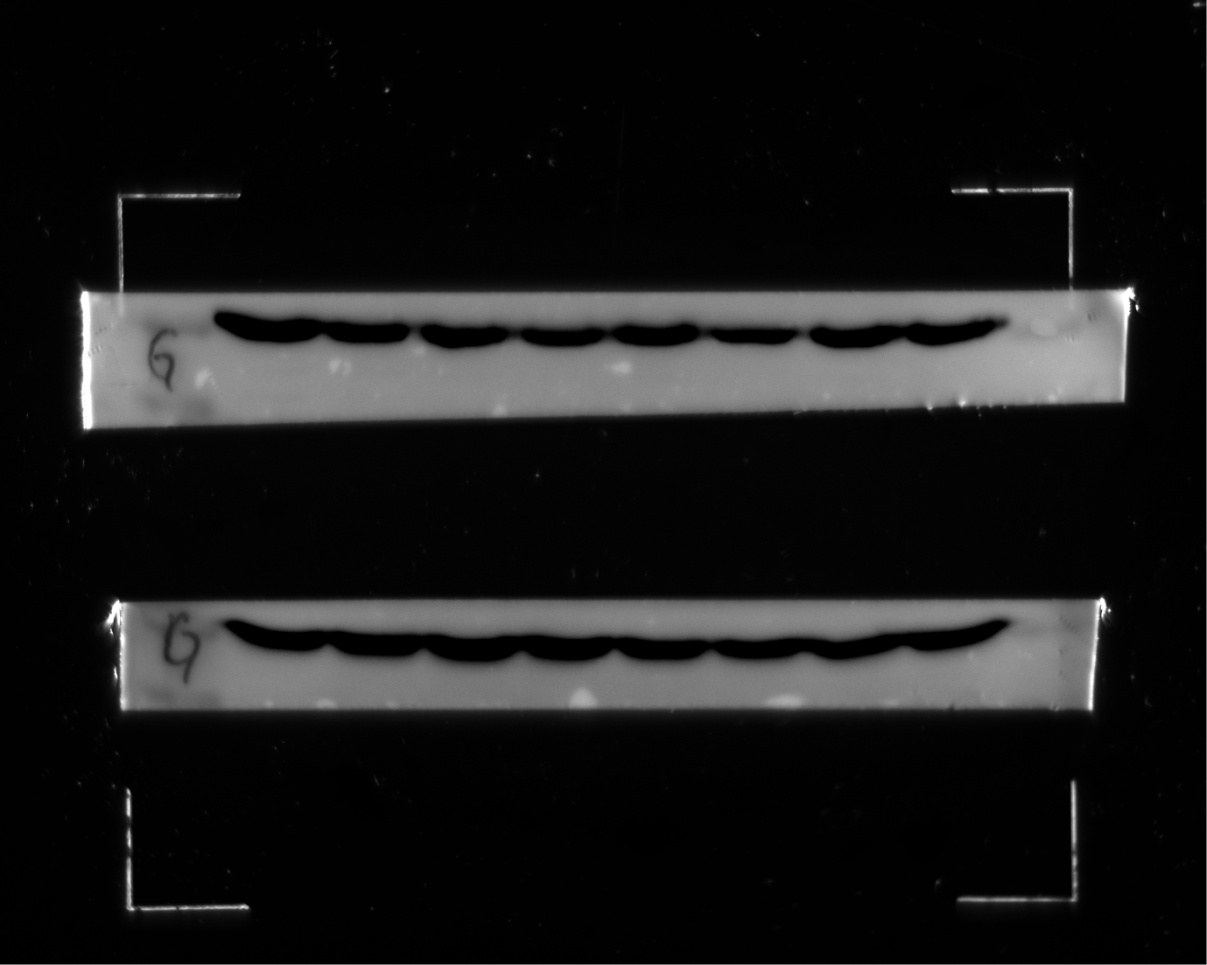
**

GAPDH -35kDa
